# Validation of *De Novo* Designs of Solid-Binding Peptides

**DOI:** 10.1021/acscentsci.5c02193

**Published:** 2026-05-22

**Authors:** Michael T. Bergman, Fiona Mukherjee, Yufan Feng, Carol K. Hall, Nicholas L. Abbott

**Affiliations:** † Department of Chemical and Biomolecular Engineering, 6798 North Carolina State University, Raleigh, North Carolina 27606, United States; ‡ Smith School of Chemical and Biomolecular Engineering, 5922 Cornell University, Ithaca, New York 14853, United States; § Department of Chemistry and Chemical Biology, Cornell University, Ithaca, New York 14853, United States

## Abstract

Solid-binding peptides (SBPs) are versatile molecules that can control a range of atomic-scale interfacial processes, but they remain challenging to discover. Current approaches for discovery rely on directed evolution, which samples only a small fraction of possible sequences. Data-driven methods for therapeutic peptides are also not applicable as they rely on crystal structures whereas peptides adopt varied conformations at solid surfaces. To address this challenge, we recently combined biophysical modeling and machine learning to design plastic-binding peptides that were predicted to have strong adsorption enthalpies. Here, we evaluate these designs using steered molecular dynamics and single-molecule force measurements and identify *de novo* designed peptides that bind strongly to polyethylene. Experimental adhesion forces exceed those previously reported for SBPs, and adsorption free energies from metadynamics simulations support strong binding. Analysis of the designed peptides reveals blocks of non-polar and charged residues, which enables them to adopt conformations that segregate non-polar amino acids to the plastic surfaces (generating hydrophobic interactions) and charged amino acids away from the surfaces. The contact patterns within the non-polar blocks depend on sequence context and polyolefin type. Overall, we validate a general approach for *de novo* SBP discovery that has broad scientific and engineering applications.

## Graphical abstract

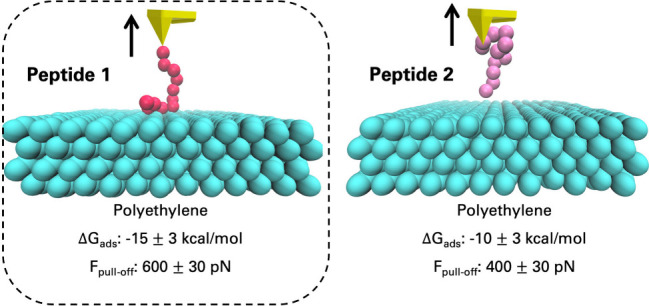

## Introduction

Solid-binding peptides (SBPs) can be used to tailor the interfaces of inorganic, synthetic, and biological materials, making them powerful tools for biotechnology, medicine and materials science. For example, they can be used to modify the surface properties of solids (e.g., wettability or tendency to nonspecifically adsorb biomolecules) to increase their biocompatibility, reduce biofouling, and modulate catalytic activity.^1^ Additionally, SBPs can guide the growth of inorganic crystals by influencing the energetics of specific crystallographic facets.^2,3^ Finally, and most relevant to our work, SBPs can recognize and selectively bind^4^ to the surfaces of targeted metals,^5^ metal oxides^6^ and synthetic polymers (with desired tacticity).^7,8^ In all these contexts, SBPs adhere to materials through a complex interplay of noncovalent interactions, including hydrophobic, electrostatic, hydrogen bonding, π-stacking, and van der Waals interactions.^9–11^

How are SBPs discovered? Nearly all SBPs reported previously have been identified via high-throughput screening (HTS) methods such as phage display^12^ or directed evolution.^13^ While HTS has identified SBPs for diverse materials,^4,12,13^ it has limitations. For example, HTS typically samples up to 10^9^–10^10^ peptides, which is a small fraction of the possible peptide sequences (e.g., 10^15^ for a 12-residue peptide); superior SBPs likely remain undiscovered. HTS also typically yields minimal insight into why a discovered SBP has high affinity for a given material.

To address these limitations, we recently reported SBPs designed by biophysical modeling and machine learning (ML).^14,15,56^ These new methodologies for SBP design are inspired by the successful use of related tools for identification of new peptide-based drugs^16^ and biomaterials,^17^ as well as small molecule drugs^18^ and proteins.^19^ In these contexts, ML tools permit 1) intelligent sampling of the massive design space using physical laws or patterns learned from large experimental databases, such databases containing protein/peptide crystal structures; 2) targeting of desired peptide properties by altering the optimization goal; and 3) iterative improvement via refinement of the modeling rules (i.e., the sequence – function relationship). However, biophysical and ML methods developed for designing peptide drugs cannot be directly translated to SBP discovery because experimental structural or thermodynamic databases for peptides at solid interfaces either do not exist or are small and challenging to obtain. In particular, peptides typically adopt a diverse distribution of conformations on solid surfaces,^20,21^ leading to complex sequence-structure relationships.^22^ The few examples of the use of biophysical modeling and/or ML for SBP discovery that have been reported are based solely on optimization of amino acid compositions (and not conformations) for a specific target.^15^ Other computational approaches for SBP discovery only qualitatively predict if a peptide binds to a material.^23,24^

In this paper, we develop a methodology for critically evaluating SBPs that combines molecular simulation and single molecule experiments, and use the methodology to assess recently reported SBPs designed to bind to polyethylene.^14,15,56^ The first set of SBP designs that we evaluate were predicted using biophysical modeling to quantitatively describe peptide affinity to a solid by generating a vast ensemble of adsorbed conformations, searching for stable adsorbed conformations within the ensemble, and then optimizing for amino acid sequences that preferentially adopt these favorable conformations.^14,25^ The second set of designs of SBPs that we evaluate emerge from ML models trained on the biophysical modeling data of the first methodology. The ML models learned sequence – function relationships that were subsequently used to guide searches for improved SBPs.^15,56^ For both approaches, we use MD simulations to evaluate the most promising SBPs, quantify peptide affinity, and provide atomic-scale insights regarding the adsorbed conformational states.^6,26^ We also perform experimental single molecule force measurements (SMFM) to validate the findings of the MD simulations and, more broadly, the SBP designs, an important step given the known limitations of MD simulations^27^ (e.g., the inability to sample beyond microsecond time scales, and necessary simplifications in models of solid interfaces and force fields). Our choice of SMFM for the experimental validation of SBPs was inspired by past studies that have quantified adhesive interactions between peptides and surfaces at the single-molecule level.^28–32^ In addition to using adhesion force measurements to validate *de novo* SBP designs, we also use SMFM to elucidate the contributions that key interactions (e.g., hydrophobic interactions) make to measured peptide affinities.

We report evaluation of *de novo* designs of SBPs for polyethylene, a major component of micro- and nanoplastics (MNPs) pollution. Our focus on such peptides is motivated by their potential to address MNP^33^ pollutants^34,35^ found in the environment,^36–42^ such as by detecting MNPs via peptide-based biosensors,^43^ capturing or filtering MNPs via bioflocculation,^44^ or helping plastic-degrading enzymes or microorganisms adhere to their target plastic.^45^ We find that both MD simulations and SMFM validate the designs of the polyethylene-binding peptides that emerge from the SBP-discovery methodologies described above and yield atomic-scale insights into the origins of the high binding affinities. In particular, we report the validation of one peptide that adheres very strongly to polyethylene (and polypropylene), as indicated by experimentally measured adhesion force magnitudes that are higher than typical unfolding forces reported for most mechanically stable proteins and protein–protein interactions.^46–49^ Overall, our results provide strong validation of new methodologies for *de novo* design of SBPs, methodologies that have the potential to be applied to a range of contexts including advanced materials science, healthcare and biotechnology.

## Methods

### Procedure for Designing and Validating Solid-Binding Peptides

We evaluate SBPs that were recently designed for polyethylene^14,15,56^ using the methodologies shown in the top row of Figure 1. Key features of the design methodologies are described here, with details presented in SI Sections 1 and 2. Briefly, the first methodology is based on biophysical modeling. It uses the PepBD algorithm to predict SBPs with strong affinity for a targeted plastic surface.^14,50^ PepBD combines Monte Carlo sampling of amino acid sequences and adsorbed peptide conformations with the molecular mechanics and generalized Born and surface area solvation (MM/GBSA) model^51^ to find strong binders (i.e., preferred PepBD score). With this procedure, we evaluated the affinity of 700,000 unique amino acid sequences over 100 unique adsorbed conformations. This approach optimizes peptide adsorption enthalpies while neglecting the role of conformational entropy and using a simple model to capture solvent effects. We discuss how this may bias peptide selection in the SI Section 2. The second general approach involves the application of two machine learning (ML) methodologies to conduct additional searches for improved SBPs. Both ML approaches involve two steps: the first step is training on PepBD data to predict the PepBD score for an amino acid sequence, and the second step involves a search for new peptides with improved affinity based on the score predictions of the first component. In the first ML approach,^56^ a long–short-term memory (LSTM)^52^ network predicts peptide affinity, and Monte Carlo Tree Search (MCTS)^53^ searches for high affinity peptides. In the second ML approach,^15^ a dense neural network with an evidential deep learning (EDL)^54^ layer predicts peptide affinity, and the biased random-key genetic algorithm^55^ searches for high affinity peptides. In this paper, we term the first and second models as “MCTS” and “EDL”, respectively. Detailed descriptions of the training strategy, model accuracy, and peptide generation strategy for the EDL-based method can be found in Figures 1, 2, and 3 of a prior publication,^15^ and for the MCTS-based method can be found in Figures 1, 2, 4, S1, S13, and S14 of ref 56. The two ML methods have different strengths: MCTS readily optimizes multiple peptide properties (e.g., affinity and predicted solubility), while EDL prioritizes peptides for which it can confidently predict the PepBD score without specifically optimizing for water solubility. Overall, each of the SBP design methodologies (PepBD, MCTS, and EDL) generate tens to hundreds of SBPs with high predicted affinity for each type of plastic.

**Figure 1:**
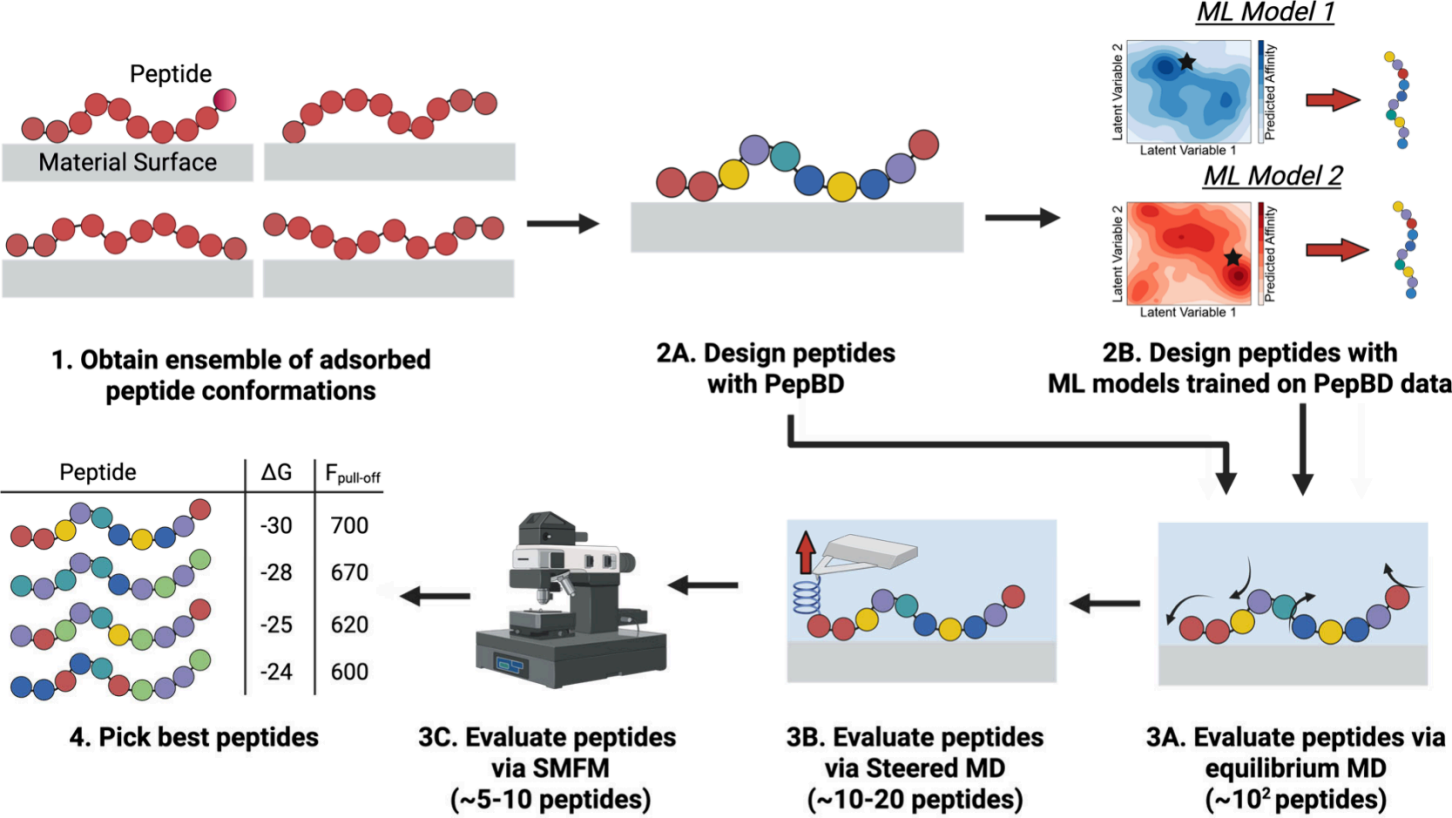
Overview of computational design and evaluation of solid-binding peptides. (Top) Starting with a 3D structure of a peptide adsorbed to a solid surface (e.g., polyethylene), PepBD identifies amino acid sequences with high predicted affinity for the solid via biophysics-based peptide design. The resulting data is used to train machine learning (ML) models that search for novel peptides with high predicted affinity for the solid. (Bottom) The best PepBD and ML peptides are evaluated in three stages. In the first stage, equilibrium MD simulations provide a computationally cheap and rapid evaluation of peptide affinity to the solid. In the second stage, the best peptides are evaluated more rigorously in simulation using steered molecular dynamics (SMD). In the third stage, the best peptides are evaluated in experimental single-molecule force measurements (SMFM). The peptides with the best SMFM results are identified as the lead candidates. Figure made with Biorender.

The methodology that we report in this paper to evaluate the designed SBPs is shown in the bottom row of Figure 1; quantitative metrics that describe the computational resources and methods are reported in the SI data file. In the first step, the peptide adsorption free energy (ΔG_ads_) was evaluated using the MM/GBSA method^51^ over an ensemble of states generated from explicit-solvent equilibrium MD simulations^15,56,57^ (SI Sections 3 and 4). This methodology can evaluate up to 100 peptides per plastic at a reasonable computational cost (Figure 1panel [Fig fig3]A). However, MM/GBSA uses an implicit solvent-based model to calculate the free energy of adsorption and approximates the loss in peptide conformational entropy upon adsorption using normal-mode analysis.^58^

The best-performing 20% of peptides from MM/GBSA-based evaluation proceeded to a second screening based on steered molecular dynamics (SMD) simulations (Figure 1, panel [Fig fig3]B).^59^ SMD calculates ΔG_ads_ by performing many nonequilibrium simulations, in which a peptide is desorbed from the solid by applying an external bias (schematic illustration in Figure 2A, see SI Sections 5 and 6 for details). Re-evaluating peptides with SMD is useful because it avoids approximations inherent in MM/GBSA (see SI Section 7). Each SMD simulation was started from one of 16 unique adsorbed conformations, where each conformation was equilibrated and then desorbed three (polyethylene) or six (polypropylene) times. Fewer simulations were performed for polyethylene as ΔG_ads_ did not change significantly when increasing from 3 to 6 simulations per conformation (Table S1). The force and then cumulative work due to the external bias was calculated (Figure S1), with different simulations generating distinct force spectra as they sampled different desorption pathways (Figure S2). The distribution of desorption works obtained from all simulations were used to calculate ΔG_ads_ using the Jarzynski relation^60^ (example work distributions for peptides with relatively low, moderate, or high affinity are provided in Figure S3). Our SMD protocol aimed to balance precision and throughput: precise calculations of ΔG_ads_ requires many SMD simulations,^61^ but more simulations means fewer peptides can be evaluated. The above procedure enabled evaluation of 10–20 peptides at a reasonable computational cost (250 – 500 ns of simulation time per peptide). To estimate the error, each ΔG_ads_ calculation was repeated twice for four different peptides on both polyethylene and polypropylene. The maximum difference in ΔG_ads_ between two runs over the four peptides was used as a conservative estimate of the uncertainty in ΔG_ads_ (see SI Section 6 and Table S2).

**Figure 2:**
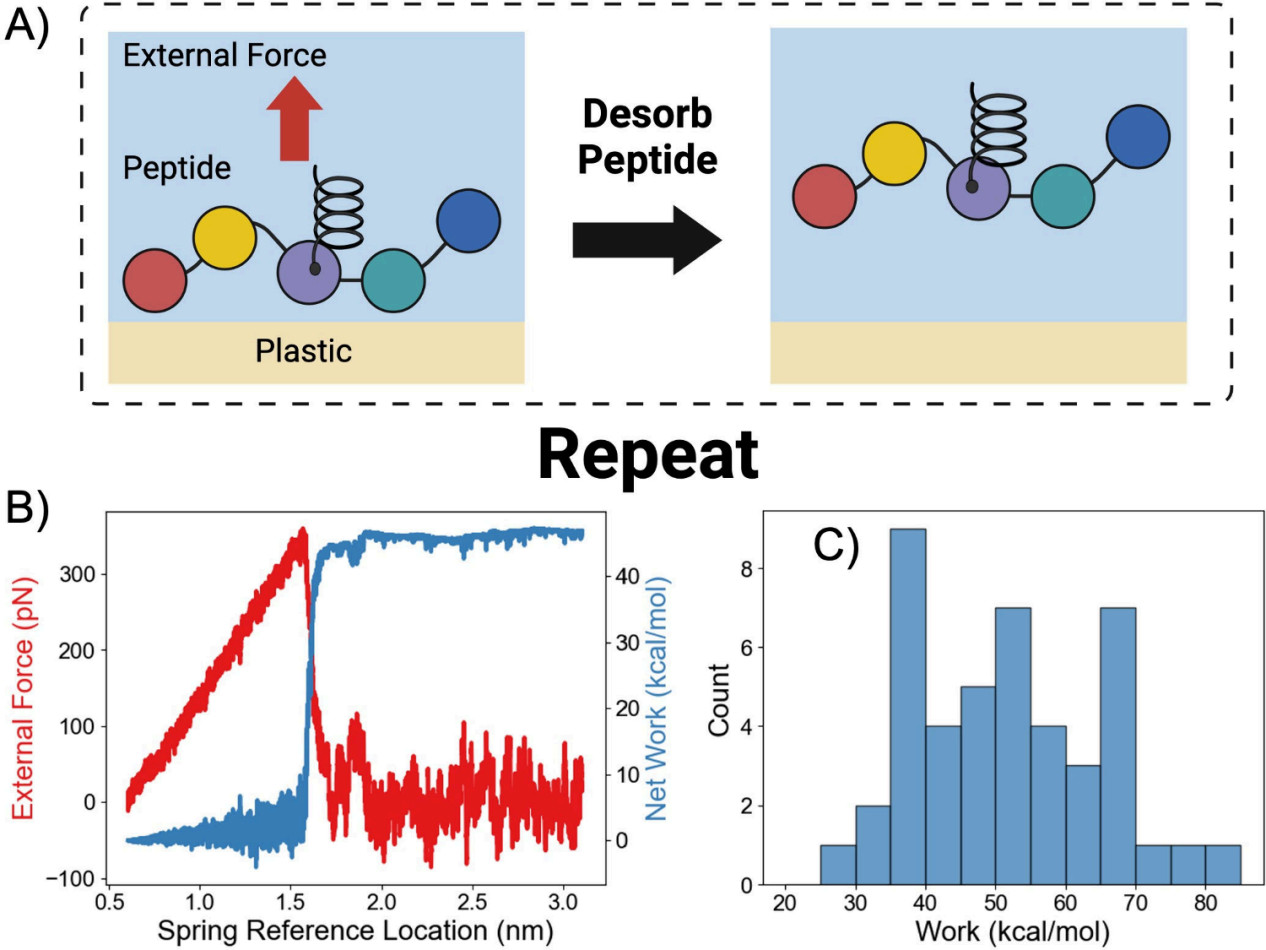
Overview of steered molecular dynamics (SMD). A) Schematic illustration of the SMD protocol. A peptide adsorbed to a plastic surface is desorbed by adding an external bias, in the form of a harmonic spring, to the peptide center of mass and pulling the spring away from the plastic surface. B) The work performed by the spring in each simulation time interval is the product of the applied force and the peptide displacement. C) Distribution of the work of desorption obtained by performing many SMD simulations for a given peptide-plastic pair.

**Figure 3:**
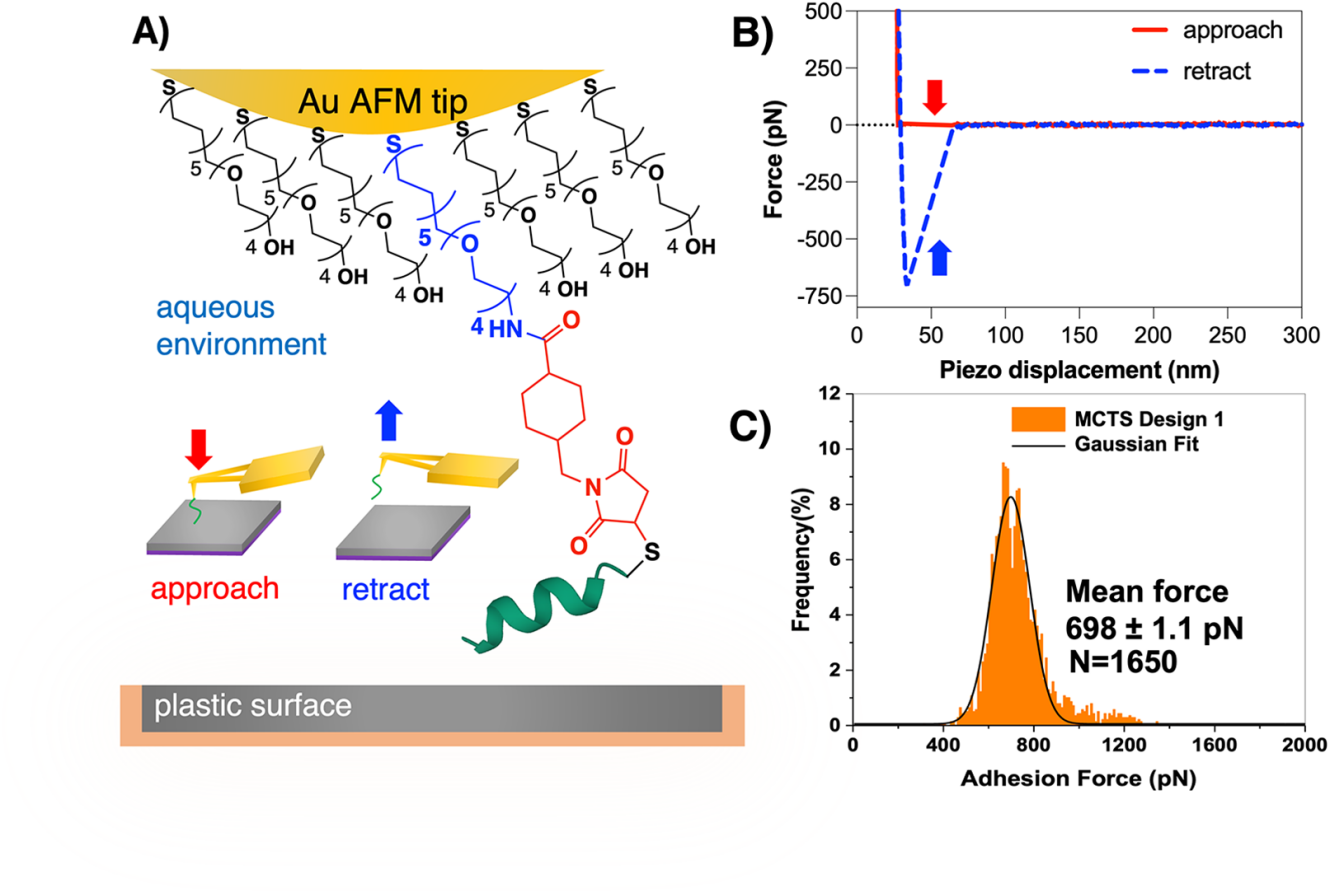
Single molecule force measurements, representative force–separation curves, and force histograms obtained to experimentally quantify peptide adhesion to a plastic surface. A) Schematic illustration of a candidate SBP covalently attached to an AFM tip. B) Plot showing a representative pull-off curve for a single pull-off event between a MCTS-designed peptide (MCTS 1) attached to an AFM tip and a polyethylene surface. C) Representative force histogram for a MCTS-designed peptide (MCTS 1) on a polyethylene surface. The measurements were performed in aqueous PBS. Control experiments that support our conclusion that the measured forces reflect single-molecule events are presented in Figure S5.

In the third evaluation step (Figure 1, panel [Fig fig3]C), all peptides evaluated in SMD simulations were evaluated experimentally using SMFM.^32,62^ SMFM measurements were performed by immobilizing each SBP sequence on the surface of the tip of an atomic force microscope (AFM; schematic illustration in Figure 3A) following a previously established protocol.^28–32^ A summary of experimental parameters for SMFM measurements is given in SI Section 8 and Table S3. We calculated statistical averages of adhesive forces over approximately 10^3^ pull-off curves, obtained using at least three independently prepared peptide-functionalized tips and plastic surface pairs (Figure S4, S7, SI Section 9; in addition, the SI data file shows the number of pull-off curves for each peptide). The peptides were immobilized on the AFM tips at a surface density of 0.1%, which we demonstrated permits measurement of adhesive interactions between single peptide molecules and plastic surfaces. Control experiments with peptide densities above and below 0.1% are provided in Figure S5 and Figure S8. Specifically, we found that lowering the peptide density beyond 0.2% did not change the measured force of adhesion, consistent with our conclusion that measurements performed at a peptide surface density of 0.1% reflect adhesive forces generated by single molecule events. Additional control experiments are reported in Figure S6. Across all control experiments, the adhesion forces measured were small compared to the forces generated by the interactions of SBPs with the plastic surfaces. Additional experimental details regarding the preparation and characterization of experimental surfaces can be found in the SI Sections 10 through 16, as well as Figure S11, S12 and Table S4.

## Results

### Initial Selection of Computationally Designed Polyethylene-Binding Peptides

The best *de novo* designs of SBPs for polyethylene that emerged from the biophysical modeling (PepBD^14^) or ML methodologies (EDL^15^ and MCTS)^56^ are compared in Table 1 using equilibrium adsorption free energies (ΔG_ads_) calculated from MM/GBSA simulations (Figure 1). Inspection of Table 1 (see also Table S5) reveals that designs from all three methodologies have more favorable adsorption free energies for polyethylene than random amino acid sequences (a reference for peptide affinity without design), with average MM/GBSA ΔG_ads_ values of −28.1 ± 4.4 kcal/mol, −32.9 ± 5.7 kcal/mol and −27.8 ± 3.5 kcal/mol for PepBD, MCTS and EDL designs, respectively, as compared to an average of −15.22 ± 7 kcal/mol for the random sequences. The average ΔG_ads_ does not vary significantly between the three design methodologies, but individual peptides vary substantially from the average ΔG_ads_ values for each methodology. The variability suggests that equilibrium MD (i.e., MM/GBSA) can discriminate between high and low affinity peptides. Guided by initial results obtained using the MM/GBSA-based method, six PepBD, two EDL, and two MCTS peptides were selected for further evaluation (Table 1), as described in the next section.

**Table 1:** *De Novo* Designed Plastic-Binding Peptides Evaluated in this Study

Description	Sequence	MM/GBSA ΔG_ads_ (±5.1 kcal/mol)^a^	Tested by SMD	Tested by SMFM
PepBD 1	HWMRTWRWHMFH	–36.8	Yes	Yes
PepBD 2	RWMWHMHRQTMW	–27.7	Yes	Yes
PepBD 3	MFWWRELHQQWR	–27.0	Yes	Yes
PepBD 4	MWMRHWRWHHFH	–25.9	Yes	No^b^
PepBD 5	HRWMMDHWRSFW	–25.8	Yes	Yes
PepBD 6	GFRHRIFWQHWW	–25.4	Yes	Yes
EDL 1	DWMWRREFFMHR	–25.4	Yes	Yes
EDL 2	HWMWRMKWNMRH	–30.3	Yes	Yes
MCTS 1	KWFFEKWWMMRR	–37.0	Yes	Yes
MCTS 2	RWRYDYWWMIFF	–28.9	Yes	Yes
Polyglutamic Acid (Control)	EEEEEEEEEEEE	-	Yes	Yes
Polyalanine (Control)	AAAAAAAAAAAA	-	Yes	No
LK-12 (Control)	LKLKLKLKLKLK	-	Yes	No
Random 1	YIIPDMKKAYPC	–20.6	Yes	No
Random 2	GYLCFKHPGIIT	–21.1	Yes	No
Random 3	KNFGDDRDTNTI	–3.7	Yes	No
Random 4	IGCPSTWEEMHV	–15.6	Yes	No
Random 5	WTYRMTASMRNF	–15.1	Yes	Yes

a Uncertainty calculated from the average change in ΔG_ads_ of 12 peptides upon repeating simulations.

b Peptide could not be synthesized.

### Evaluation of Ten *De Novo* Designed Polyethylene-Binding Peptides via Steered MD and SMFM

We evaluated the ten select peptides for polyethylene identified in the previous section by calculating ΔG_ads_ using SMD (Δ*G*_*ads*_^*SMD*^; Figure 2) and experimentally measuring adhesive pull-off forces (F_pull‑off_) using SMFM (Figure 3). Both SMD and SMFM quantify peptide adhesion to polyethylene by repeatedly desorbing a single peptide molecule (Figure 3B). As detailed in Methods, SMD calculates ΔG_ads_ using the Jarzynski relation.^60^ SMFM determines F_pull‑off_ by measuring the distribution of forces required to detach (desorb) a single peptide from a plastic surface over hundreds of repeat trials, fitting the pull-off forces to a Gaussian distribution, and assigning F_pull‑off_ to the mean of the Gaussian fit^28,31,63,64^ (a representative F_pull‑off_ histogram is shown in Figure 3C; see also Methods, SI Section 9, and the SI data file for details on data fitting and analysis). We note that rupture forces measured by SMFM are inherently non-equilibrium quantities with absolute magnitudes that depend on experimental parameters (e.g., rate of pulling; see SI Section 8, Table S3). Here we use the magnitudes of pull-off forces as a proxy for equilibrium binding free energies. This approach is guided by a number of prior studies that report mean rupture forces to correlate with equilibrium binding free energies under conditions where key experimental parameters are held constant^65^ (e.g., spring constant, rate of pulling; see SI Sections 8 and 9). Higher peptide affinity for polyethylene corresponds to a more negative Δ*G*_*ads*_^*SMD*^ and a more positive F_pull‑off_. To provide control peptides against which to reference the *de novo* SBP designs, two types of controls were tested (Table 1). The first type of control was a random amino acid sequence where amino acid residues at each position were picked independently and with equal probability. This random sequence allowed us to determine if the *de novo* design methodologies outperformed random chance. The second type of control used sequences of a single type of amino acid. In particular, we predicted that polyglutamic acid would provide a likely lower limit on peptide affinity, since its large net negative charge and hydrophilic properties were predicted to generate low affinity for a nonpolar plastic surface. In addition, we used polyalanine and a 12 amino acid residue sequence comprised of leucine and lysine (LK-12) to allow us to compare the designed peptides to non-polar or amphiphilic peptides. These control peptides were selected as initial benchmarks because there are no experimentally validated peptide sequences for polyethylene-binding against which we could compare our measurements (see SI Section 1 for discussion). This report establishes a baseline that can be used for comparison in future studies.

SMD and SMFM results in Table 2 reveal that the designed peptides have high affinities for polyethylene. The values of Δ*G*_*ads*_^*SMD*^ of 7 of 10 designed SBPs are more favorable than Δ*G*_*ads*_^*SMD*^ for the control peptides­(Figure 4A and Table 2). The differences are statistically significant: a one-sided *t* test comparing the average Δ*G*_*ads*_^*SMD*^ of the designed peptides to the random peptides gives a p-value of 0.017. A 95% confidence interval for the average Δ*G*_*ads*_^*SMD*^ of the designed peptides is – 26.9 ± 3.6 kcal/mol whereas a 95% confidence interval for the average of the random peptides is −17.7 ± 5.9 kcal/mol. Furthermore, the SBP with the highest predicted affinity for polyethylene is MCTS 1 (Δ*G*_*ads*_^*SMD*^: −37.5 ± 6.4 kcal/mol), which greatly outperforms all controls and most other designed SBPs (Table 2). SMFM shows that 6 of 10 designed peptides have higher average F_pull‑off_ than the best performing random peptide based on Δ*G*_*ads*_^*SMD*^, and these differences are statistically significant for MCTS 1 (p-value of 0.049) and PepBD 3 (p-value 0.037, the SI data file). In addition, the experimentally measured pull-off forces shown in Figure 3 are large, including when compared to the unfolding forces of mechanically stable proteins and protein–protein interactions^46–49,66^ (e.g., 150–300 pN for mechanically stable I-type immunoglobulin modules from human cardiac titin,^67,68^ or 85–200 pN for Lys48-C-linked or N–C-linked polyubiquitin chains, respectively^69^). Figure 4B and Table 2 show that SMFM results broadly agree with SMD predictions (Figure 4B and Table 2). Namely, most of the designed SBPs outperform the controls, and MCTS 1 exhibits the strongest adhesion (pull-off forces) for polyethylene (F_pull‑off_: 659 ± 68 pN). The magnitude of F_pull‑off_ for MCTS 1 is larger than previously reported SBPs of similar length (e.g., 220–610 pN^32^ for silica binding peptides and <200 pN for plasma fibronectin domains interacting with multiple solid surfaces^70^).

**Figure 4:**
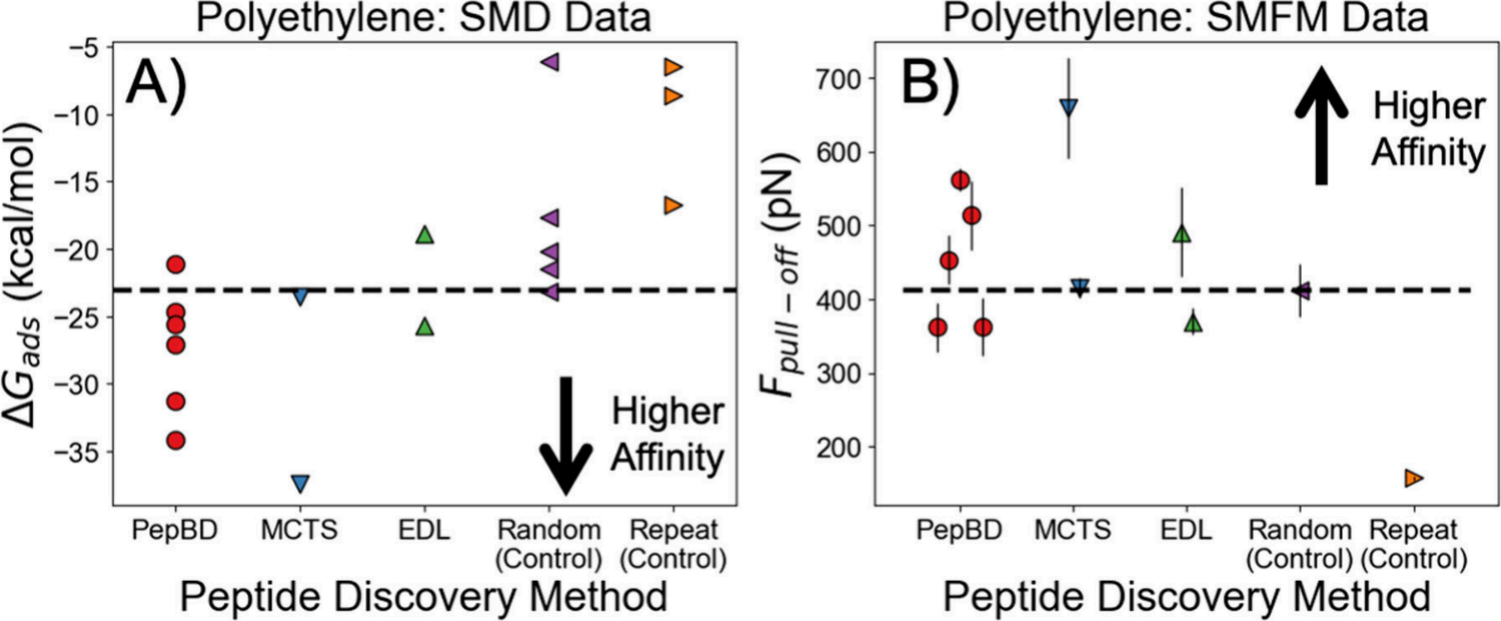
Simulations and experiments show that the computationally designed peptides adsorb strongly to polyethylene. A) ΔG_ads_ for peptides calculated with SMD simulations, or Δ*G*_*ads*_^*SMD*^. B) F_pull‑off_ for peptides measured using experimental SMFM. In both plots, each data point corresponds to one peptide sequence shown in Table 1, and dashed lines indicate the random peptide with the highest affinity. High affinity (or, strong adhesion) is associated with a more negative Δ*G*_*ads*_^*SMD*^ (more positive F_pull‑off_). Affinity data for each peptide are given in Table 2. Data for individual peptides are provided in the SI data file: “SMFM & MD Data” sheet. Error bars for ΔG_ads_ are taken from the maximum deviation in Table S2. Error bars for F_pull‑off_ are 1 standard error of mean (SEM) from 3 replicate measurements.

**Table 2:** Average and Best ΔG_ads_ and F_pull‑off_ Values for *De Novo* Designed Peptides Binding to Polyethylene^a^

Peptide Category	Δ*G*_*ads*_^*SMD*^ (±6.4 kcal/mol), Average/Best	F_pull‑off_ (pN), Average/Best
PepBD	–27.3/–34.2	451 ± 40/562 ± 15
MCTS	**–30.5/–37.5**	**537 ± 122/659 ± 69**
EDL	–22.3/–27.8	431 ± 32/491 ± 61
Controls	–10.6/–15.7	152 ± 6
Random	–17.7/–23.1	412 ± 36

a Bold entries indicate peptides that exhibit the strongest adsorption to polyethylene based on SMFM or SMD. The F_pull‑off_ error values indicate standard error of the mean (SEM).

We found SMFM and SMD measurements of peptide affinity for polyethylene to be strongly correlated: the Pearson correlation coefficient for Δ*G*_*ads*_^*SMD*^ and F_pull‑off_ is 0.93 (Figure 5A). The strong correlation between simulation and experiment is significant given the simplifications underlying SMD. Our simulations used crystalline polyethylene and amorphous polypropylene models similar to previous computational studies.^200,201^ The model surfaces used in simulations are idealizations of the experimental surfaces, with experimental polyethylene samples being typically semicrystalline, involving imperfections and heterogeneities (e.g., from branching and the presence of plasticizers^71^). Additionally, accurate free energy calculations using SMD require sampling of low probability events,^72,73^ which here correspond to peptides following a low energy pathway during desorption from polyethylene. The strong correlation between SMD and SMFM results suggests that SMD predicts relative peptide affinity with a useful accuracy despite these limitations, thus providing a viable approach to computational screening of future designs of SBPs without incurring experimental costs.

**Figure 5:**
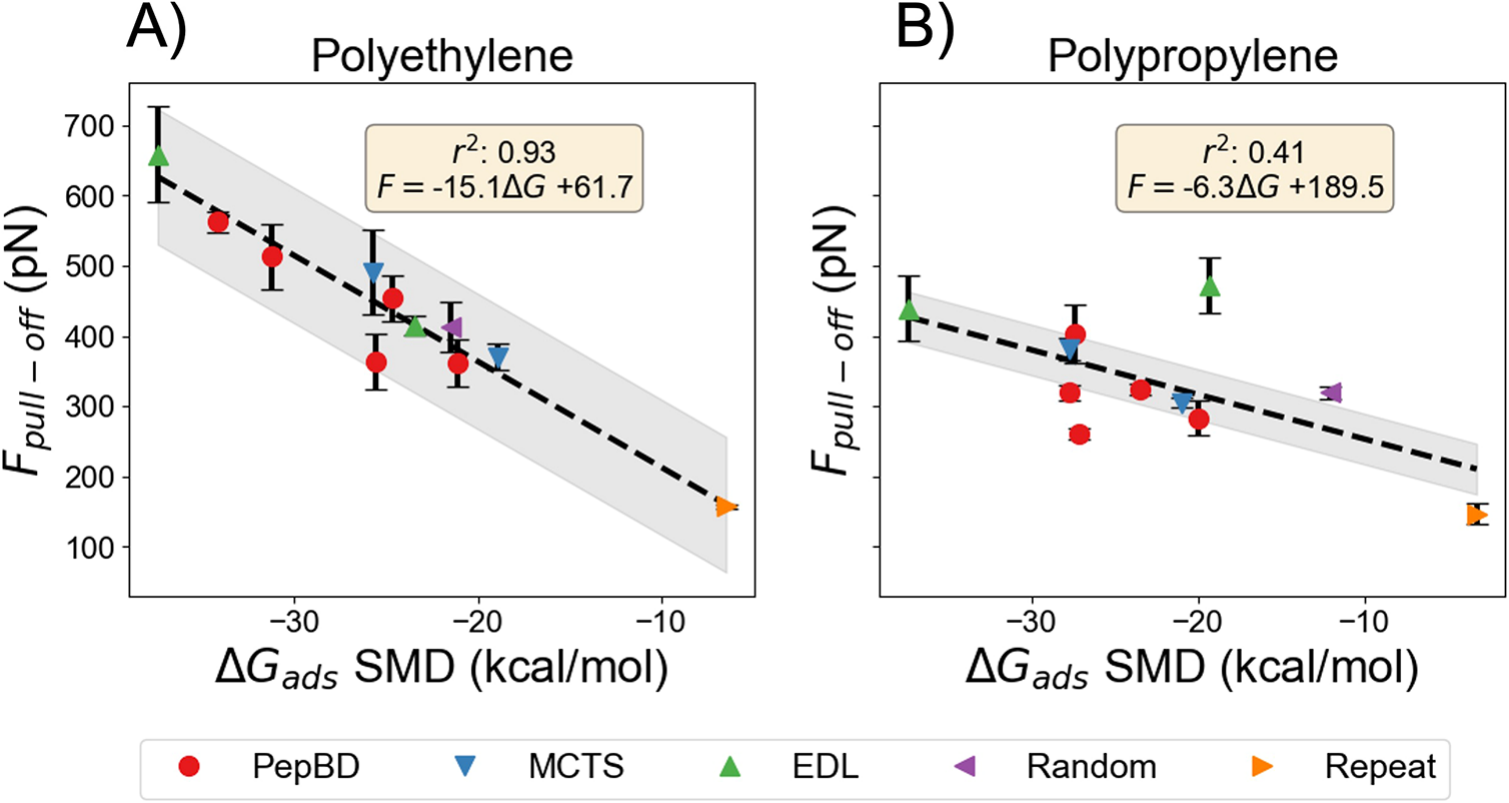
Computational SMD predictions correlate with experimental SMFM measurements. Correlations between F_pull‑off_ versus Δ*G*_*ads*_^*SMD*^ are plotted for A) polyethylene and B) polypropylene. The amino acid sequence in each *de novo* designed peptide is provided in Table 1. R-squared values were calculated using a Pearson least-squares regression fit. Error bars for F_pull‑off_ are 1 standard error of mean (SEM) from 3 replicate measurements. The shaded region shows uncertainty in ΔG_ads_ values taken from the maximum deviation in Table S2. Note that the *x*- and *y*-axis limits are identical in the two plots.

### Polyethylene-Binding Peptides Also Adsorb Strongly to Polypropylene

While the SBPs evaluated above were optimized for binding to polyethylene, we predicted that the SBPs may also adsorb strongly to polypropylene since both plastics are polyolefins. Evaluation of the peptides in Table 1 by repeating SMFM and SMD testing on polypropylene largely confirms this prediction (Figure S9, Table S6). We find that the designed SBPs tend to have greater affinity for polypropylene as compared to control peptides, but not all SBPs outperform the random control. Comparison of F_pull‑off_ values between polyethylene and polypropylene shows a modest correlation, with a Pearson correlation coefficient of r^2^ = 0.55 (Figure 6A). Thus, strong peptide adsorption to polyethylene is typically associated with strong adsorption to polypropylene, and vice versa. A byproduct of this correlation is that MCTS 1, the best SBP for polyethylene, has the second strongest adhesion for polypropylene out of all tested peptides. This suggests that MCTS 1 is a promising SBP for further evaluation in the context of addressing polyethylene and polypropylene MNP pollution.

**Figure 6:**
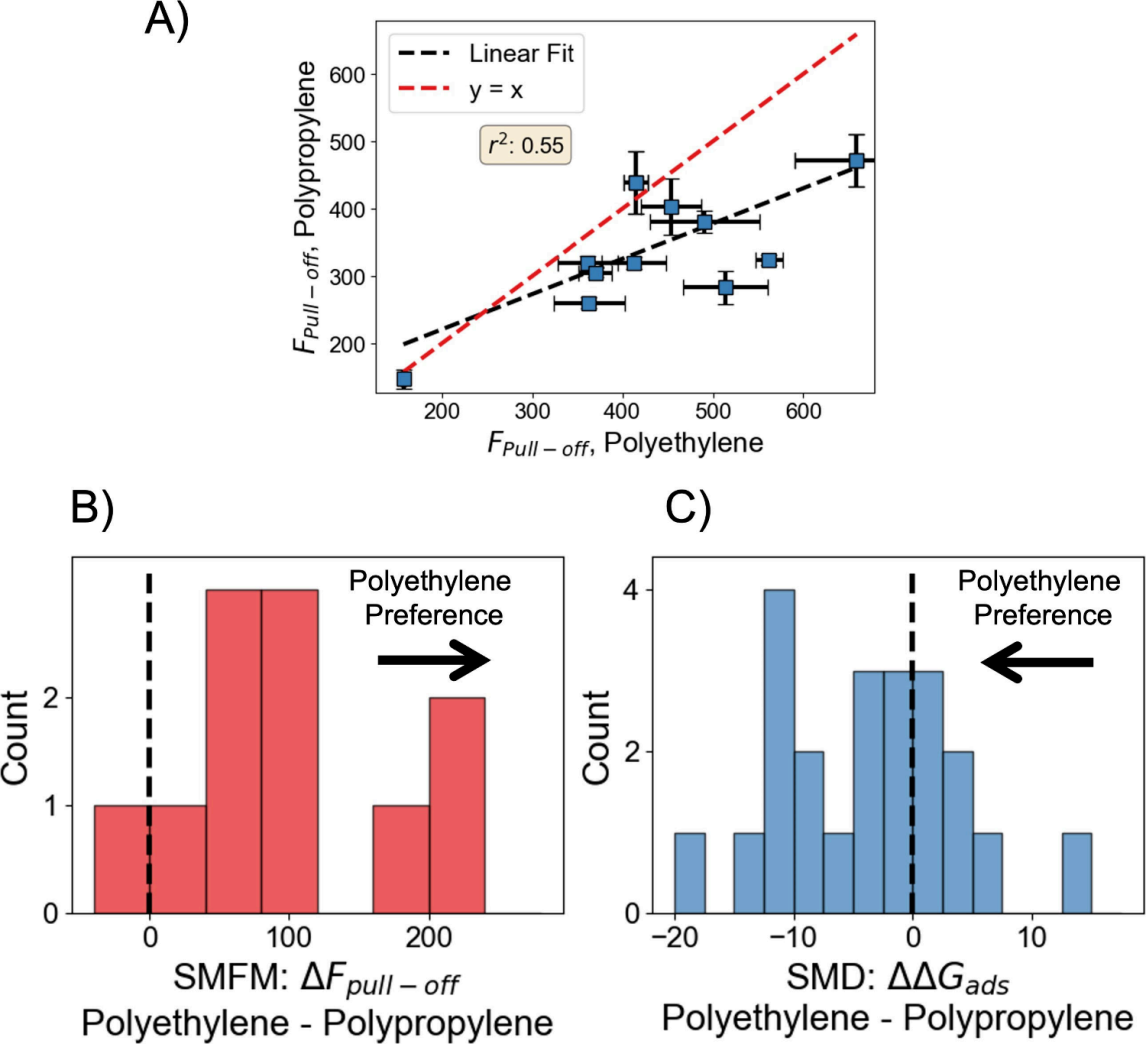
Relative strengths of adhesion of the designed peptides are correlated onpolypropylene andpolyethylene surfaces, but the peptides tend to adsorb more strongly to polyethylene. A) Correlation between F_pull‑off_ for polypropylene and polyethylene for the *de novo* designed peptides shown in Table 1. The r-squared Pearson correlation coefficient corresponding to the black dashed curve is provided. The red dashed curve shows the y = x line. B) Difference in F_pull‑off_ between polyethylene and polypropylene (Δ*F*_pull‑off_) for the peptides in A). C) Difference in ΔG_ads_ between polyethylene and polypropylene (ΔΔ*G*_*ads*_^*SMD*^) for the peptides in A). C) also includes 4 SBPs for polypropylene (Table S7). Arrows in B) and C) point in direction of increasing specificity for polyethylene.

The use of SMD for the validation of SBPs for polypropylene appears to be less accurate than for polyethylene. We make this interpretation based on the weaker correlation between Δ*G*_*ads*_^*SMD*^ and F_pull‑off_ for polypropylene (r^2^ = 0.41, Figure 5B) than for polyethylene (r^2^ = 0.93, Figure 5A). In general, we find that the range of F_pull‑off_ values is smaller for polypropylene than for polyethylene, even though the range of Δ*G*_*ads*_^*SMD*^ values predicted by SMDs is roughly the same for the two plastics. The larger errors in SMD predictions for polypropylene (versus polyethylene) increase the likelihood that a peptide with high affinity for polypropylene will not be selected by SMD screening for experimental validation. This is exemplified by MCTS 2: SMD predicted that the peptide has relatively weak affinity for polypropylene, yet SMFM found it to have the strongest adhesion out of all peptides tested. The weaker agreement between simulations and experiment likely arises from the greater challenge of computational sampling of peptide desorption from polypropylene than polyethylene. We expand on this topic in the Discussion.

### The Designed SBPs Tend to Prefer Polyethylene over Polypropylene, but the Specificity Varies Greatly between Peptides

Three observations regarding the SMD and SMFM results shown in Figure 6 reveal that the designed SBPs generally adsorb more strongly to polyethylene than to polypropylene (see also Table 1). First, nearly all of the data points in Figure 6A lie below the *y* = *x* line. Second, six peptides characterized by SMFM have statistically significant differences in F_pull‑off_ between the two plastics (SI data file). Third, the average per-peptide differences in F_pull‑off_ and Δ*G*_*ads*_^*SMD*^ between polyethylene and polypropylene are 100 pN and −3.9 kcal/mol, respectively (Figure 6B, C), where both values correspond to stronger adhesion to polyethylene. These differences are statistically significant, as a two-sided paired *t* test gives p-values of 0.011 for differences in Δ*G*_*ads*_^*SMD*^ and 0.00098 for differences in F_pull‑off_. Since all the SBPs described above were designed with polyethylene as the target, we also evaluated four SBPs designed by PepBD for polypropylene^14^ to determine if a preference for polyethylene was still observed. The peptides were only evaluated with SMD to avoid experimental cost. Interestingly, the peptides also exhibited greater affinity for polyethylene than for polypropylene (Table S7). Even when only considering the random peptides and the SBPs found by PepBD for polypropylene, we still find a statistically significant difference in Δ*G*_*ads*_^*SMD*^ between polyethylene and polypropylene (ΔΔ*G*_*ads*_^*SMD*^: −6.2 kcal/mol; two-sided paired *t* test p-value: 0.013). We also performed simulations using umbrella sampling^74^ (SI Section 17) to evaluate the relative tendency of the designed peptides in Table 1 to bind to polyethylene versus polypropylene; these additional simulations revealed that all but one peptide have greater affinity for polyethylene than polypropylene. The general preference of the peptides for polyethylene over polypropylene likely reflects differences in crystallinity in the polymer surface models used in our SMD simulations (Figure S10)- a fully crystalline polyethylene and an amorphous polypropylene surface (see Figure S11 and S12 for corresponding characterization of polyethylene and polypropylene surfaces used in our experiments). Prior studies have shown that greater crystallinity in a non-polar surface can increase the magnitude of hydrophobic forces.^75^ Below we return to the role of hydrophobic forces in peptide affinity.

The preference of the SBPs for polyethylene versus polypropylene, however, varies significantly between the peptides (as measured by distance from the *y* = *x* line in Figure 6A). Nearly all peptides lie below the line, indicating greater preference for polyethylene, as just discussed. However, some points deviate farther from the *y* = *x* line than others, corresponding to greater preference for polyethylene. The deviation increases as the strength of peptide adhesion to polyethylene increases, since the best-fit line has a slope smaller than 1. However, one peptide lies just above the *y* = *x* line, suggesting a small preference for polypropylene, although the difference lies within the range of experimental uncertainty (see below for additional comments under a discussion of future directions). Table 3 lists example peptides with different degrees of preference between polyethylene and polypropylene. Encouragingly, SMFM and SMD agree on assessments of peptide specificity.

**Table 3:** Designed Peptides that Display Different Preferences between Polyethylene and Polypropylene Surfaces

Description	Peptide	ΔΔ*G*_*ads*_^*SMD*^(±12.1 kcal/mol), Polyethylene – Polypropylene	ΔF_pull‑off_ (pN), Polyethylene – Polypropylene
Preference for Polyethylene	MCTS 1	–18.1	187 ± 4
	PepBD 3	–10.7	239 ± 4
No Preference	EDL 2	2.1	65 ± 1
	PepBD 2	1.4	50 ± 2
Preference for Polypropylene	MCTS 2	13.9	–24 ± 5

### SBP Affinity is Driven by Strong Hydrophobic Interactions

As polyethylene and polypropylene are both non-polar plastics, we expected that hydrophobic interactions would play an important role in peptide binding. The potential role of hydrophobic interactions was probed by replacing water (in SMD; Table S8) or aqueous PBS buffer (in SMFM) with 60% by volume methanol. This choice of mixed solvent was guided by the results of a series of prior experimental investigations^28,29,31^ revealing that the addition of 60 v% methanol disrupts hydrophobic interactions while having limited measurable impact on van der Waals and electrostatic interactions. We note that the refractive index of 60 v% methanol is identical to water, hinting at why van der Waals interactions are similar in water and the mixed solvent system. Also, the dielectric constant of 60 v% methanol in water is high (approximately 52 ^76,77^ ), which likely underlies the observation that electrostatic interactions are not substantially perturbed in the mixed solvent system. For example, Ma et. al,^28^ found that 60 v% methanol substantially reduced adhesion between two hydrophobic surfaces but did not measurably alter the magnitude of adhesive pull-off forces between surfaces with opposite charge, consistent with selective disruption of hydrophobic interactions. We term this mixed solvent system “60% methanol” for the remainder of this paper. Guided by the above-described prior studies, we attribute differences in the values of Δ*G*_*ads*_^*SMD*^ and F_pull‑off_ between water/PBS versus 60% methanol to indicate a role for hydrophobic interactions. Four representative peptides were selected for evaluation: MCTS 1 and 2, EDL 1, and PepBD 2. For each of these peptides, F_pull‑off_ and Δ*G*_*ads*_^*SMD*^ are reduced 40–80% when comparing measurements performed in water/PBS and in 60% methanol, for both polyethylene and polypropylene (Figure 7 and S13, SI data file). This result is consistent with hydrophobic interactions playing an important role. We note also that changes in hydrophobic interactions of the non-polar amino acids of the peptides with the polyolefin surfaces likely introduces changes in the distributions of conformations sampled by the peptides at the surfaces. Variability in the percent reduction of adhesion in 60% methanol between peptides suggests that hydrophobic forces are stronger for some peptides than others. The contribution of hydrophobic interactions was largest for MCTS 1 (F_pull‑off_ decreased by 78.5 ± 0.1% for polyethylene and 62.1 ± 0.4% for polypropylene) and MCTS 2 (F_pull‑off_ decreased by 47.2 ± 0.3% for polyethylene and 36.1 ± 0.8% for polypropylene). Although our SMD simulations and SMFM experiments clearly indicate that hydrophobic interactions play an important role, we found no direct correlation between the magnitude of hydrophobic interactions and peptide physicochemical properties commonly associated with hydrophobic interactions, namely peptide solubility predicted via the CamSol score^78^ or from peptide volume (Figure S14).

**Figure 7:**
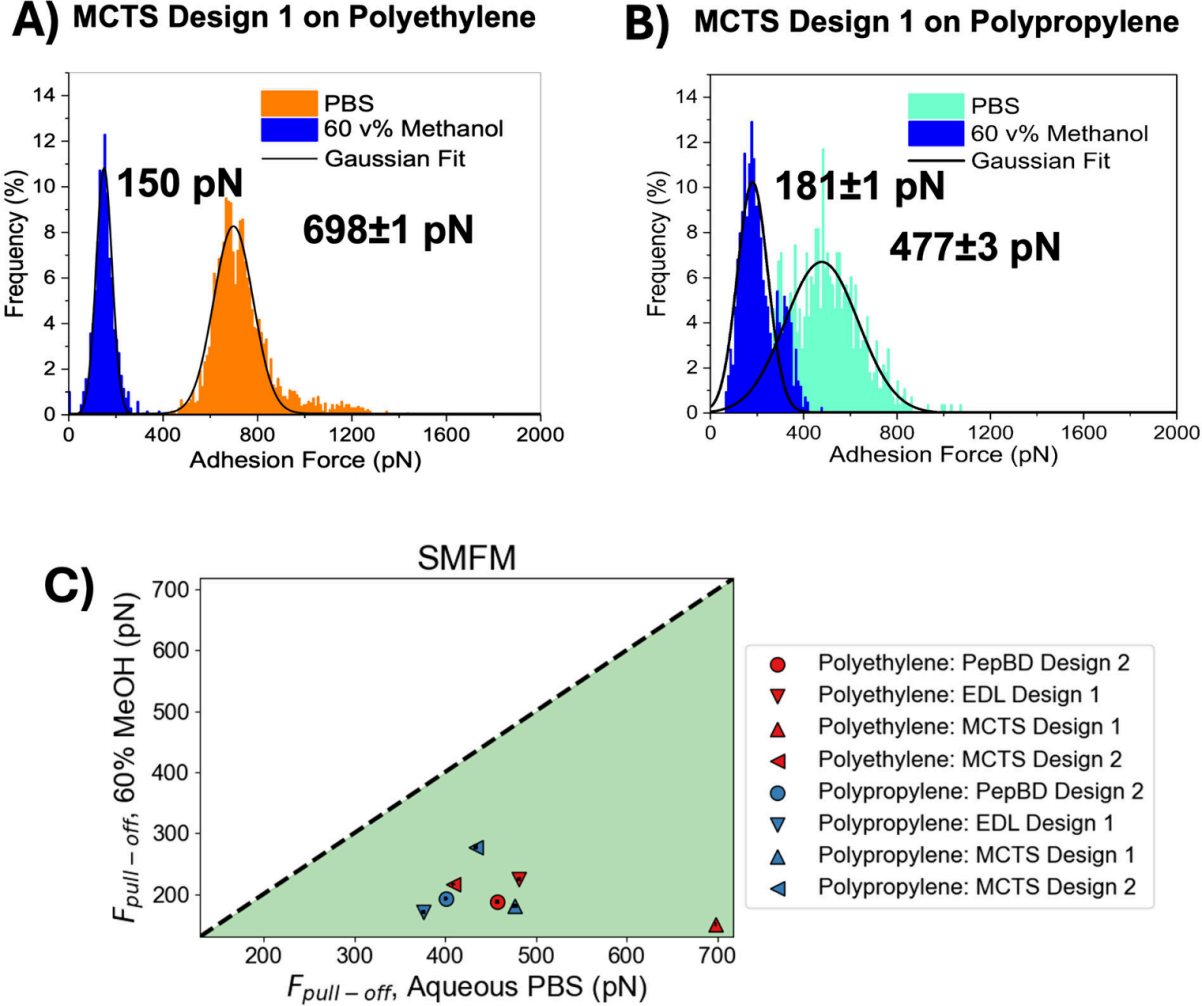
Hydrophobic interactions make a major contribution to the adhesion of the designed peptides to polyethylene and polypropylene. Each plot shows a histogram of F_pull‑off_ values from 1500 measurements of the peptide MCTS 1 with either A) polyethylene and B) polypropylene, in either aqueous PBS or 60% methanol in aqueous PBS. The mean and standard deviation obtained by fitting a Gaussian distribution to the experimental data are shown. C) The plot shows F_pull‑off_ ± SEM values in aqueous PBS and 60% methanol in PBS for 4 different *de novo* designed peptides binding to polyethylene (red markers) or polypropylene (blue markers). The dashed line shows *y* = *x* and corresponds to F_pull‑off_ being equal in PBS and 60% methanol added to PBS. All points in the green shaded region have stronger adhesion to the plastics in PBS than in 60% methanol added to PBS. Corresponding calculations of Δ*G*_*ads*_^*SMD*^ in both water or 60% methanol added to water showa similar trend and are provided in Table S8. Raw data are provided in the SI data file: “Aqueous PBS vs. Methanol” sheet.

### Additional Insights into the High Affinity of *De Novo* Designed SBPs

To provide additional insight into the SBP designs that exhibited the strongest affinities for polyethylene and polypropylene surfaces, we analyzed the equilibrated adsorbed conformations of all strong binding peptides obtained from SMD simulations and quantified the distribution of distances between the centers of mass of each amino acid and the polymer surfaces. Our analysis of amino acid contact patterns yielded six key insights, which we summarize here (see SI Section 18 for an expanded discussion):

i. The designed peptides adsorb to polyethylene and polypropylene surfaces by localizing non-polar aromatic and aliphatic residues near the polymer surfaces (<4–5 Å), while partitioning charged residues (particularly D, E, and K) away from the surfaces into solution (>7 Å) (Figure S15). These results provide additional support for our conclusion that peptide binding to both polyethylene and polypropylene surfaces is driven largely by hydrophobic interactions, as inferred from experiments and SMD simulations reported above.
ii. The contact patterns of amino acid residues in the designed peptides differ subtly between polyethylene and polypropylene: ionic residues K, D, and E, and polar residues S and T localize further from the polyethylene surface than from the polypropylene surface, while nonpolar residues such as I and L are more closely associated with the polyethylene surface than on the polypropylene surface (Figure S15). These differences in segregation of charged and non-polar amino acids are consistent with stronger hydrophobic interactions with polyethylene (Figure 6) and with prior reports that hydrophobic interactions are stronger at ordered aliphatic surfaces than at disordered ones (as noted above, the polyethylene used in our study is crystalline whereas polypropylene is amorphous).^79^
iii. Blockiness in the sequences of non-polar and polar residues, instead of specific binding motifs, characterize the structure of the designed peptides with high affinity for polyethylene. Inspection of Figure 8A reveals the characteristic blockiness in the sequence of non-polar and charged amino acid residues within both MCTS 1 and MCTS 2: MCTS 1 has two non-polar blocks (WFF and WWMM) while MCTS 2 has a single non-polar block at the C-terminus (WWMIFF). We hypothesize that this blockiness plays a key role in enabling the segregation of non-polar and charged amino acid residues in polyethylene-bound high affinity peptides, as discussed in point (ii) above, by allowing the blocks of non-polar amino acids to adsorb to the polyethylene surface and the blocks of charged residues to loop away from the surface. The other high affinity peptides identified in our study also exhibited similar blockiness (see sequences in Table 1), supporting our proposal that blockiness is a characteristic feature of the *de novo* designed high affinity peptides.
iv. The *de novo* designed peptides MCTS 1 and MCTS 2 both contain a tetrapeptide block of four non-polar amino acid residues (WWMM or WWMI, respectively; see Figure S16 and S17). Interestingly, WWMM of MCTS 1 localizes to a greater extent at the polyethylene surface than WWMI from MCTS 2, even though I is more hydrophobic than M. This difference is noteworthy also because WWMM of MCTS 1 is flanked on either side by two ionic amino acid residues (EK and RR) that extend away from the polyethylene surface to a greater extent than the flanking residues for MCTS 2 (DY and FF). Overall, this comparison of MCTS 1 and MCTS 2 binding to polyethylene supports our proposal that strong binding peptides (we measured MCTS 1 to bind to polyethylene more strongly than MCTS 2 (Table 3) effectively segregate non-polar and charged amino acids when adsorbed.
v. The same triad of non-polar amino acid residues (WWM) within MCTS 1 and MCTS 2 show differential preferences for binding to polyethylene versus polypropylene surfaces depending on sequence context. The non-polar triad WWM of MCTS 1 localizes more closely to the surface of polyethylene than polypropylene, but WWM of MCTS 2 localizes closer to polypropylene than polyethylene (highlighted in Figure S16B). This result illustrates how the relative extents of association of a specific non-polar peptide block within a sequence depends on both the sequence context and the polymer surface properties (the latter reflecting differences in the structure of polyethylene and polypropylene surfaces, as discussed above).
vi. All *de novo* designed peptides exhibit diverse adsorbed conformations on polyolefin surfaces, with the ensemble of adsorbed conformations differing between polyethylene and polypropylene. Past studies have hypothesized that a key challenge underlying the design of SBPs, which is distinct from peptides binding to protein pockets, is that SBPs typically adopt a broad range of conformations when adsorbed to surfaces of solids.^20^ ,^21^ Our results support this hypothesis, as evidenced by the broad surface-distance distributions, often with multiple peaks, particularly for the charged residues (Figure S15).

**Figure 8:**
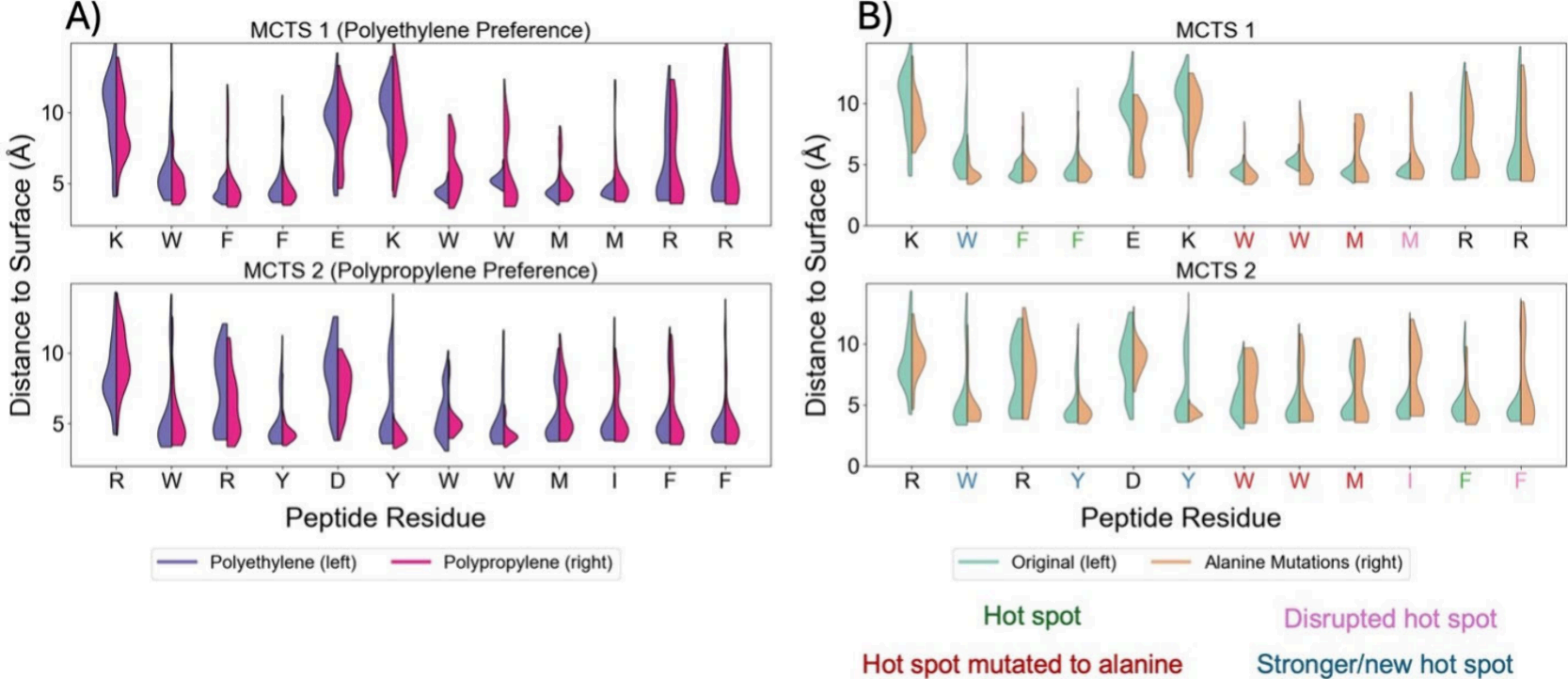
Contact patterns of MCTS 1 and 2 on polyolefin surfaces and how they are changed by alanine mutations. (A) Binding of MCTS 1 and MCTS 2 to polyethylene and polypropylene. Each panel shows the distribution of distances between each amino acid side chain center of mass and the plastic surface for equilibrated adsorbed conformations obtained in SMD simulations. Comparison of adsorbed conformations for MCTS 1 (top) and MCTS 2 (bottom) on polyethylene (purple) and polypropylene (pink). B) Identifying hot spot residues in MCTS 1 and 2 based on the adsorbed conformations of the peptides before and after alanine mutations. Comparison of adsorbed conformations for MCTS 1 (top) and MCTS 2 (bottom) before (green) and after (orange) alanine mutations. Amino acid letter colors indicate hot spots (green), residues mutated to alanine (red), hot spots disrupted after alanine mutations (pink), and new or strengthened hot spots after alanine mutations (blue). Sequences and Δ*G*_*ads*_^*SMD*^ values for the peptides are provided in Table 4.

### The Role of Hot Spot Residues in the *De Novo* Designed MCTS Peptides

Observations (iv) and (v) above reveal that the triad WWM of MCTS 1 and MCTS 2 associate differently with polyethylene, and exhibit differential preferences for polyethylene versus polypropylene. These observations motivated us to investigate further how residues strongly associating with the polymer surfaces contribute to peptide affinity. Strongly associating residues are commonly called ″hot spots,″ and have been reported for peptides that bind to other solid materials.^8,80,81^ The most common approach used to find a hot spot in a polypeptide is alanine scanning:^82^ mutate one residue to alanine, measure the change in affinity, and repeat for all residues. The cost of SMD and SMFM makes this exhaustive approach impractical. We instead identify hot spots as residue sequences (i.e., motifs) that consistently contact the polymer surface in their equilibrated adsorbed conformations in SMD simulations (similar to analysis above) and evaluate the impact of mutating these hot spots simultaneously to alanine (SI Section 19 and Figure S18). Accordingly, we mutated WWM to alanine in MCTS 1 and MCTS 2 (color coded in red in Figure 8B), and then repeated SMD simulations.

By comparing the amino acid contact patterns before and after mutation of the peptides (Figure 8B), we identify new hot spots (i.e., hot spots not present in the original peptide), strengthened hot spots (i.e., hot spots present in the original peptide but found to contact the polymer more consistently after mutation), and disrupted hot spots (i.e., hot spots present in the original peptide but absent in the mutated peptide) (color coded in Figure 8B). For MCTS 1, the mutations reduced Δ*G*_*ads*_^*SMD*^ by ∼33% (Table 4), but no significant changes in the peptide’s adsorbed conformations were observed (Figure 8B, top row, orange) other than the expected disruption of hot spots mutated to alanine (given the analysis of polyalanine in Figure S18). The other hot spot motif in MCTS 1, WFF, was unaffected by alanine mutations and no additional hot spots were observed to form, resulting in an overall decrease in affinity of the mutated peptide for polyethylene (to Δ*G*_*ads*_^*SMD*^ of −24.4 kcal/mol; i.e., 33% decrease). For MCTS 2, the mutations did not impact ΔG_ads_ but changed the adsorbed conformations substantially (Figure 8B, bottom row, orange). Hot spots both in the mutated WWM and the adjacent IFF motif were disrupted, while one tryptophan (W) and two tyrosine (Y) residues at the N-terminus became new hot spots. These compensatory changes likely explain why the overall Δ*G*_*ads*_^*SMD*^ did not change, and indicate that a residue can be a hot spot for one peptide but not another. Thus, consistent with the physical picture described above in points (iv) and (v) of the previous section, the hot spot analysis supports the idea that accurate prediction of peptide affinity for plastic (or other materials) cannot be achieved by treatment of amino acids individually (or, sequence specific motifs), but requires consideration of the peptide sequence as a whole.

**Table 4:** Influence of Alanine Mutations on *De Novo* Designed Peptide Affinity for Polyethylene (Determined via SMD Simulations)

Peptide	Sequence^a^	Δ*G*_*ads*_^*SMD*^ (±6.4 kcal/mol)	ΔΔ*G*_*ads*_^*SMD*^ (±12.8 kcal/mol) Original – AlaScan
MCTS 1, Original	KWFFEK**WWM**MRR	–37.5	+13.1
MCTS 1, AlaScan	KWFFEK **AAA** MRR	–24.4	
MCTS 2, Original	RWRYDY**WWM**IFF	–23.5	–2.9
MCTS 2, AlaScan	RWRYDY **AAA** IFF	–26.4	

a Bold underlines indicate where residues were mutated to alanine.

### MCTS 1 Assumes Diverse Adsorbed Conformations on Polyethylene and they Differ Substantially from those on Polypropylene

As discussed above in the context of observation (vi), analysis of the contact patterns of the *de novo* designed peptides with polyethylene and polypropylene provides support for the proposal that SBPs are challenging to design because the peptides typically adopt a wide range of conformations when bound at solid surfaces. Here we provide additional support for this proposal in the context of MCTS 1 by evaluating and comparing the diversity of conformational states assumed by the peptide at polyethylene and polypropylene surfaces. We approach this goal by calculating the free energy surface of bound MCTS 1 using parallel-bias well-tempered metadynamics (PBMetaD, SI Section 20).^83^ The free energy surfaces provided by PBMetaD were considered to have converged when their fluctuations were not significantly greater than 1.2 kcal/mol (thermal energy at 300 K) by the end of the simulation (Figures S19 and S20). The 2-D free energy surface as a function of the peptide radius of gyration (R_gyr_) and the distance between the center of mass of the peptide and the polyethylene surface (z_pep_) (Figure 9A) shows that all R_gyr_ values have equal free energy values when the peptide is far from the polyethylene surface. In other words, the peptide is unstructured in solution. Inspection of Figure 9A reveals that as the peptide approaches the polyethylene surface, an extended conformation becomes increasingly stable (Figure S21). The peptide is stably adsorbed when z_pep_ is 0.6 to 0.8 nm and R_gyr_ is between 0.80 and 0.95 nm (Figure S22 and S23). Based on these values of R_gyr_, we conclude that the adsorbed peptide adopts a structure somewhere between a compact globule and the fully extended state. Examples of such structures are shown in Figure 9B. A key implication of Figure 9A and 9B is that the peptide does not have a single stable adsorbed conformation, but rather many stable adsorbed conformations.^20,21^ This is distinct from peptides binding to protein pockets,^84–86^ where peptides are typically constrained by interactions with the protein to a limited range of conformations. Furthermore, we also find that MCTS 1 assumes substantially different ensembles of adsorbed conformations on polyethylene and polypropylene (Figure S24), with MCTS 1 tending to assume conformations that extend along the surface (larger R_gyr_) when adsorbed to polyethylene as compared to polypropylene. ΔG_ads_ was calculated^87^ from the free energy as a function of z_pep_ (Figure S22) to give Δ*G*_*ads*_^*PBMetaD*^=-15.3 kcal/mol at a standard concentration of 1 mol/liter. This value of Δ*G*_*ads*_^*PBMetaD*^ indicates very strong binding when compared to the values previously reported for SBPs for a range of various materials,^5,88–90^ which typically lie in the range of −6 to −20 kcal/mol (see SI Section 7 for a discussion of ΔG_ads_ values calculated using different simulations methods).

**Figure 9:**
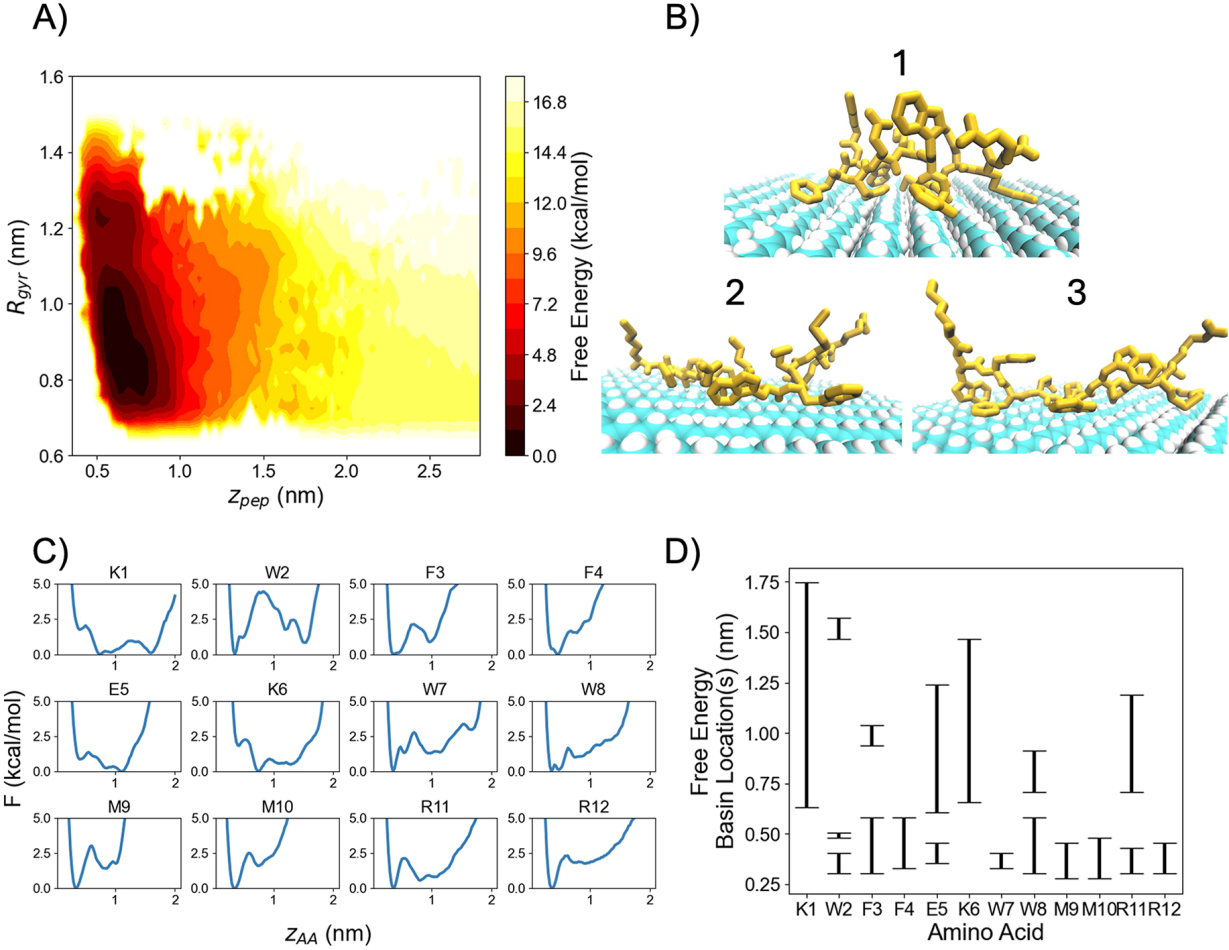
Free energy surface and stable bound conformations of MCTS 1 adsorbing to polyethylene. A) Two-dimensional free energy surface as a function of z_pep_ (the distance between the peptide center of mass and the top of the polyethylene surface) and R_gyr_ (the peptide’s radius of gyration). B) Examples of adsorbed conformations in the free energy minima indicated in panel A. C) One-dimensional free energy profile as a function of z_AA_ (the distance between the side chain center of mass and the top of the polyethylene surface) for each amino acid. D) Free energy basin for each amino acid, i.e. the range of z_AA_ values where F is within 1.2 kcal/mol of the minimum F value. Panel B made with VMD.^114^

The role of each amino acid in peptide adsorption was determined by obtaining free energy profiles as a function of the distance of each amino acid side chain from the polyethylene surface, or z_AA_ (Figure 9C). Several of the free energy profiles have minima at 0.3 and/or 1 nm, indicating multiple stable conformations for the amino acids. We highlight the minima by plotting the location of the free energy basin for each amino acid in Figure 9D, where we define the free energy basin as the range of distances over which the free energy is within 1.2 kcal/mol of the global minimum (1.2 kcal/mol is twice the thermal energy at 300 K). Lysine (K) and glutamic acid (E), both hydrophilic and ionic residues, have broad basins that are distant from the polyethylene surface. These residues thus appear to mediate peptide interactions with water. Conversely, arginine (R) has a basin that is narrow and proximal to polyethylene, even though it also is ionic. It thus appears that arginine helps the peptide interact with polyethylene, either because its side chain has some hydrophobic nature^91^ or because it influences hydrophobic interactions of nearby non-polar residues with polyethylene. Hydrophobic residues like tryptophan (W) and methionine (M) tend to have narrow basins proximal to polyethylene, indicating their strong interaction with polyethylene. However, tryptophan at positions 7 and 8 (W7 and W8) are exceptions to this generalization, as they have basins that are broad as compared to the narrow basin of the tryptophan at position 2 (W2). The different free energy profiles for tryptophan residues again emphasizes that the role played by an amino acid in peptide adsorption is not an intrinsic amino acid property but depends on the entire peptide sequence.

## Discussion and Conclusion

This paper critically assesses SBPs that emerge from recently reported SBP design methodologies that use biophysical models and ML-based approaches (Figure 1). We report a framework for evaluating SBPs by using a combination of simulation and experiments, and we demonstrate the utility of the framework by validating the first set of *de novo* designed peptides with strong adhesion to polyethylene. Below we summarize four key conclusions that emerge from our critical evaluation of the SBPs and the methodologies used for their design. We also discuss the implications of our findings for future designs of SBPs:

First, our evaluation of the predictions of the biophysical and ML-based design methodologies relied on implementation of both MD simulations (SMD) and experimental methods (SMFM). An important finding of our study is that the computational and experimental approaches used to evaluate the designed SBPs lead to similar conclusions. Agreement between the two approaches provides confidence to our conclusions regarding the relative performance of the SBPs designed for polyethylene. Significantly, it also hints that use of *either* simulations or SMFMs may, in the future, be sufficient for evaluating computationally designed SBPs.

Second, one *de novo* designed peptide, MCTS 1, is validated by our simulations and experiments to adsorb particularly strongly to polyethylene (F_pull‑off_: 659 ± 68 pN, Δ*G*_*ads*_^*PBMetaD*^: −15.3 kcal/mol). By benchmarking against previously reported high-binding SBPs, which have pull-off forces^32^ of 220 to 610 pN and ΔG_ads_ ^5,88–90^ values ranging between −6 to −20 kcal/mol, we conclude that the SBP design methodologies evaluated in this paper are effective at discovering new peptide sequences that exhibit high binding to targeted solids.

Third, our study can be used to compare the relative performance of biophysical modeling and ML methodologies used for SBP design. Significantly, our assessment of the performance of the preferred SBPs emerging from the three design methodologies is that they all are strong-binding, with all 9 of the designed sequences tested exhibiting pull-off forces between 361 ± 33 and 659 ± 68 pN on polyethylene surfaces. While the binding strengths are all strong, the peptide sequences identified by each methodology differ substantially, a conclusion we support with the Hamming distance similarity matrix shown in Figure S25. This metric, which quantifies the amino acid matches at each position, reveals a similarity of less than 19% across the 9 sequences. Thus, the ML design methodologies not only yield strongly binding peptides, but also expand the diversity of SBPs. This finding is significant, as ML methods can readily optimize other peptide properties (e.g., solubility) while maintaining strong binding to a targeted solid surface.

Fourth, a significant merit of our methodology for evaluation of SBPs is that it yields physical insights which, in turn, can be used to guide future biophysical and ML-based designs of SBPs. We find that the adsorbed states of the *de novo* designed peptides on polyolefin surfaces are characterized by broad free energy minima and multiple conformations. We conclude that strong binding arises from peptide sequences that adopt adsorbed conformations with statistical segregation of charged and nonpolar amino acids. Specifically, our results support a generalizable design principle for polyolefin-binding peptides that feature interspersed non-polar and charged blocks, where a range of aromatic and aliphatic residues form non-polar blocks that closely contact the polymer surface while blocks of charged residues localize away from the surface to maximize solvation. Interestingly, for the strongest binding peptides validated in our study (MCTS 1 and MCTS 2), we found that the peptides were comprised of exclusively nonpolar and charged amino acids (polar amino acid residues were not incorporated into the sequence). Overall, the physical picture that emerges from our study of SBPs stands in contrast to typical protein–peptide complexes, which often feature a dominant binding conformation stabilized by a well-defined binding pocket.^84–86^ Alanine mutation studies (hot spot analyses) performed using SMD simulations provide further support for the physical picture of SBPs described above by revealing that no single motif (no specific sequence of amino acids) functions as a universal hot spot for the *de novo* designed SBPs validated in our study.

While the SBPs validated in this paper show high affinity for polyolefins, several approximations need to be considered when applying the SBP design framework (Figure 1, top row) to other materials. In particular, PepBD makes two simplifications when calculating peptide affinity scores: 1) it does not calculate the loss in peptide conformational entropy upon adsorption to the solid surface, and 2) it uses an implicit solvent model to describe solvent effects. Both factors can be important in peptide adsorption, since the entropic driving force for peptide adsorption may differ greatly between peptides,^21,92^ and the structure of the interfacial solvent may play an important role in peptide adsorption.^81,93^ These two factors are omitted in PepBD because they are computationally intensive to evaluate. Due to this approximation, both PepBD and the ML models (which are trained on PepBD data) will tend to identify peptides whose adsorption is driven by enthalpy; we expand on the potential impact of these simplifying assumptions in SI Section 2. While these assumptions did not prevent the *de novo* design of peptides with high affinity for polyethylene, future studies are needed to assess the impact of these approximations on SBPs designed for other materials. We note that bias introduced by PepBD could be mitigated by pairing SMD data with Bayesian optimization,^94^ as has been used to improve peptide selectivity for gold or silver.^5^

The MD procedure used to evaluate SBPs may also require refinement when evaluating peptide affinities for other materials. SMD and SMFM measurements correlated more weakly for polypropylene than for polyethylene (Figure 5), which may reflect the limitations of SMD when used to calculate free energy differences. Our simulations use a fully crystalline surface for polyethylene and an amorphous surface for polypropylene (Figure S10). A peptide likely adopts a broader range of adsorbed conformations on an amorphous surface as compared to a crystalline surface. As SMD must thoroughly sample desorption pathways (especially pathways that occur with exponentially low probability^59^) to accurately determine free energies, calculating ΔG_ads_ poses a tougher sampling problem for polypropylene than polyethylene. The sampling problem becomes more challenging as the variation in the calculated work of desorption between simulations is larger than the thermal energy (i.e., kT),^95^ as observed here. We chose SMD despite these limitations because it had an acceptable computational cost (<10% of the computational time required by metadynamics), our primary use of Δ*G*_*ads*_^*SMD*^ values was to rank peptides, and SMD was found to provide consistent Δ*G*_*ads*_^*SMD*^ (see SI Section 6) that aligned with SMFM. We also evaluated peptides using umbrella sampling^74^ (SI Section 17), and found no substantial improvement in the correlation between umbrella sampling and SMFM, suggesting that the challenge of predicting peptide affinity for polypropylene is not unique to SMD. The results of umbrella sampling and SMD results also correlated for both plastics (Figure S26). Another simulation option for evaluating peptides is a high-throughput metadynamics method,^88^ although it requires approximately 10-fold longer simulation times than our SMD or umbrella sampling procedures.

Experimental methods other than SMFM can likely be used to measure peptide affinity for surfaces and may in fact be preferable for different materials. Although we used SMFM to evaluate peptide adhesion to plastics, other methods are possible for other materials, such as surface plasmon resonance for metal surfaces^96^, quartz crystal microbalance^97^ or fluorescence-based assays.^98,99^ These alternative methods have the appealing feature of directly providing kinetic and/or thermodynamic information regarding peptide adsorption. In contrast, SMFM provides only non-equilibrium force measurements, although SMFM data can be analyzed to estimate ΔG^‡^.^73^ We analyzed dynamic force spectroscopy measurement for MCTS 1 on polyethylene (Figure S27, SI Section 21) to estimate the kinetic parameters: barrier height, *x*_β_, dissociation rate, *k*_0_ and the activation energy for detachment ΔG^‡^. The ΔG^‡^ extracted from the dynamic force spectroscopy is 18.5 ± 0.6 kcal/mol, which is comparable in magnitude to the ΔG_ads_ obtained from metadynamics simulations (−15.3 kcal/mol).

### Key Insights and Future Directions

Our findings suggest several new directions of research at the intersection of *de novo* SBP discovery and material science. While our primary goal was to critically evaluate methodologies that use biophysical models and ML for the discovery of plastic-binding peptides, our results have broader implications that span both fundamental SBP design principles and technological potential.

First, our results suggest that a productive avenue for future research is understanding why specific peptides display differential binding affinities across target surfaces. The emerging understanding of the molecular basis of hydrophobic interactions mediated by proteins^86^ could potentially be employed in SBP design strategies. Additionally, our results indicate that *de novo* designed peptides for polyolefins achieve strong binding by segregating non-polar amino acids to the plastic surface and charged amino acids away from the surface into solution, and that this segregation is enabled by a characteristic blockiness of the designed sequences. Future studies could further test this proposal by characterizing sequence isomers with designed patterns of blockiness.

Second, our results suggest that the incorporation of ML methods in SBP design methodologies open up opportunities for multiobjective peptide design. Specifically, as discussed above, a noteworthy finding of our study is that we validated multiple strong binding peptide sequences for a specific target. This finding provides the opportunity to optimize for additional functional properties while maintaining strong binding to the target surface. Support for this proposed direction of investigation can be found in the MCTS ML-based design methodology used to discover several peptide sequences evaluated in our study. In addition to optimizing for binding affinity to polyethylene, the ML model leading to the MCTS designs selected for peptides that exhibit solubility in water. Depending on the context of interest, additional functional properties that could be designed into strong binding peptide sequences might include (i) low toxicity, (ii) tendency to form supramolecular structures, including self-association to form gels or soft material scaffolds,^100–102^ (iii) resistance to enzymatic degradation,^103^ and (iv) affinity for multiple surfaces^104,105^ (e.g., inorganic and biological interfaces).

Third, the results of our study enable the development of peptides for use in a range of potential applications. In this work, we have focused on peptides that bind to polyethylene and polypropylene because of the potential utility of the peptides in the context of microplastics. However, the design methodologies that we have validated are applicable to the much broader range of polymers found in microplastics pollution.^34,35,106^ Our success in designing a SBP for polyethylene suggests that it should be possible to design SBPs for other common plastics, such as polystyrene or PET. In this context, we note that the interactions of peptides with polymer surfaces have the potential to be influenced by the morphology of the surfaces (e.g., due to processing) in addition to the chemical identity of the polymer (polyethylene versus polypropylene). Specifically, factors such as crystallinity, surface oxidation, chemical cross-linking can potentially influence the ways in which peptides interact with polymers, and additional studies are needed to characterize these impacts. We envisage that SBPs emerging from the design methodologies validated in our study can be incorporated into tools that can be used to detect, filter, capture, or degrade microplastics. We also note that SBPs have applications outside of microplastics pollution, such as increasing the biocompatibility of materials,^107,108^ developing new biomaterials,^109^ controlling crystal growth,^110^ and developing new medical devices.^111^

Fourth, a key barrier to using SBPs for technological applications is the complexity and cost of peptide synthesis. There are two main routes for the synthesis of peptides: chemical and biological. If peptides are chemically synthesized, the high cost of the process will likely limit SBPs to analytical applications. Biological expression of SBPs via engineered microbes, as proposed in recent work,^112,113^ could allow larger scale syntheses. As such, the integration of biophysical modeling with ML screening provides a framework for simultaneously optimizing both binding strength and other peptide properties needed for efficient syntheses, properties that are likely to be different (e.g., solubility or resistance to protease degradation) depending on whether the peptide is prepared using solid-phase peptide synthesis or expressed in bacterial hosts.

## Supplementary Material





## Notes

SI text file: Experimental and computational methods; example outputs from an SMD simulation, visualization of plastic surfaces in simulation, SEM and AFM characterization of polyethylene and polypropylene surfaces used in SMFM experiments, affinity calculations for polypropylene, correlations between peptide affinity and physicochemical properties, analysis and visualization of adsorbed structures, convergence and additional free energy profiles of PBMetaD simulations, adsorption free energies calculated with Umbrella Sampling (PDF)
SI data file: SMD and SMFM data for all peptides, SMFM force histograms and statistics, statistical analysis of SMFM data (XLSX)

## Abbreviations

MM/GBSA: molecular mechanics/generalized born and surface area
EDL: evidential deep-learning
MCTS: Monte Carlo tree search
MD: molecular dynamics
ML: machine learning
MNP: micro-nanoplastic
PBMetaD: parallel bias well-tempered metadynamics
SBP: solid-binding peptide
PBP: plastic-binding peptide, PepBD, peptide binder design
PET: polyethylene terephthalate
SMD: steered molecular dynamics
SMFM: single-molecule force measurements
