# Supplementary material for: Validation of De Novo Designs of Solid-Binding Peptides
Source: ACS Cent Sci. 2026 May 22;12(6):789–806. doi: 10.1021/acscentsci.5c02193 (PMC13306600; doi:10.1021/acscentsci.5c02193)
Supplement: Supplementary file 1 [file oc5c02193_si_001.pdf]

Supplementary Information for:

Validation of *De Novo* Designs of Solid-Binding Peptides

Michael T. Bergman[1 ‡], Fiona Mukherjee[2,3 ‡], Yufan Feng[2], Carol K. Hall[1*,] and Nicholas L. Abbott[2,3*]

[1]Department of Chemical and Biomolecular Engineering, North Carolina State University, Raleigh, NC 27606, USA

[2]Smith School of Chemical and Biomolecular Engineering, Cornell University, Ithaca, New York 14853, USA

[3]Department of Chemistry and Chemical Biology, Cornell University, Ithaca, New York 14853, USA

[‡]These authors contributed equally to this work

*Co-corresponding authors: hall@ncsu.edu, nla34@cornell.edu

Table of Contents

Methods and Materials *S5*

Section 1. The peptides tested in this work S5

Section 2. Assumptions in PepBD Design Approach and Possible Impacts S5

Section 3. Molecular Dynamics Simulation Parameters S6

Section 4. Evaluation of Peptide Affinity to Plastics via Equilibrium MD Simulations S7

Section 5. Generating an Ensemble of Adsorbed Conformations for use in SMD........ S8

Section 6. Calculating Adsorption Free Energies with Steered Molecular Dynamics (SMD)..S8

Section 7. Comparing ΔG_{ads} Between Simulation Methods S11

Section 8. Single Molecule Force Measurements........ S13

Section 9. Statistical Analysis S13

Data Processing and Selection Criteria for Force Analysis S13

Statistical Assessment of Unimodality in Force Distributions S15

Section 10. Preparation of Peptide-Functionalized Atomic Force Microscopy Tips........ S16

Section 11. Materials for Single Molecule Force Measurements........ S17

Section 12. Preparation and Characterization of Polyethylene Surfaces S18

Section 13. Preparation of Spin Coated Polypropylene Surfaces........ S18

Section 14. Characterization of Polymer Surface using AFM and SEM S18

Section 15. Optical Characterization of Polymer Surface S19

Section 16. Effect of Surface Preparation on Adhesion Force........ S19

Section 17. Umbrella Sampling Simulations S20

Section 18. Additional Insights Into the High Affinity of De Novo Designed SBPs: Expanded Section S20

1. The peptides bind by localizing non-polar aromatic and aliphatic amino acid residues near both polyethylene and polypropylene surfaces. S21

2. The contact patterns of amino acid residues differ in subtle ways between polyethylene and polypropylene........ S21

3. Blockiness in sequences of non-polar and polar residues, instead of specific binding motifs, characterize high affinity of peptides for polyethylene. S22

4. The non-polar tetrapeptide WWMX of MCTS 1 and MCTS 2 differs in its association with polyethylene. S23

5. The same triad of non-polar amino acid residues (WWM) shows differential preferences for polyethylene versus polypropylene depending on sequence context. S24

6. Peptides have diverse adsorbed conformations on polyolefin surfaces, with the ensemble of adsorbed conformations differing between polyethylene and polypropylene. S25

Section 19. Identifying Potential Hot Spot Residues in Peptides with High Affinity for Plastic S25

Section 20. Well-Tempered Parallel-Bias Metadynamics (PBMetaD) S26

Section 21. Dynamic Force Spectroscopy for MCTS 1 on Polyethylene S27

Supplemental Figures and Tables *S29*

Figure S1. ....S29
Figure S2. ....S30
Figure S3. ....S31
Figure S4. ....S33
Figure S5. ....S34
Figure S6. ....S35
Figure S7. ....S36
Figure S8. ....S37
Figure S9. ....S38
Figure S10. ....S39
Figure S11. ....S41
Figure S12. ....S42
Figure S13. ....S45
Figure S14. ....S46
Figure S15. ....S47
Figure S16. ....S48
Figure S17. ....S49
Figure S18. ....S50
Figure S19. ....S51
Figure S20. ....S52
Figure S21. ....S53
Figure S22. ....S54
Figure S23. ....S55
Figure S24. ....S56
Figure S25. ....S57
Figure S26. ....S58
Figure S27. ....S59
Figure S28. ....S60
Table S1...S61
Table S2...S62
Table S3...S63
Table S4...S64
Table S5...S65
Table S6...S66
Table S7...S67

Table S8..S68
References *S69*

Methods and Materials

Section 1. The peptides tested in this work

Table 1 lists the PBPs designed by PepBD, MCTS and EDL as well as the control sequences tested in this work. All peptides have 12 residues. More peptides were tested in SMD simulations than in SMFM experiments as the former is cheaper and faster to perform. We used two types of controls: (i) homo-amino acid sequences consisting of either alanine, glutamic acid, or repeats of leucine-lysine; and (ii) randomly generated sequences of 12 amino acids. The randomly generated sequences were created by randomly picking amino acids at each of the 12 positions in the sequence (independently and with equal probability). Five unique peptides were generated in this fashion. These sequences are used as controls because there are no known polyethylene-binding peptides that can serve as a reference. For SMFM, we used the random peptide (Random 5) found to have high affinity in SMD to serve as a high benchmark for determining if the designed peptides have high affinity for polyethylene.We note that the EDL-designed peptides tested in our study (see Table 1) differ from those found in Table 3 of a prior study[1] because our experiments were performed prior to finalization of the prior study. The same EDL method was used to generate all peptides, however, and MD simulations reveal all peptides to have comparable affinities (see below). Accordingly, we judge that the EDL peptides listed in Table 1 permit evaluation of the efficacy of the EDL-based peptide design methodology.

Section 2. Assumptions in PepBD Design Approach and Possible Impacts

As discussed in the main text of our manuscript, PepBD optimizes peptide adsorption enthalpy and does not explicitly consider peptide conformational entropy or solvent restructuring. While the latter two effects contribute to peptide adsorption, quantifying these effects requires simulations that are too computationally intensive for peptide screening. The omission of these

factors in PepBD thus represents a necessary simplifying assumption. However, several lines of evidence support the use of enthalpy as a useful surrogate for binding affinity in molecular design. First, most computational approaches to biomolecular design, including nearly all protein–ligand docking methods, rely on enthalpy-based scoring functions (e.g., van der Waals, electrostatic, and hydrogen-bonding interaction energies) and neglect entropy[2–6]. This is due to the difficulty of calculating entropy. While there is no thermodynamic requirement that a strong binding enthalpy will lead to a strong binding free energy, there has been much success in designing biomolecules by optimizing binding enthalpy[3]. Of particular relevance, the PepBD method has been used previously to design peptides with high affinity for various proteins[7–10]. Second, computational studies of peptide adsorption to surfaces have reported correlations between binding enthalpy and free energy. For example, Welch et al. calculated both binding enthalpy and free energy for all 20 amino acids (as GXG tripeptides) adsorbing to graphene surfaces and found that tripeptides with more favorable binding enthalpies generally exhibited more favorable binding free energies (see Table VII of ref. 11). Our results also demonstrate that optimizing adsorption enthalpy can be an effective strategy for discovering SBPs for polyethylene and polypropylene.

Section 3. Molecular Dynamics Simulation Parameters

Molecular dynamics simulations were prepared using tLEaP[12] in Amber, converted to Gromacs format using Parmed[13], and run with Gromacs version 2019.6[14]. Bonds to hydrogen were constrained using LINCS[15]. Long-range electrostatic interactions were treated using the particle mesh Ewald method[16]. Temperature in NVT and NPT simulations was controlled using the velocity scaling thermostat[17], with separate thermostats for water and non-water atoms. Pressure in NPT simulations was controlled using the Berendsen barostat[18] with a time constant of 5 ps, semi-isotropic coupling so that the simulation box dimension normal to the plastic surface could

change independently of the dimensions parallel to the surface, and an isothermal compressibility of 4.5×10^{-4} in all three directions. The simulation time step was 2 fs. The TIP3P[19] model was used for water, GAFF[20] parameters were used for plastic with partial charges taken from previous work[21], and the ff14SB force field[22] was used for peptides. Methanol was parameterized by obtaining partial charges using the R.E.D. Server[23] and taking all other parameters from GAFF[20]. Atomistic models of plastic surfaces were taken from previous work[21] and are shown in Figure S10. Position restraints with a force constant of 5000 kJ/mol/nm^2 were added to all carbon atoms in the plastic to maintain the rectangular geometry of the plastic surface during all simulation stages.

Section 4. Evaluation of Peptide Affinity to Plastics via Equilibrium MD Simulations

The computationally discovered peptides were first evaluated in high-throughput equilibrium MD simulations prior to the SMD simulations described in this work (see Figure 1). The equilibrium MD procedure has been discussed in previous publications[1,24], and we briefly summarize the methods here. A small ensemble of system conformations where a peptide is adsorbed to a plastic surface is generated per the section "Generating an Ensemble of Adsorbed Conformations for use in SMD" in the Main text. Each conformation is simulated for 1 ns at 300K in the NVT ensemble, then the adsorption free energy is calculated with Amber's MMGBSA tool[13]. We then simulate the eight systems with the lowest binding free energy for an additional 4 ns and recalculate the adsorption free energy. This procedure is performed to identify the most stable adsorbed conformation. Of the eight extended MD simulations, the system with the lowest adsorption free energy is assigned as the peptide affinity since this is predicted to represent the peptide in one of its most stably adsorbed conformations. Uncertainty in free energy calculations for each plastic was estimated by repeating this procedure for 12 peptides and calculating the mean absolute error.

Section 5. Generating an Ensemble of Adsorbed Conformations for use in SMD

An ensemble of adsorbed conformations for each peptide was obtained with the following procedure, which we have detailed previously[1,24]. The peptide and plastic surface were merged into a single system using tLEaP[12], then the peptide was translated 5 Å above the plastic surface using VMD[25]. TIP3P water was added 15 Å above the peptide and 10 Å below the plastic surface using tLEaP. The system was energy-minimized using steepest descent until the maximum force was less than 1000 kJ/mol/nm. The system was equilibrated by running an NVT simulation for 100 ps at 300K, then an NPT simulation for 200 ps at 1 bar and 300K. Next, the system was heated to 550K for 200 ps in the NVT ensemble before running a 10 ns production simulation at the elevated temperature. The high temperature allowed the peptide to rapidly transition between different adsorbed conformations. To prevent the peptide from diffusing away from the surface, we added a harmonic restraining potential using the UPPER WALL utility in PLUMED[26]. The UPPER WALL pushed the peptide back towards the plastic surface when the distance between the peptide center of mass and the top of the plastic surface exceeded 1 nm. From the high temperature simulation, sixteen representative adsorbed conformations were obtained by performing k-means clustering in CPPTRAJ[13] on the peptide's backbone alpha carbons to obtain sixteen clusters, then selecting a representative conformation from each cluster.

Section 6. Calculating Adsorption Free Energies with Steered Molecular Dynamics (SMD)

The adsorption free energy (ΔG_{ads}) of peptides to polyethylene and polypropylene surfaces was evaluated via steered molecular dynamics (SMD) simulations. The SMD process is shown schematically in Figure 2A. The starting adsorbed conformations were obtained as described in the previous section. Each configuration was solvated by adding a TIP3P[19] water box with tLEAP[12] that extended roughly 50 Å away from the top of the plastic surface and 10 Å from the bottom surface. A smaller layer of water was placed under the plastic surface to accelerate simulations.

Systems were equilibrated as in the prior section. SMD was performed in the NVT ensemble using the MOVINGRESTRAINT utility of PLUMED, which adds an external bias in the form of a time-dependent harmonic potential (spring constant of 4 kJ/Å^2). At the start of the simulation, the minimum of the harmonic potential was set at the starting distance between the top of the plastic surface and the peptide center of mass after NPT equilibration. The location of the minimum was then moved away from the plastic surface at a rate of 0.5 nm/ns over 5 ns, for a total distance of 2.5 nm. This external bias induces the peptide to desorb. The work performed by the bias at each time step was calculated as the product of the peptide displacement and the average of the forces at the beginning and end of the time step. The total worked performed by the bias is then the sum of the works from each time step (Figure 2B). This entire procedure (equilibration plus SMD) was repeated three (polyethylene) or six (polypropylene) times for each starting conformation, giving a total of 48 (polyethylene) or 96 (polypropylene) simulations for each peptide-plastic pair. Fewer simulations were performed for polyethylene as ΔG_{ads} changed insignificantly when increasing the number of simulations from 48 to 96 (Table S1).

Using SMD simulations, we calculated ΔG_{ads}. The distribution of desorption works from all SMD simulations is related to ΔG_{ads} via the Jarzynski relation[27], $e^{-\frac{\Delta G_{ads}}{RT}} = \langle e^{-\frac{W}{RT}} \rangle$, where T is the system temperature, R is the gas constant, W is the work performed by the harmonic potential, and $\langle \ldots \rangle$ denotes an average over all simulations. An example of a work distribution is shown in Figure 2B for one peptide-plastic pair. ΔG_{ads} calculated via this procedure changed by no more than 6 kcal/mol for either polyethylene or polypropylene when the entire procedure was repeated for four peptides (Table S2). We deemed this uncertainty to be acceptable for this stage of peptide evaluation. The maximum difference in ΔG_{ads} between two runs over the four peptides (6.4 kcal/mol for polyethylene and 5.7 kcal/mol for polypropylene) was used as a conservative estimate

for uncertainty in ΔG_{ads}. We account for this uncertainty when determining the correlation between SMD and SMFM measurements (shaded region in Figure 5) by performing linear regression with the exact SMD ΔG_{ads}, then shifting all points on the linear fit in the positive or negative x-direction by the appropriate uncertainty in ΔG_{ads} for each plastic.

We note that peptide affinity is not quantified by $F_{pull-off}$ in SMD (see Figure S1 for definition) like it is in SMFM (see below) for multiple reasons. First, there is no direct relationship between the forces evaluated in non-equilibrium events and equilibrium properties like ΔG_{ads}, akin to the relationship established between non-equilibrium work and ΔG_{ads} by the Jarzynski equation. Second, SMD simulations desorb the peptide at a speed roughly six-orders of magnitude faster than in SMFM. Thus, SMFM and SMD likely sample different desorption pathways with different associated forces. Third, SMD simulations may have multiple force peaks (Figure S2) which do not occur in SMFM. The result of these factors is that $F_{pull-off}$ from SMFM correlates better with ΔG_{ads} than with $F_{pull-off}$ from SMD (compare Figure 5 and Figure S28).

While bidirectional protocols may give better convergence[24], they are challenging to apply to peptide adsorption. The collective variable that guides the SMD pathway is the distance between the peptide center of mass and the top of the plastic surface. However, the peptide conformation also plays a key role. Figure 9 clearly shows this: at a fixed distance from the surface, the free energy of the system varies greatly depending on the peptide's radius of gyration or the position of the individual amino acids. This complicates bidirectional SMD, as it becomes unclear when to end the simulations. The simulation cannot be terminated at a small distance, since the peptide conformation may be constrained. This issue does not occur when desorbing the peptide, where the starting structures are obtained from equilibrium simulations.

While SMD may give biased estimates of ΔG_{ads}[28], two results support the use of SMD for peptide ranking. First, ΔG_{ads}^{SMD} does not vary significantly between independent simulations of the same peptide. In Table S2, we present binding affinity results for multiple, independent simulations of the same peptide/surface, and these results were reproducible within an error range of at most 6 kcal/mol, or roughly 25% of the magnitude of ΔG_{ads}^{SMD} (Table S2). Second, we found a strong linear correlation between ΔG_{ads}^{SMD} and $F_{pull\text{-}off}$ of SMFM (Figure 5). Additionally, we retested peptides using umbrella sampling (see SI Section 17), and found similar correlations with SMFM as with SMD.

Section 7. Comparing ΔG_{ads} Between Simulation Methods

We used four different simulation methods to calculate the ΔG_{ads} of adsorption of peptides to plastic surfaces. Each simulation method has its own set of underlying assumptions and/or limitations. For this reason, the magnitudes of ΔG_{ads} values calculated using different simulation methods should not be directly compared. Instead, the relative values of ΔG_{ads} (compared to other peptides) can be compared for a given method of simulation. Below we provide a brief description of each simulation method to note the key assumptions and limitations, and refer the reader to relevant literature for additional details.

- **MM/GBSA** calculates ΔG_{ads} by measuring the energy difference between the state where the peptide is adsorbed to the plastic surface, versus the state where peptide and surface are in the same intramolecular configuration but infinitely separated. This MM/GBSA calculation is paired with normal mode analysis to estimate the conformational entropy change due to peptide adsorption (we note that PepBD excludes normal mode analysis because of the large computational burden). This calculation is repeated over many different adsorbed peptides states and averaged to calculate ΔG_{ads}. This procedure makes

a number of assumptions: water and ions in solution are modelled in a mean field approach using the Generalized Born model, hydrophobic effects are quantified based on the solvent-exposed surface area, the peptide is assumed to adopt similar conformations in solution and when adsorbed, and normal mode analysis coarsely approximates the full conformational freedom of a peptide. Additional details on this procedure can be found in ref[6].

- **SMD** calculates ΔG_{ads} using a non-equilibrium procedure wherein the peptide is repeatedly desorbed from the plastic surface from many different starting adsorbed conformations, calculating the work performed to desorb in each simulation, then applying the Jarzynski equation[27]. While there are no intrinsic assumptions in this procedure (i.e., entropic and solvent effects are accounted for), in practice, the method tends to overestimate ΔG_{ads} due to finite sampling of peptide desorption pathways. Additional details can be found in ref[28].
- **Umbrella sampling** calculates ΔG_{ads} by holding the peptide at different distances from the plastic surface using a harmonic potential, combining the distributions of forces applied by the harmonic potential at each position to obtain a potential of mean force (PMF), and then integrating the PMF to obtain ΔG_{ads}. Similar to SMD, this procedure makes no inherent assumptions that prevent accurate calculation of ΔG_{ads}. However, accurate calculations of ΔG_{ads} require ergodic sampling at each distance from the surface of all degrees of freedom. This does not typically occur due to the many conformational degrees of freedom of a peptide. If the sampling is insufficient, ΔG_{ads} can be inaccurate. Unlike SMD, these inaccuracies will not typically be biased. Additional details can be found in ref[29].
- **PBMetaD** calculates ΔG_{ads} by calculating a PMF force then integrating it, similar to umbrella sampling. It obtains this potential of mean force by adding a time-dependent

potential that accelerates peptide diffusion to and from the surface as well as peptide configurational degrees of freedom. Accelerating sampling of peptide conformational degrees of freedom typically reduces sampling issues compared to umbrella sampling. This makes PBMetaD calculations of ΔG_{ads} the most reliable of all methods used in this study, but inadequate sampling of slow degrees of freedom can still introduce inaccuracies. Additional details can be found in ref[30].

Section 8. Single Molecule Force Measurements

Adhesion force measurements were performed using a Nanoscope IIIa Multimode AFM equipped with a fluid cell (Veeco Metrology Group, Santa Barbara, CA). Triangular-shaped silicon nitride cantilevers (Bruker, MSCT-UC) were used and functionalized as described above. The nominal spring constant of the AFM tips used was 0.01 N/m. The spring constants of the cantilevers were calibrated using the thermal tuning method on an Asylum MFP-3D (Santa Barbara, CA) instrument and determined to be 0.028 ± 0.008 N/m. Force measurements were performed at room temperature. Unless otherwise stated, force curves were recorded using a constant contact time of 500 ms, and retraction and approach speeds of 1000 nm/s, which corresponds to loading rate of 28 ± 8 nN/s. All measurements were performed in aqueous PBS. A J-type scanner was used for the force measurements. These experimental parameters are summarized in Table S3.

Section 9. Statistical Analysis

Data Processing and Selection Criteria for Force Analysis

The histograms of adhesion force measurements reported in this paper are the averages of three independent peptide functionalized tip-substrate (polyethylene or polypropylene) pairs (N=3). Each histogram contains between 300-1000 pull-off force curves, the pull-off forces reported for

each peptide-polymer pair are values averaged across three independent measurements. See the sheet "SMFM Per Peptide Data" in the SI Data File SMFM SMD (SI data file) for the exact number of pull-off forces measured for each peptide sequence-plastic surface pair, and the sheet "SMFM Per Peptide Gaussian Fits" in the SI data file for the Gaussian fits (fitting was done using the Origin software). We performed t-tests to probe the statistical significance of differences in mean adhesion forces for the peptides that were screened for high binding to polyethylene and polypropylene surfaces (Table 1). The t-test results are provided in the sheet "SMFM Statistical Analysis" in the SI data file.

Force-distance curves were pre-screened to select only those displaying a single rupture event during tip retraction, consistent with single molecule unbinding. Curves that exhibited multiple peaks were excluded, as were curves showing no adhesion events (the number of excluded events was < 1% for each peptide-plastic pair as shown in Figure S7 and Table S4). For the short tether (SSMCC) used in this study, a single rupture event serves as a reliable indicator of a single-molecule interaction, as validated by Ma et al.[39]. The selected single-rupture forces were compiled into histograms and fitted with a Gaussian distribution to extract the most probable unbinding force ($F_{pull\text{-}off}$), following procedures reported in past papers[31,34–36]. We also tested whether these histograms showed multimodality using Hartigan's dip test as described in the relevant section below.

As shown in Figure S8, increasing peptide immobilization density from 0.05% to 0.5% (shown for representative peptide MCTS 1 on polyethylene) results in a progressive broadening of the force distribution and decrease in Gaussian fit quality (R^2 = 0.96 (0.1%), and 0.82 (0.5%), respectively), which we attribute to unresolved multi-peptide binding events at higher densities.

Accordingly, adhesion forces reported in this manuscript were obtained using 0.1% peptide density to best approximate single-molecule unbinding.

Statistical Assessment of Unimodality in Force Distributions

To test whether the measured rupture force distributions are unimodal, we performed Hartigan's dip test[37] for unimodality on each force histogram. The dip test measures the deviation of the cumulative distribution of the force histogram from the shape of a single peak. When $p < 0.05$, the deviation is too large to be explained by random variation, indicating multiple populations with different unbinding forces.

Hartigan's dip test was implemented using the "diptest" Python package, which computes the dip statistic and estimates p-values. For each trial, the binned histogram of force distribution raw data were tested at a significance level of $\alpha = 0.05$. A p-value ≥ 0.05 indicates that the distribution is consistent with unimodality, while p-value < 0.05 provides evidence of multimodal structure.

We have included the results in an Excel sheet titled "Test for unimodality" in the revised SI Data File. Overall, all individual trial force distributions (for 0.1% peptide concentration on polyethylene and polypropylene) passed the dip test ($p > 0.05$), supporting the interpretation that the measured force histograms do not show multimodality.

Additional Evidence Supporting the Interpretation of the Pull-Off Forces Reported in our Measurements as Arising from the Interactions of Single Molecules, and Discussion of the Magnitudes of the Forces.

To provide support for our conclusion that the pull-off forces measured in experiments shown in the main text arise from the interactions of single peptide molecules with plastic surfaces, we performed measurements in which we used peptide surface densities on the AFM tip that were

below and above 0.1% (the density used in experiments reported in the main text). The plot of adhesion force (pN) vs. peptide density (%) is shown in Figure S5. As discussed in the main text, these results support our conclusion that we are measuring single-molecule interactions at surface densities of 0.2% and below. To provide additional evidence that the pull-off forces reported in the main text are mediated by the peptides immobilized on the AFM tip, we performed two additional control experiments whose results are shown in Figure S6. Briefly, we measured adhesion forces between polyethylene surfaces and AFM tips at different stages of peptide immobilization including 1. AFM tip functionalized with a mixed self-assembled monolayer (SAM) formed from 1% EG4N (99% EG4OH (ethylene glycol monolayer)). The adhesion measurement with polyethylene surface was performed in 10 mM PBS buffer, and 2. AFM tips that were subsequently SSMCC functionalized.

Section 10. Preparation of Peptide-Functionalized Atomic Force Microscopy Tips

Peptide sequences were synthesized via solid-phase methods by Biomatik and immobilized as detailed previously[31–34]. AFM tips with triangular-shaped cantilevers with nominal spring constants of 0.01 N/m were coated with a 2-nm layer of titanium and a 20-nm layer of gold by physical vapor deposition using an electron beam evaporator[31–34]. The peptide sequences were immobilized on the AFM tips following a protocol reported in prior publications[31–34]. In brief, we immobilized each peptide sequence on mixed monolayers terminated in EG4OH and EG4N groups, using a heterobifunctional cross-linker SSMCC. The estimated length of the SSMCC tether is ∼1 nm[32]. SSMCC reacts with the amine-terminal group of the EG4N component of the mixed monolayer and the free thiol group from a cysteamine (NH_2-CH_2-CH_2-SH) modification to the c-terminus of the peptide sequence. A low density of surface immobilized peptides on the AFM tip was obtained by using a mole fraction of EG4N of 0.001 (or 0.1%) in the solution from which

the mixed monolayers were formed. This approach allowed us to measure adhesive interactions between single peptide molecules immobilized onto mixed monolayers on individual AFM tips and the plastic surface (control experiments are provided in Figures S4-S8). Previous work has shown that maleimide activated EG4N/EG4OH mixed monolayers do not generate measurable adhesive forces with non-polar AFM tips in aqueous PBS buffer[32].

Section 11. Materials for Single Molecule Force Measurements

Tetraethylene glycol thiols terminated in hydroxyl (EG4OH) or amine groups (EG4N) were purchased from Prochimia (Gdynia, Poland). PBS (Corning, NY; Dulbecco's Phosphate-Buffered Saline, 1X with calcium and magnesium, pH 7.4), methanol (anhydrous, 99.8%), and ethanol (reagent, anhydrous) for preparing thiol solutions were purchased from Sigma-Aldrich (Milwaukee, WI). Sulfosuccinimidyl-4-(N-maleimidomethyl) cyclohexane-1-carboxylate (SSMCC) was purchased from Thermo Fisher Scientific (Rockford, IL). All peptide sequences were purchased from Biomatik (Wilmington, DE). Ethanol (anhydrous, 200 proof) used for rinsing was purchased from Decon Labs (King of Prussia, PA). All chemicals were used without additional purification. AFM tips (triangular shaped, nominal spring constant of 0.01 N/m) for force measurements were purchased from Bruker Nano (Camarillo, CA). Silicon wafers were purchased from Silicon Sense (Nashua, NH). High Density Polyethylene (HDPE-grade) strips, Catalog No. 1546707, Lot No. R149K0 were purchased from Sigma-Aldrich (UPS Store, Milwaukee, WI). Amorphous polypropylene beads, average Mw ~12,000 (Cas No 9003-07-0) and o-xylene were purchased from Sigma-Aldrich (Milwaukee, WI).

Section 12. Preparation and Characterization of Polyethylene Surfaces

The HDPE sheets were used as polyethylene substrates without further treatment after rinsing with ethanol and purified water (Milli-Q System, 18.2 MΩ cm resistivity at 25 °C; Millipore, Bedford, MA) for three cycles.

Section 13. Preparation of Spin Coated Polypropylene Surfaces

Polypropylene beads were dissolved in *o*-xylene at 130 °C at a concentration of 6 wt.%. The clear and transparent solution was spin coated on a clean transparent glass slide to make a thin film. The spin coating condition was performed in two steps: a slow step of 400 rpm for 60 s, followed by a fast step of 3000 rpm for 60 s[38]. After the spin coating, the thin films were heated in an oven at 160 °C for 20 min.

Section 14. Characterization of Polymer Surface using AFM and SEM

Physical characterization of the plastic surfaces using AFM and scanning electron microscopy (SEM) is shown in Figure S11. Inspection of Figure S11A reveals that the commercial polyethylene surfaces has porous structures. However, the porosity is present at a length scale (~10-20 µm) (Figure S11A) that is approximately 10× larger than our AFM scan areas (1 µm × 1 µm) (images in Figure S11C, I). Within 1 µm × 1 µm regions of the surfaces, both SEM and AFM imaging reveal relatively smooth surfaces (with roughnesses R_{RMS}: 42.4 ± 11.2 nm for polyethylene (Figure S11D), 17.7 ± 6.8 nm for polypropylene (Figure S11I). Since AFM pull-off force measurements probe local surface interactions within these scan areas, the presence of porosity on the ~10-20 µm scale is not expected to directly influence the measured adhesion forces. The AFM tip radius is approximately 10-40 nm[23], and the characteristic length of the area of contact between the AFM tip and plastic surface is expected to be of a similar magnitude (i.e., much smaller than the scale of the porosity).

Overall, SEM revealed that the commercial polyethylene surfaces used in our study exhibited a topography at ~10-20 µm length scales, which is large compared to the AFM tip radius (~10-40 nm) and unlikely to influence measured adhesion forces.

Section 15. Optical Characterization of Polymer Surface

We acquired optical micrographs using crossed polarizers of each surface (Figure S12 A,B). The commercial polyethylene surface exhibited birefringence confirming their semicrystalline structure. In contrast, the spin-coated polypropylene surface appears dark under crossed polarizers (Figure S12 B), consistent with an amorphous structure. These results are consistent with the crystalline polyethylene and amorphous polypropylene surface models used in simulations.

Section 16. Effect of Surface Preparation on Adhesion Force

To evaluate the sensitivity of our adhesion force measurements to surface preparation methods, we measured the adhesion force between MCTS 1 peptide and a commercial HDPE surface that was incubated in 60 v% methanol for 2 h, followed by rinsing three times with water and ethanol. All experimental conditions, including the peptide functionalization density (0.1%) were unchanged.

The mean pull-off force measured on the solvent-treated commercial HDPE surface (after incubation and force measurements in 60 v% methanol) was 702 ± 5 pN (N = 1000) (Figure S12C). This value is in good agreement with the adhesion force measured on the commercial polyethylene surface prior to surface treatment, 698 ± 1 pN (N = 1650), reported in Figure 3C of the main manuscript (reproduced in Figure S12 for ease of comparison). This result indicates that exposure to organic solvents (methanol and ethanol) does not appreciably alter either the magnitude or the distribution (R^2~0.9) of adhesion forces for MCTS 1 on commercial HDPE (Figure S12C). We

conclude that organic solvent treatment does not significantly modify the surface structure of polyethylene to impact peptide binding.

Section 17. Umbrella Sampling Simulations

The affinity of peptides to plastic was evaluated using umbrella sampling (US) to determine if the results better correlated with SMFM data, as described in the Discussion section of the Main text. The collective variable in US was the distance between the peptide center of mass and the plastic surface, just as in SMD. For each peptide – plastic combination, we create 32 windows spaced in 0.5 Å in this distance, thus sampling a total distance range of 16 Å. The first window (i.e., the window with the smallest distance between the peptide and the plastic surface) was set to 4.5 Å for polyethylene and 6 Å for polypropylene. Different distances were used because the greater roughness of the polypropylene surface prevents the peptide from getting as close as it can get to a polyethylene surface. Each target distance was simulated in four independent simulations using conformations extracted from four separate SMD simulations. We simulated each window four times to improve conformational sampling of the peptide. Each window was simulated for 1.5 ns at 300K in the NVT ensemble. The free energy profile as a function of the peptide distance to the surface was then calculated using WHAM[43], with the first 0.5 ns of each simulation discarded since the system was equilibrating. Integration of the free energy profile then gives the adsorption free energy. The standard deviation in the adsorption free energy was calculated using a previously described procedure[43].

Section 18. Additional Insights Into the High Affinity of De Novo Designed SBPs: Expanded Section

The text below is an expanded version of the discussion presented in the main text. Some text below is repeated from the main text for completeness and ease of reading of the SI.

1. The peptides bind by localizing non-polar aromatic and aliphatic amino acid residues near both polyethylene and polypropylene surfaces. We analyzed the distance between the amino acid side-chains and the polymer surfaces (averaged over the ensemble of adsorbed conformations) for all strong-binding-peptides identified in our study (Figure S15, as shown below). Inspection of Figure S15 reveals the locations of individual amino acids (Figure S15B) as well as categories of amino acids (Figure S15A), the latter defined as either ionic (I), polar (P), aliphatic (Al) or aromatic (Ar). This analysis reveals that aromatic (F, W, Y) and aliphatic residues (I, L, M) were consistently localized near the polyolefin surfaces (within 4–5 Å), and that charged residues (particularly K, D, E) were localized substantially away from the surface (i.e., solvent exposed; > 7 Å from the surface). Polar residues (H, S, T, N, Q) were found to be either adsorbed or solvent-exposed. These results provide additional support for our conclusion that peptide binding to both polyethylene and polypropylene surfaces is driven largely by hydrophobic interactions, as inferred from experiments reported in our manuscript (Figure 7), but also that partitioning of charged amino acid residues away from the polyolefin surfaces is a characteristic feature of the strong binding peptides (we return to this point below).

2. The contact patterns of amino acid residues differ in subtle ways between polyethylene and polypropylene. Figure S15 not only reinforces the importance of hydrophobic interactions in the adsorption of the peptides to polyolefin surfaces, but also hints at differences between the binding of SBPs at polyethylene and polypropylene surfaces (Figure 6 of our manuscript shows that the

peptides identified in our study bind more strongly to polyethylene than polypropylene). Inspection of Figure S15A reveals that differences exist in the distributions of ionic residues, polar and non-polar residues on the two polymer surfaces. Specifically, ionic residues K, D and E, and polar residues S and T, localize further from the polyethylene surface than from the polypropylene surface. In addition, non-polar residues, such as I and L, tend to locate closer to the polyethylene surface than polypropylene surface, consistent with differences in hydrophobic contact between the two surfaces. Overall, Figure S15 provides initial support for a physical picture where differences in the locations of ionic and polar residues (extending more towards solution for polyethylene) and non-polar residues (making more frequent contact with polyethylene) reflect the measured variations in affinity of the peptides for polyethylene and polypropylene surfaces; this picture of more effective segregation of charged/polar residues from non-polar hydrophobic residues on polyethylene as compared to polypropylene is consistent with stronger hydrophobic interaction (e.g., via I and L) with the polyethylene surface. The latter proposal receives support from prior studies by Dallin et. al[44] who reported that hydrophobic interactions at ordered aliphatic surfaces are stronger than disordered aliphatic surfaces (the polyethylene used in our study is crystalline whereas polypropylene is amorphous). Finally, we note that differences in segregation patterns of the type discussed above are also evident in comparisons between different peptides sequences binding to the same surface (see points #3 and 4 below).

3. Blockiness in sequences of non-polar and polar residues, instead of specific binding motifs, characterize high affinity of peptides for polyethylene. MCTS 1 and MCTS 2 are two peptide sequences identified by our study as having high affinity for polyethylene (Figure 8A, dark purple data; see below). Inspection of Figure 8A reveals localization of non-polar amino acids near the

polyethylene surface and the partitioning of charged amino acids away from the polyethylene surface for both MCTS 1 and MCTS2, similar to the general trends shown in Figure S15. Inspection of Figure 8A, however, also reveals a characteristic blockiness in the sequence of non-polar and charged amino acid residues within both MCTS1 and MCTS2. We hypothesize that this blockiness plays a key role in enabling the segregation of non-polar and charged amino acid residues in polyethylene-bound high affinity peptides, as discussed in point 2 above, by allowing blocks of non-polar amino acids to adsorb to the polyethylene surface and the sequences of charged residues to loop away from the surface. Specifically, MCTS 1 has two non-polar blocks (WWMM and WFF) while MCTS 2 has a single, large non-polar block at the C-terminus (WWMIFF). The other high affinity peptides identified in our study also exhibited similar blockiness. Overall, these observations support our proposal that a characteristic feature of the high affinity peptides identified in our study is the presence of blocks of non-polar amino acids rather than a motif involving a specific sequence of amino acids (e.g., the (XXYY)n, where X denotes hydrophobic residues, Y denotes cationic residues, found in α-helical antimicrobial peptides that bind to non-polar surfaces[45,46]). An important corollary to this proposal is that many different blocks of non-polar amino acids can achieve strong binding to polyethylene surfaces. This idea is supported by the Hamming similarity matrix[47] shown in Figure S25, which reveals that the strong-binding peptides validated in our study have diverse amino acid compositions despite comparable (strong) binding performance.

4. The non-polar tetrapeptide WWMX of MCTS 1 and MCTS 2 differs in its association with polyethylene. Both MCTS 1 and MCTS 2 contain a tetrapeptide block of four non-polar amino acid residues (WWMM or WWMI, respectively; see Figure S16 below). Interestingly, inspection

of Figure S16A reveals that WWMM of MCTS 1 localizes to a greater extent at the polyethylene surface than WWMI from MCTS 2, despite the fact that I of MCTS 2 is considered more hydrophobic than M of MCTS 1. This difference in localization is noteworthy also because WWMM of MCTS 1 is flanked on either side by two ionic amino acid residues (EK and RR) that extend away from the polyethylene surface to a greater extent that the flanking residues for MCTS 2 (DY and FF). Overall, the physical picture that emerges from this comparison of MCTS 1 and MCTS 2 provides additional support for our proposal discussed above that strong binding peptides (we measured MCTS 1 to bind to polyethylene more strongly than MCTS 2) are characterized by adsorbed configurations that effectively segregate non-polar amino acids to the polyethylene surface and charged amino acids away from the surface.

5. The same triad of non-polar amino acid residues (WWM) shows differential preferences for polyethylene versus polypropylene depending on sequence context. The analyses presented above reveal that blocks of non-polar amino acid residues from MCTS 1 and MCTS 2 localize to different extents at the surfaces of polyethylene, consistent with a hydrophobic driving force for adsorption. Additional inspection of Figure S16A reveals that the non-polar triad WWM of MCTS 1 localizes more closely to the surface of polyethylene than polypropylene, but WWM of MCTS 2 localizes closer to polypropylene than polyethylene (highlighted in Figure S16B). This result illustrates how the relative extents of association of a specific non-polar peptide block within a sequence depends on both the overall peptide sequence and the polymer surface properties. As noted above, the surfaces of polyethylene and polypropylene are structurally distinct: polyethylene surface is ordered and crystalline while polypropylene has a rough, amorphous surface with pendant methyl groups.

6. Peptides have diverse adsorbed conformations on polyolefin surfaces, with the ensemble of adsorbed conformations differing between polyethylene and polypropylene. Past studies have hypothesized that a key challenge underlying the design of SBPs, which is distinct from peptides binding to protein pockets, is that SBPs typically adopt a broad range of conformations when adsorbed to surfaces of solids. Our results support this hypothesis, as evidenced by the broad surface-distance distributions, often with multiple peaks, particularly for the charged residues (Figure S15; see also Figure S24). Our results reveal that the adsorption free energy landscapes are characterized by broad minima, and that MCTS 1 assumes substantially different ensembles of adsorbed conformations on polyethylene and polypropylene (Figure S24; see also Figure 9). MCTS 1 more readily adopts adsorbed conformations that extend along the surface (larger R_{gyr}) when adsorbed to polyethylene than polypropylene (z = 0.5 nm), which correlates with experimentally measured stronger adhesion of MCTS 1 on polyethylene.

Section 19. Identifying Potential Hot Spot Residues in Peptides with High Affinity for Plastic

To identify potential hot spot residues, we assume that a hot spot will consistently interact with the plastic surface. Thus, we can analyze the adsorbed conformations obtained for a peptide: plastic pair and categorize residues that are consistently proximal to the plastic surface as being a possible hot spot. The validity of this approach is supported by the ensemble of adsorbed structures of the LK-12 peptide (sequence: LKLKLKLKLKLK) adsorbed to polyethylene (Figure S18). Every aliphatic leucine (L) residue is always close to the surface, whereas every charged lysine (K) residue is far from the polyethylene surface and presumably solvent-exposed. To determine if a selected residue is truly a hot spot, we mutate the residue to alanine and repeat SMD. Two changes are expected for a hot spot residue: the mutated residue should no longer contact the plastic surface,

and peptide affinity for the plastic should be significantly reduced. Both changes are predicated on the analysis of polyalanine adsorption to polyethylene (Figure S18). No alanine residue consistently contacts polyethylene, and polyalanine has weak affinity for polyethylene relative to the other peptides.

Section 20. Well-Tempered Parallel-Bias Metadynamics (PBMetaD)

PBMetaD simulations[48] were performed to obtain the free energy surface for the adsorption of the best designed peptide to polyethylene. The system was prepared and equilibrated following the procedure used in SMD with two changes: 1) more water was added so that the water layer extended roughly 9 nm above the polyethylene surface after NPT equilibration, and 2) NVT and NPT equilibration were both performed for 5 ns. PBMetaD was run in the NVT ensemble using PLUMED[26]. Following past work[49], the collective variables were the distances between each side chain center of mass and the top of the polyethylene surface, and the peptide radius of gyration, giving a total of 13 collective variables. An Upper Wall of PLUMED (a harmonic bias potential) was added at 5.5 nm for each side chain distance to prevent the peptide from diffusing to the underside of the plastic surface. Gaussian hills were deposited every 1 ps with an initial height of 2 kJ/mol and a bias factor of 30. The width of the added Gaussian biases was controlled using the adaptive Gaussian method[50], with upper and lower limits of 0.01 and 0.5 nm. We used four multiple walkers[51] (i.e. multiple simulation replicas that communicate with each other) to speed up convergence. The free energy profile for each collective variable, as well as the entire peptide's center of mass distance to the polyethylene surface, was calculated using reweighting[48]. Results were deemed to have converged as changes in the free energy profile were of the order of thermal energy (Boltzmann constant times temperature) as the simulation was extended (Figure S19 and S20).

Section 21. Dynamic Force Spectroscopy for MCTS 1 on Polyethylene

Rupture forces measured by SMFM are inherently non-equilibrium quantities whose absolute magnitudes depend on loading rate, contact time, linker mechanics, and the details of the energy landscape. However, in our experiments, the above mentioned parameters are identical (listed in SI Methods section) enabling differences in mean rupture force across peptides to be interpreted as reflecting differences in the underlying peptide–surface interaction. The relative ordering of pull-off forces serves as a meaningful comparative metric.

We also wish to clarify that we do not equate rupture forces to equilibrium binding free energies, rather we use pull-off forces as a proxy for affinity. A number of prior studies support this approach. Specifically, prior studies performed with identical experimental parameters have found that mean rupture forces correlate with the surface affinity as measured by surface plasmon resonance (SPR)[39]. For example, Wei et al.[39] demonstrated that AFM pull-off forces for peptides on solid surfaces correlate linearly with adsorption free energies, providing strong evidence that pull-off force measurements function as a useful proxy for relative affinity when the SMFM are performed under rigorously controlled conditions. The correlation between magnitude of pull-off force and surface affinity has been found in a number of additional studies[35,40–42]. Finally, the use of pull-off forces as a proxy for surface affinity is also supported by the correlation that we found between ΔG_{ads} (obtained from either SMD or umbrella sampling) and experimental values of $F_{pull\text{-}off}$ (Pearson $r^2 = 0.93$ for polyethylene with SMD data, Figure 5A).

To further validate this interpretation, we performed dynamic force spectroscopy for MCTS 1 on polyethylene by measuring rupture force across retraction speeds from 410, 634, 872, 1000, 1160, 2330 and 3490 nm/s, with other parameters kept constant. The results are shown in Figure S27.

We analysed the data using the Bell-Evans framework, which predicts that the most probable rupture force, F(r) scales linearly with the natural log of the loading rate (r) during retraction. The Bell-Evans equation is,

$$F(r) = \frac{k_B T}{x_\beta} \ln \frac{r x_\beta}{k_0 k_B T}$$

where, F(r) is the most probable rupture force, r is the loading rate, x_β is the barrier height, k_0 is the dissociation rate at zero force, k_B is the Boltzmann constant, and T is the absolute temperature and h is Planck's constant and $\Delta G^{\ddagger}$ is the activation free energy for dissociation of the peptide from the surface. $\Delta G^{\ddagger}$ is calculated from the following equation,

$$-\Delta G^{\ddagger} = k_B T \ln \frac{k_0 h}{k_B T}$$

In Figure S27B, we show that the data obtained for MCTS 1 on polyethylene is described by the Bell–Evans model ($R^2 \sim 0.95$), which is consistent with a single energy barrier governing peptide detachment. Moreover, the activation free energy of the peptide detaching (or unwinding) from the polyethylene surface extracted from the Bell–Evans fit (= 18.5±0.6 kcal/mol) is comparable in magnitude to the ΔG_{ads} obtained from metadynamics simulations (-15.3 kcal/mol).

Supplemental Figures and Tables

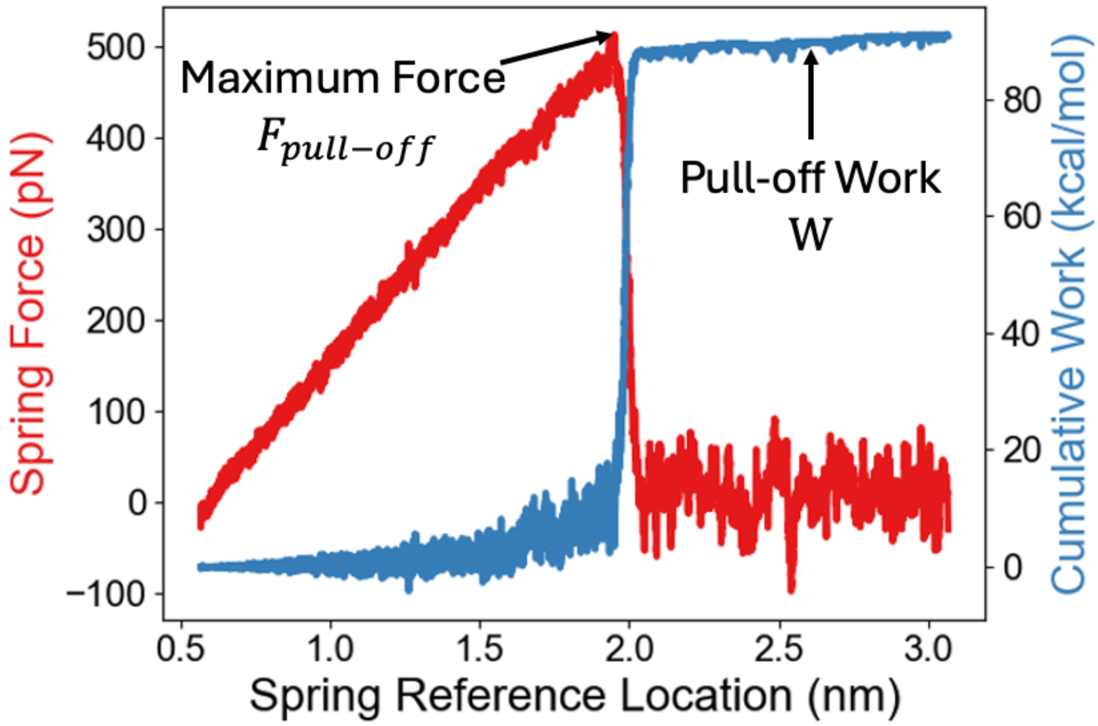

Figure S1. An example force spectrum from SMD. Red curve shows the force applied by the spring as the peptide is desorbed from the plastic surface, and the blue curve shows the total work performed by the spring over the simulation. Labels indicate the values for $F_{pull-off}$ and W.

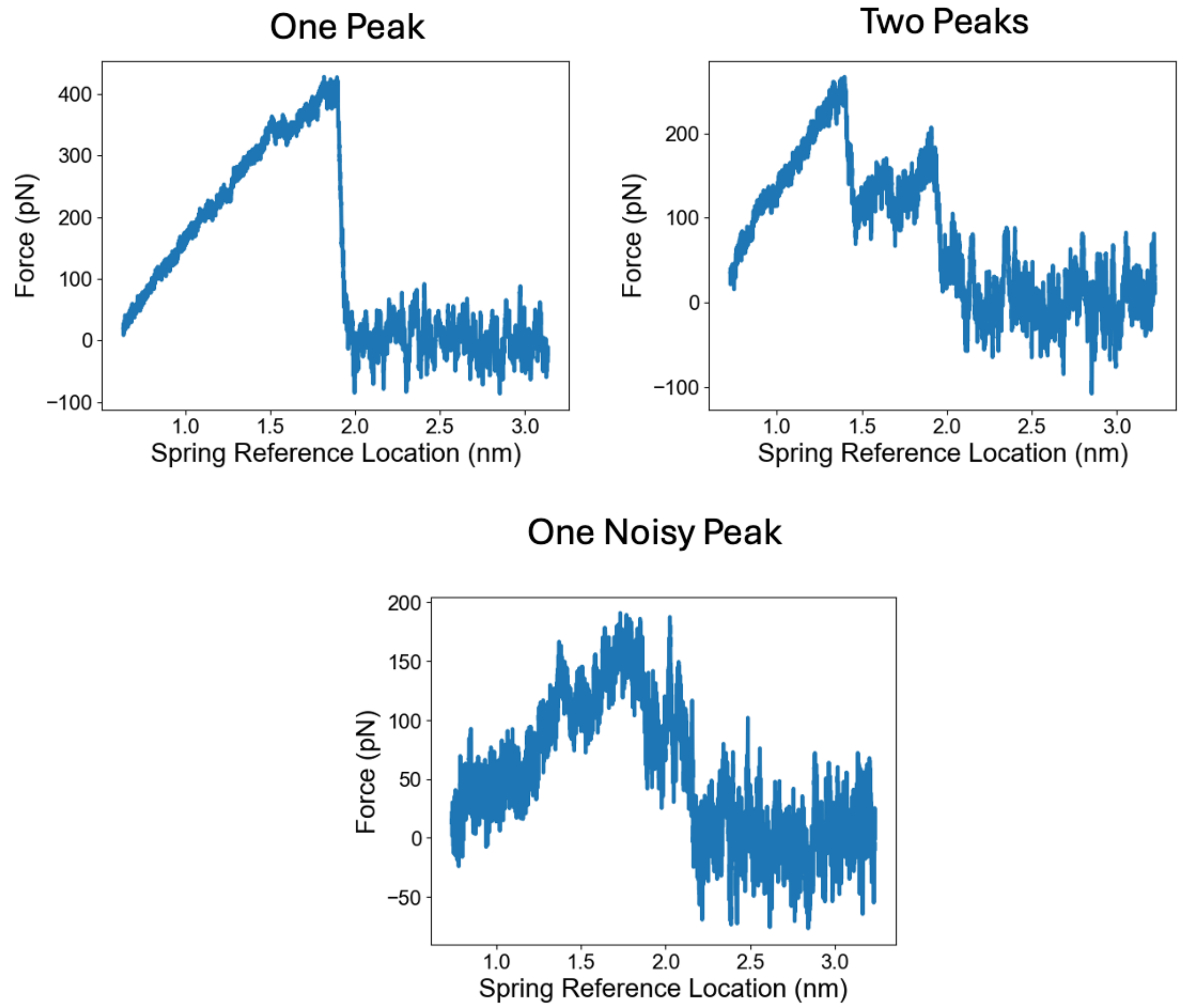

Figure S2. Example force vs. time curves for SMD simulations. Three general categories of force spectra are observed: one force peak, two force peaks, and a noisy force peak. Different force profiles correspond to different desorption pathways.

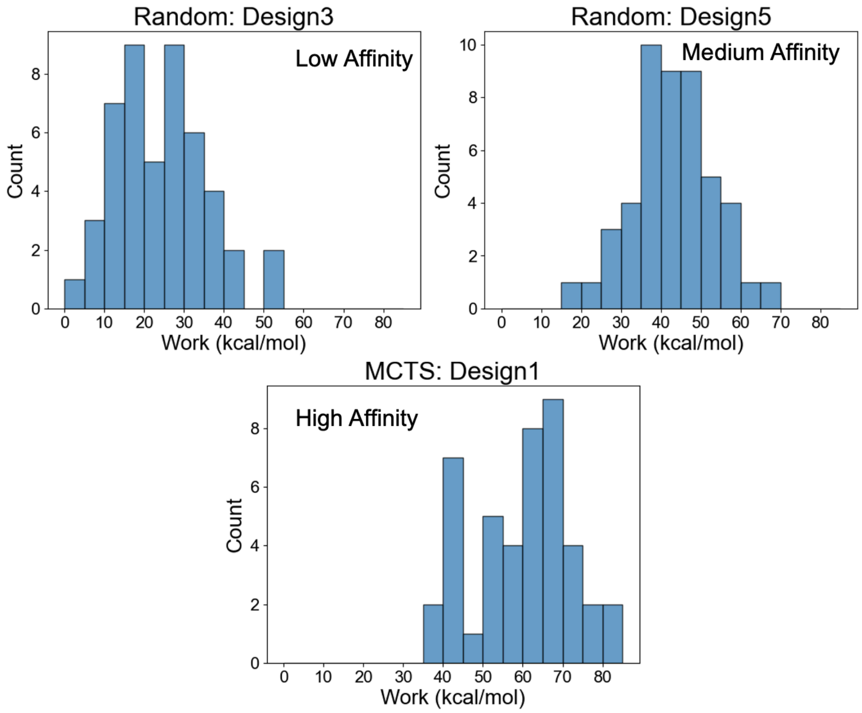

Figure S3. Example work distributions for peptide desorption from polyethylene. One example is given each for peptides with relatively low, moderate, or high affinity.

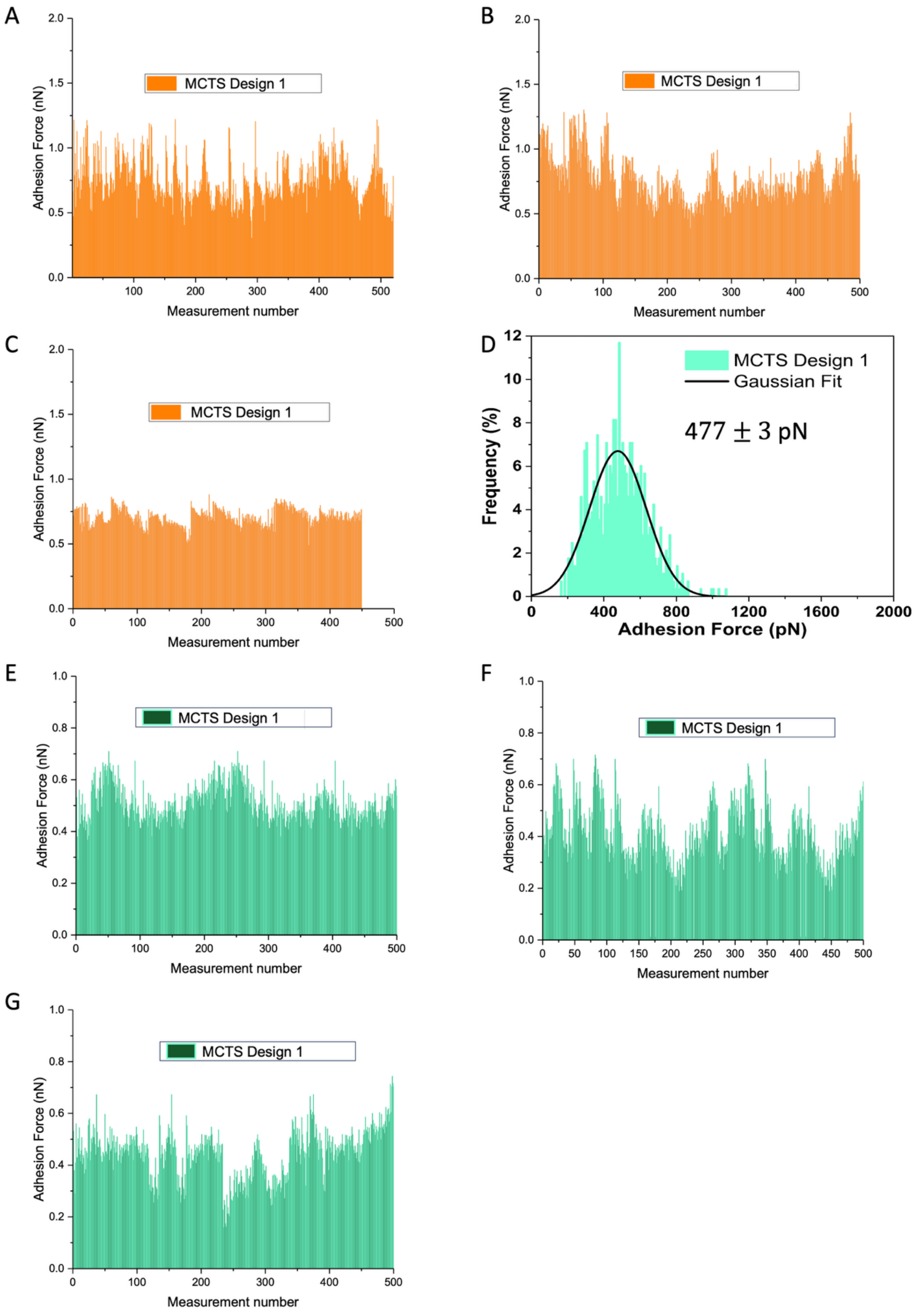
A
B
C
D
E
F
G
MCTS Design 1
Gaussian Fit
477 ± 3 pN
Adhesion Force (nN)
Measurement number
Frequency (%)
Adhesion Force (pN)

Figure S4. Representative raw data used to construct force histograms. SMFM plots shown in Figure 3C of main text or Figure S4D (above) were obtained by immobilizing peptides (0.1%) to an AFM tip and contacting them with polyethylene or polypropylene surface. The measurement number is the number of contacts between the AFM tip and the plastic surface. Measurements were performed in 10 mM PBS buffer. (A-C) Plots show the raw data (N=3) of pull-off forces measured vs measurement number for MCTS Design 1 peptide sequence for polyethylene that was used to construct the histogram (shown in orange) in Figure 3C of main text. (E-G) Plots show the raw data (N=3) of pull-off forces measured vs measurement number for MCTS Design 1 peptide sequence for polypropylene that was used to construct the histogram (shown in green color) in D.

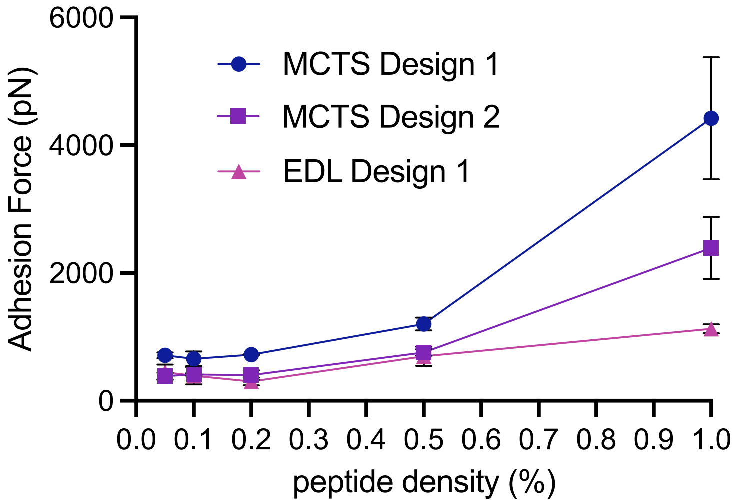

Figure S5. Experimental evidence that single molecule pull-off events are measured when peptide densities on the AFM tip are less than 0.2% (see Methods for a detailed description of the immobilization chemistry). The plots show peptide pull-off forces measured as a function of peptide surface density in PBS for three different peptide sequences, MCTS 1, MCTS 2 and EDL 1 on polyethylene surface. (N=3 for all values). Error bars are indicated.

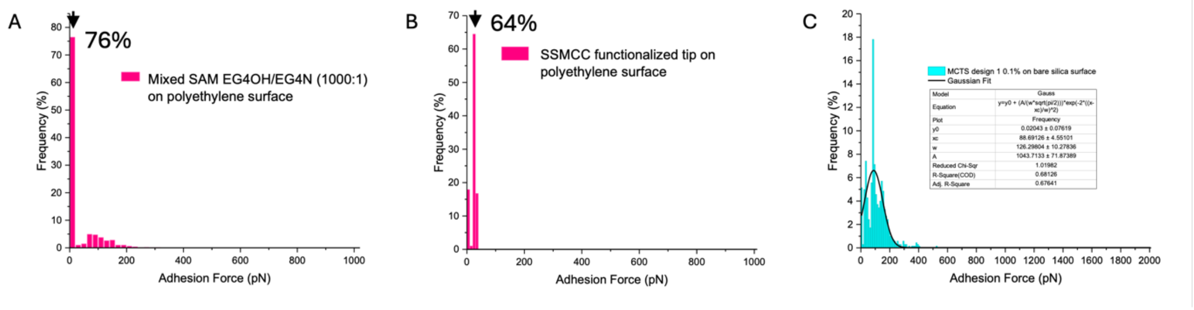

Figure S6. Control experiments that support our conclusion that the force measurements reported in the main text of this manuscript arise from the presence of peptides on the surface of the AFM tip and their interaction with polymer films. (A) and (B) show adhesion force histograms obtained using AFM tips at different steps of the peptide immobilization procedure (prior to attachment of peptides). The AFM tips were interacting with polyethylene surfaces. (A) AFM tip functionalized with a mixed self-assembled monolayer (SAM) formed from 1% EG4N (99% EG4OH (ethylene glycol monolayer)). The adhesion measurement with polyethylene was performed in 10 mM PBS buffer. (B) AFM tip in (A) after subsequent SSMCC treatment. The adhesion measurement with polyethylene was performed in 10 mM PBS buffer. (C) Adhesion force histogram characterizing the interaction of MCTS Design 1 peptide-functionalized AFM tip with a bare silica surface. The observation that the adhesion force histogram measured on glass (C) is comprised of forces that are small compared to those measured on polypropylene surfaces (see Fig S8E) supports our conclusion that a continuous polypropylene film coating was formed on the glass substrate.

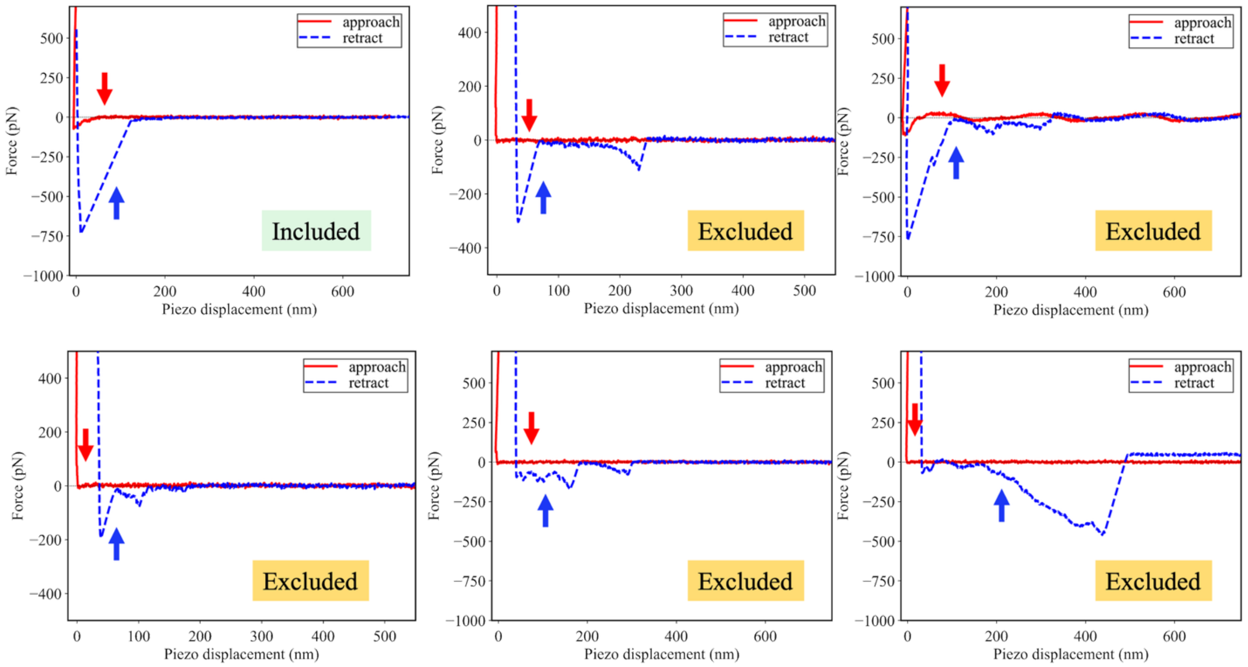

Figure S7. Representative force curves from pull-off force measurements showing one force curve with a single force peak that was included in the analysis (labelled in green) and 5 representative force curves that were excluded from the analysis (labelled in yellow) due to the presence of more than one peak > 10 pN.

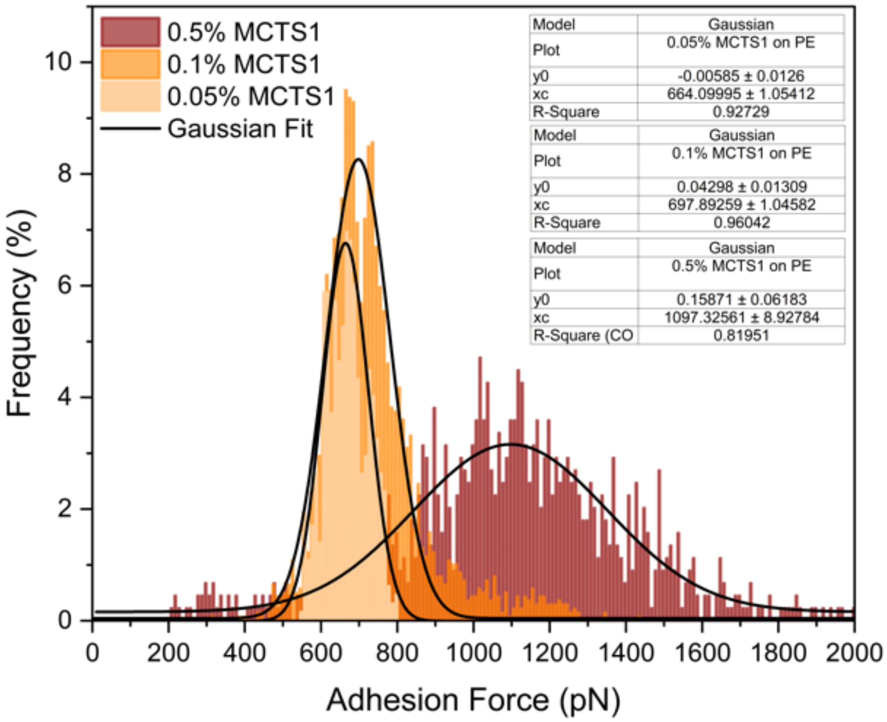

Figure S8. Plot shows an overlay of pull-off force histograms for MCTS Design 1 on polyethylene surface for different concentrations of peptide functionalization of the AFM tip, 0.05%, 0.1% and 0.5%. Note that the plot of 0.1% is duplicated from Main Text (Figure 3C) for direct comparison.

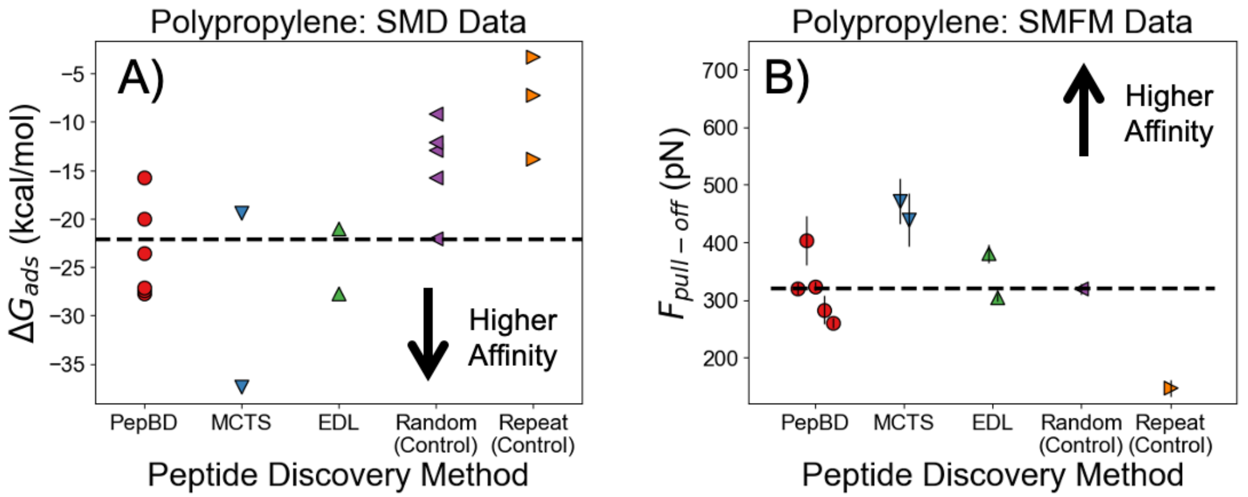

Figure S9. Simulations and experiments both show that the computationally discovered peptides have moderately high affinity for polypropylene. Left plot shows the results from SMD and the right plot shows the results from SMFM. High affinity for plastic is associated with a more negative ΔG_{ads} and a more positive $F_{pull\text{-}off}$. Peptide sequences are provided in Table 1, data for each peptide category is provided in Table 2, and raw data is provided in the SI data file: “SMFM & MD Data” sheet. Error bars for ΔG_{ads} are taken from the maximum deviation in Table S2. Error bars for $F_{pull\text{-}off}$ are 1 standard error of mean (SEM) from 3 replicate measurements.

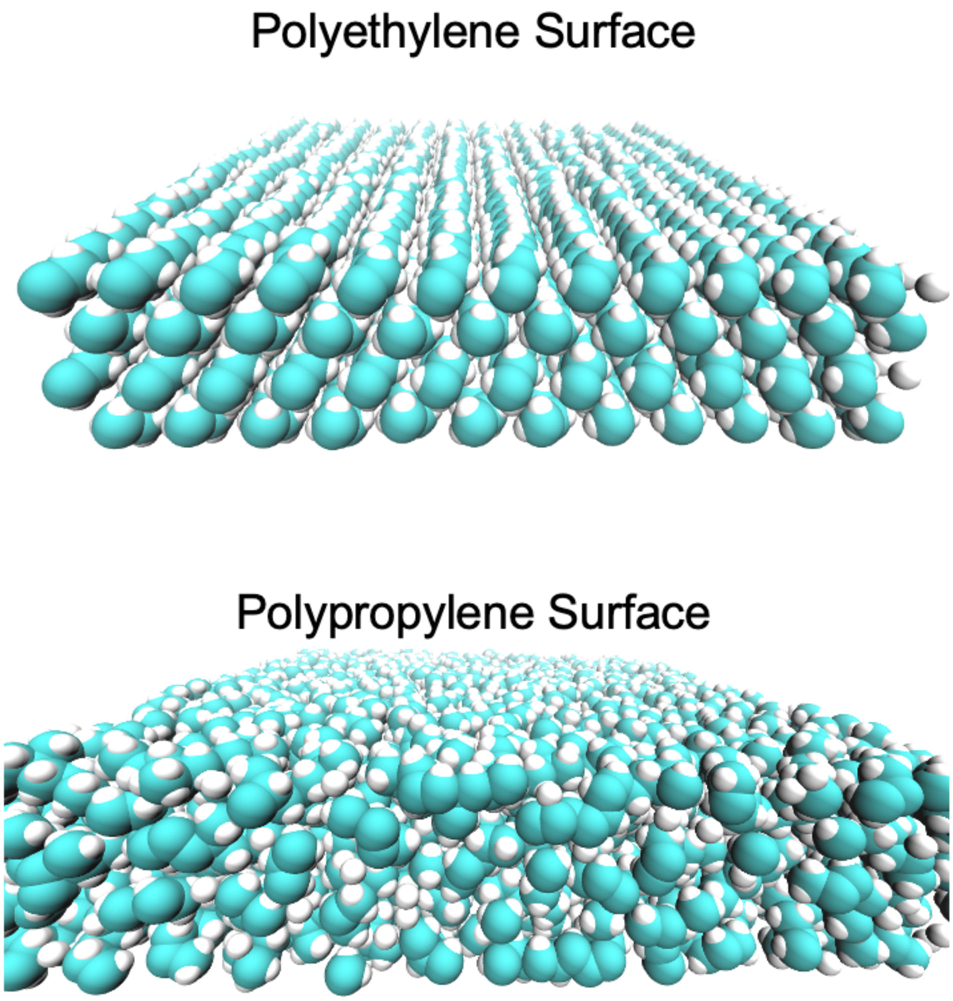

Figure S10. Atomistic structure of polypropylene and polypropylene used in SMD simulations. The polyethylene surface has dimensions of 58.3 Å by 59.5 Å, which are multiples of the crystal lattice dimensions. The polypropylene surface has dimensions of 80 Å by 80 Å. Both surfaces are approximately 20 Å thick. Figure made using VMD.

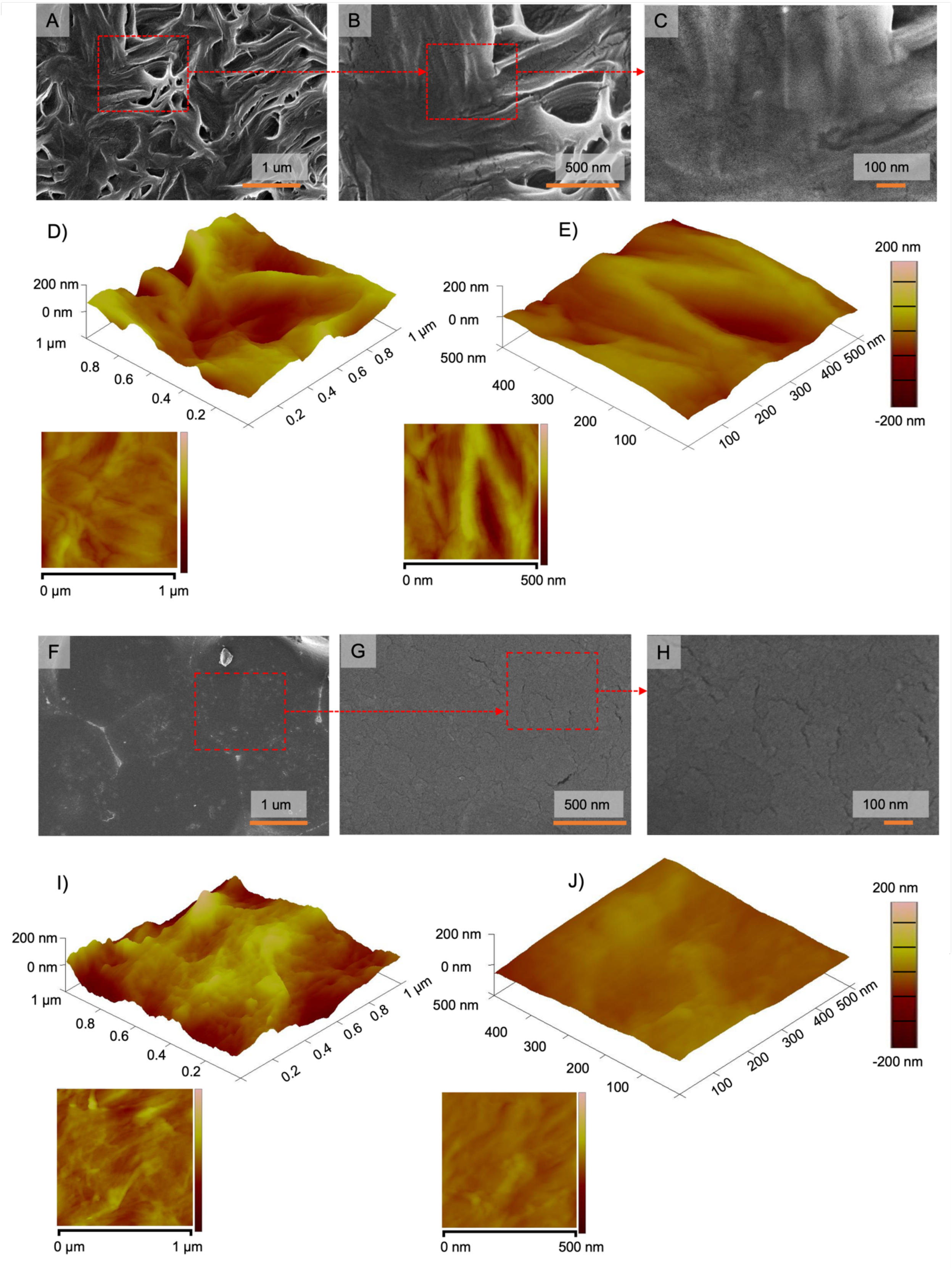
A
1 um
B
500 nm
C
100 nm
D)
200 nm
0 nm
1 µm
0.8
0.6
0.4
0.2
0 µm
E)
500 nm
400
300
200
100
-200 nm
0 nm
F
G
H
I)
J)

Figure S11. Physical characterization of polypropylene and polyethylene materials used in the experiments reported in this paper. (A-C): SEM images of polyethylene film surfaces used for SMFM experiments at different magnifications. SEM images show porous structures at low magnification (A), but flat/smooth apparent surface at higher magnification (C). (D, E): AFM images of polyethylene (inset, phase image) at scan areas of D. 1 μm x 1 μm and E. 500 nm x 500 nm. (F-H): SEM images of polypropylene film surfaces used for SMFM experiments at different magnifications. SEM images show relatively flat/smooth apparent surfaces at all magnifications. (I, J): AFM images of polypropylene (inset, phase image) at scan areas of I. 1 μm x 1 μm and J. 500 nm x 500 nm. AFM imaging was carried out using 0.1 Nm^{-1} (nominal spring constant) cantilevers with silicon nitride tips (described in SMFM methods section in main text) with a nominal radius of ~10-40 nm (according to Bruker MSCT-UC product specification). The tip velocity was maintained at 50 nm/s, scan rate was between 0.05-0.1 Hz. The root mean squared surface roughness (R_{RMS}) for polyethylene and polypropylene films were measured from the 1 μm x 1 μm images (D & I) to be 42.4 ± 11.2 nm and 17.7 ± 6.8 nm, respectively.

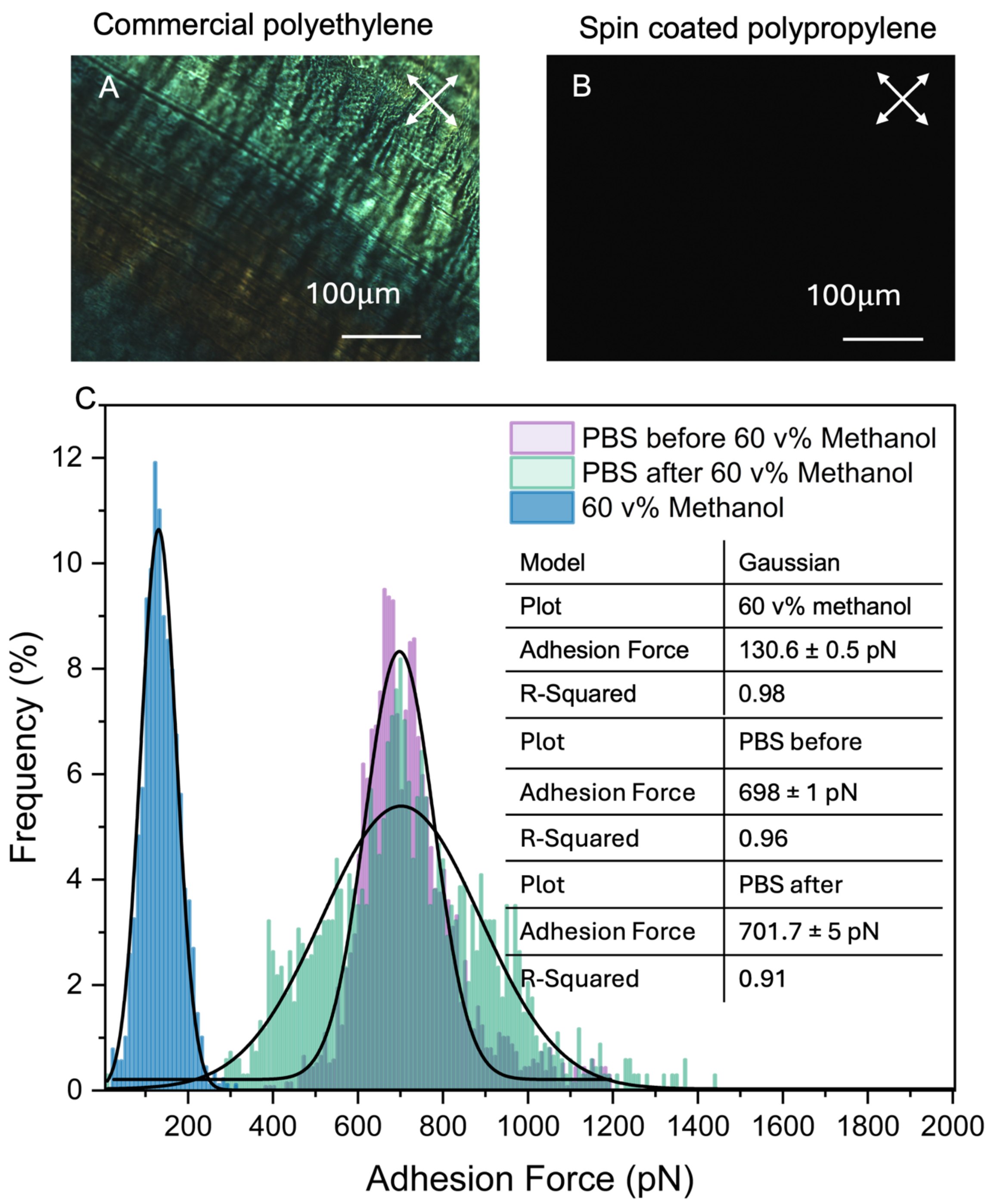

Figure S12. Optical micrographs (crossed polarizers) of A) commercial polyethylene (HDPE) sheet, B) polypropylene film prepared by spin coating for SMFM experiments. C) Plot shows pull-off force histograms for MCTS 1 on polyethylene surfaces in (blue histogram) aqueous PBS containing 60 v% methanol (blue histogram) and (green histogram) aqueous PBS following the

measurements in the presence of methanol. The force histogram in purple was obtained using MCTS 1 in PBS buffer prior to exposure to methanol, and is reproduced from Figure 3C for comparison.

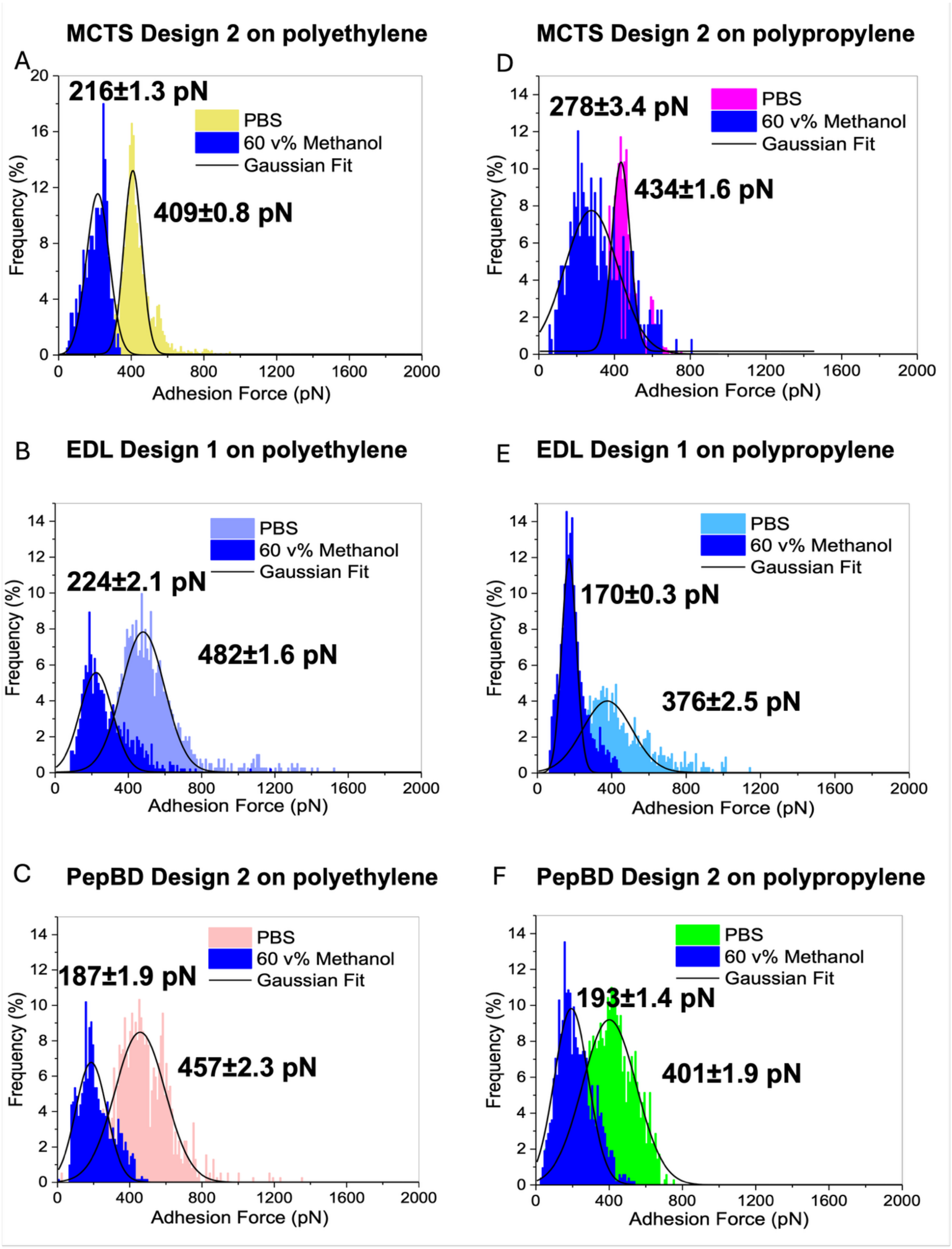
MCTS Design 2 on polyethylene
A
216±1.3 pN
409±0.8 pN
PBS
60 v% Methanol
Gaussian Fit
Frequency (%)
Adhesion Force (pN)
MCTS Design 2 on polypropylene
D
278±3.4 pN
434±1.6 pN
PBS
60 v% Methanol
Gaussian Fit
Frequency (%)
Adhesion Force (pN)
B
EDL Design 1 on polyethylene
224±2.1 pN
482±1.6 pN
PBS
60 v% Methanol
Gaussian Fit
Frequency (%)
Adhesion Force (pN)
E
EDL Design 1 on polypropylene
170±0.3 pN
376±2.5 pN
PBS
60 v% Methanol
Gaussian Fit
Frequency (%)
Adhesion Force (pN)
C
PepBD Design 2 on polyethylene
187±1.9 pN
457±2.3 pN
PBS
60 v% Methanol
Gaussian Fit
Frequency (%)
Adhesion Force (pN)
F
PepBD Design 2 on polypropylene
193±1.4 pN
401±1.9 pN
PBS
60 v% Methanol
Gaussian Fit
Frequency (%)
Adhesion Force (pN)

Figure S13. Comparison of $F_{pull-off}$ distributions for both polyethylene and polypropylene for peptides in aqueous PBS or 60% methanol added to aqueous PBS. Each plot shows the $F_{pull-off}$ distribution from 1500 measurements with either polyethylene and polypropylene, in either aqueous PBS or a 60% methanol/aqueous PBS. Each plot provides the mean and standard deviation from fitting a Gaussian distribution to the experimental data.

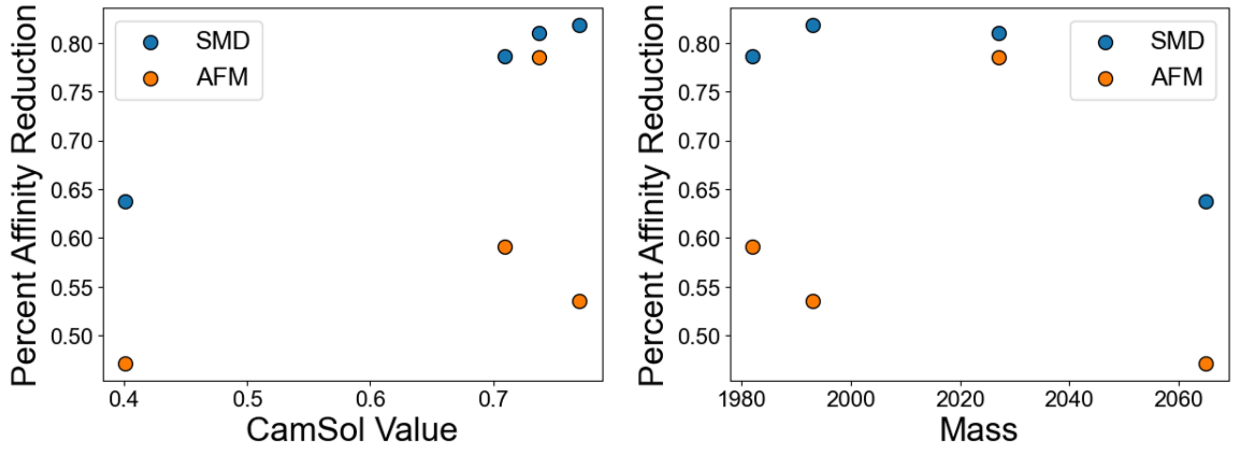

Figure S14. Correlation between peptide CamSol score (predicted aqueous solubility) and mass versus the reduction in peptide affinity in 60% methanol relative to water. The reduction in affinity corresponds to the data shown in Figure 7. For the CamSol score, a higher value indicates lower hydrophobicity and greater solubility in water.

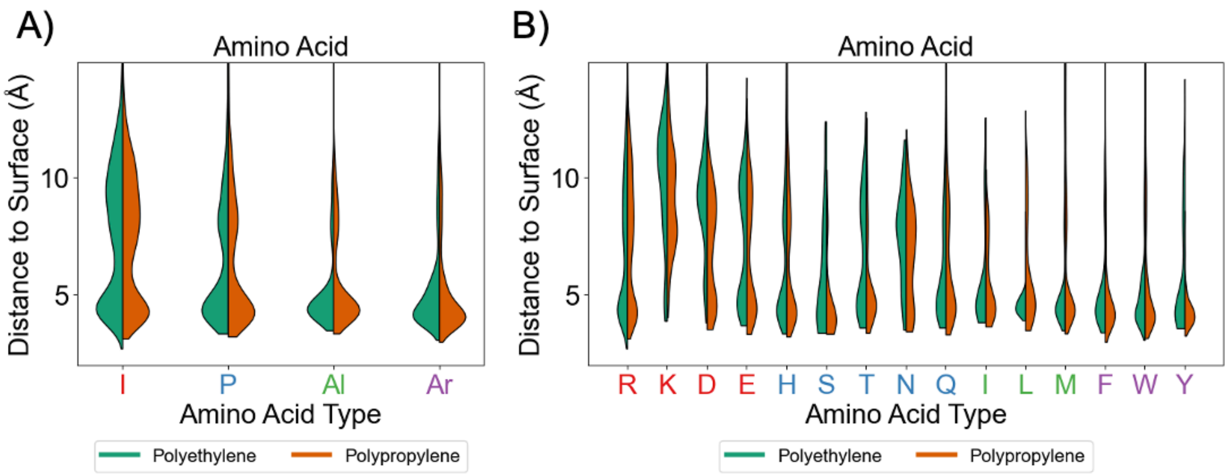

Figure S15. Distribution of distances between amino acid side-chain (center-of-mass) and either polyethylene or polypropylene surfaces. Each panel shows the distribution of distances for each amino acid side chain center of mass and the plastic surface for equilibrated adsorbed conformations obtained in SMD simulations for all 10 designed peptides (Table 1). Results are grouped by A) amino acid side-chain category, where I is ionic (arginine, lysine, aspartic acid, and glutamic acid), P is polar (asparagine, glutamine, glycine, histidine, proline, serine, and threonine), Al is aliphatic (alanine, cysteine, isoleucine, leucine, methionine, and valine), and Ar is aromatic (phenylalanine, tryptophan, and tyrosine), and B) amino acid. Color coding of amino acid letters in B) aligns with amino acid category in A).

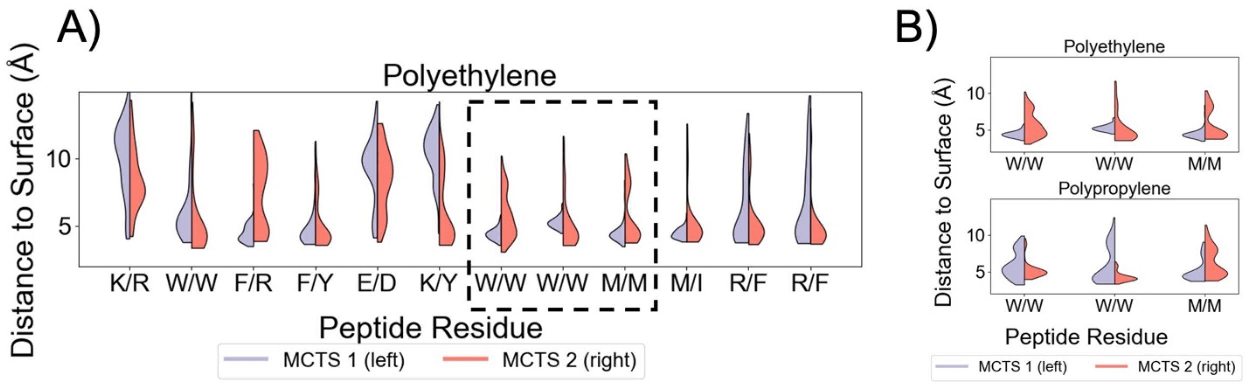

Figure S16. Interaction of MCTS 1 and MCTS 2 with polyethylene. Each panel shows the distribution of distances between each amino acid side chain center of mass and the plastic surface for equilibrated adsorbed conformations obtained in SMD simulations. The distance distributions for each residue are shown in purple for MCTS 1 and pink for MCTS 2. A) Comparison of MCTS 1 and 2 on polyethylene. B) Comparison of the WWM residues (the boxed residues in panel A) for MCTS 1 and MCTS 2 on polyethylene and polypropylene. Note that MCTS 1 shows stronger adhesion to polyethylene than MCTS 2. Data from Figure 8 is partially reproduced in this figure for completeness and ease of reading of the SI.

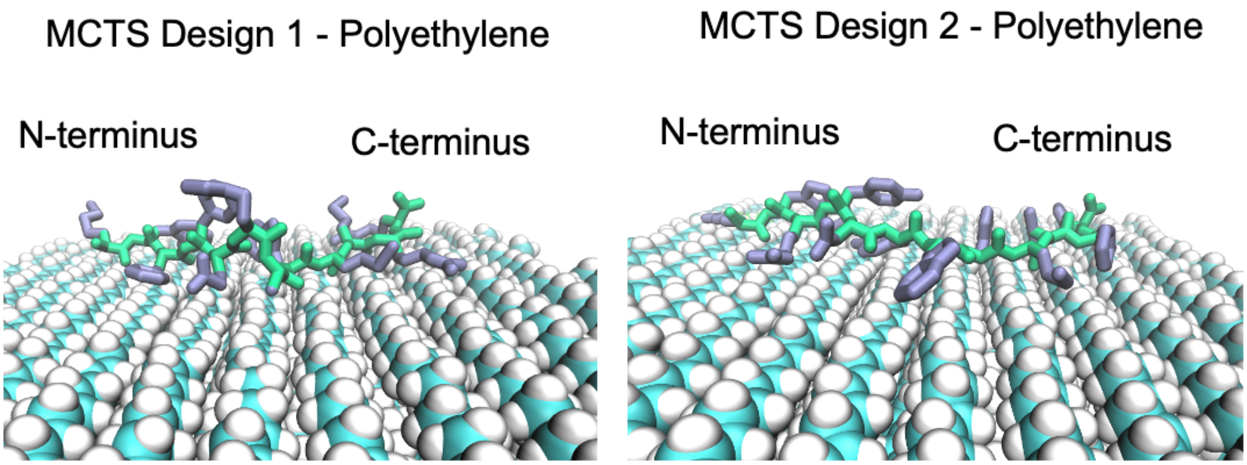

Figure S17. Example adsorbed structures of MCTS Peptides 1 and 2 on polyethylene. Polyethylene shown as blue van der Waals spheres, and peptide is shown as licorice with backbone in green and side chains in blue. Hydrogens in the peptide are hidden for clarity. Peptides are oriented so the N-terminus is on the left and C-terminus is on the right, so the structure can be compared with Figure 8. Figure made with VMD.

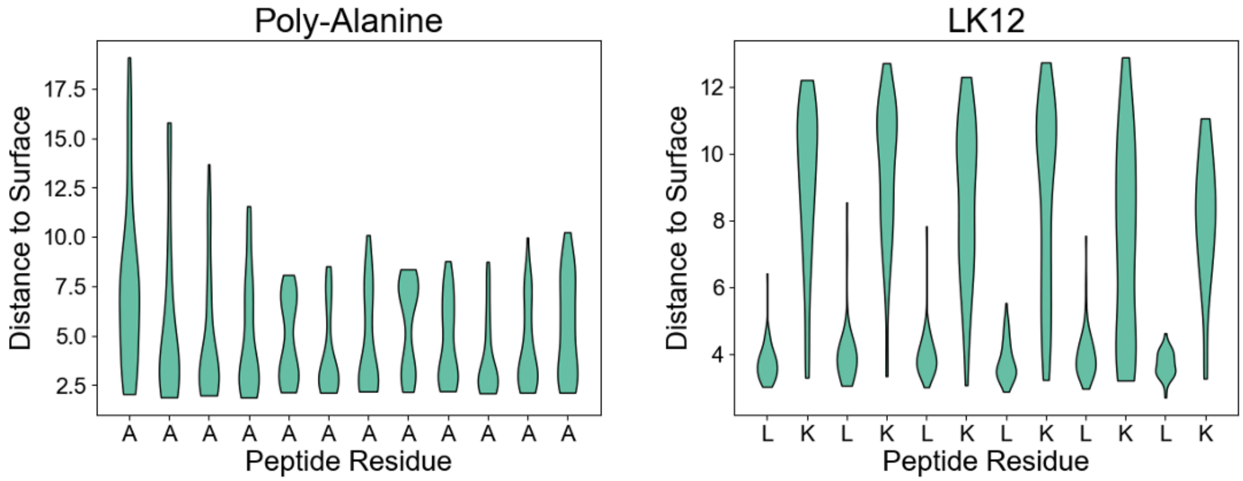

Figure S18. Adsorbed conformations for controls LK12 and Poly-Alanine to polyethylene. Results are averaged over the 48 equilibrated structures prior to beginning the production simulation of SMD. Data for each amino acid is visualized as a violin plot.

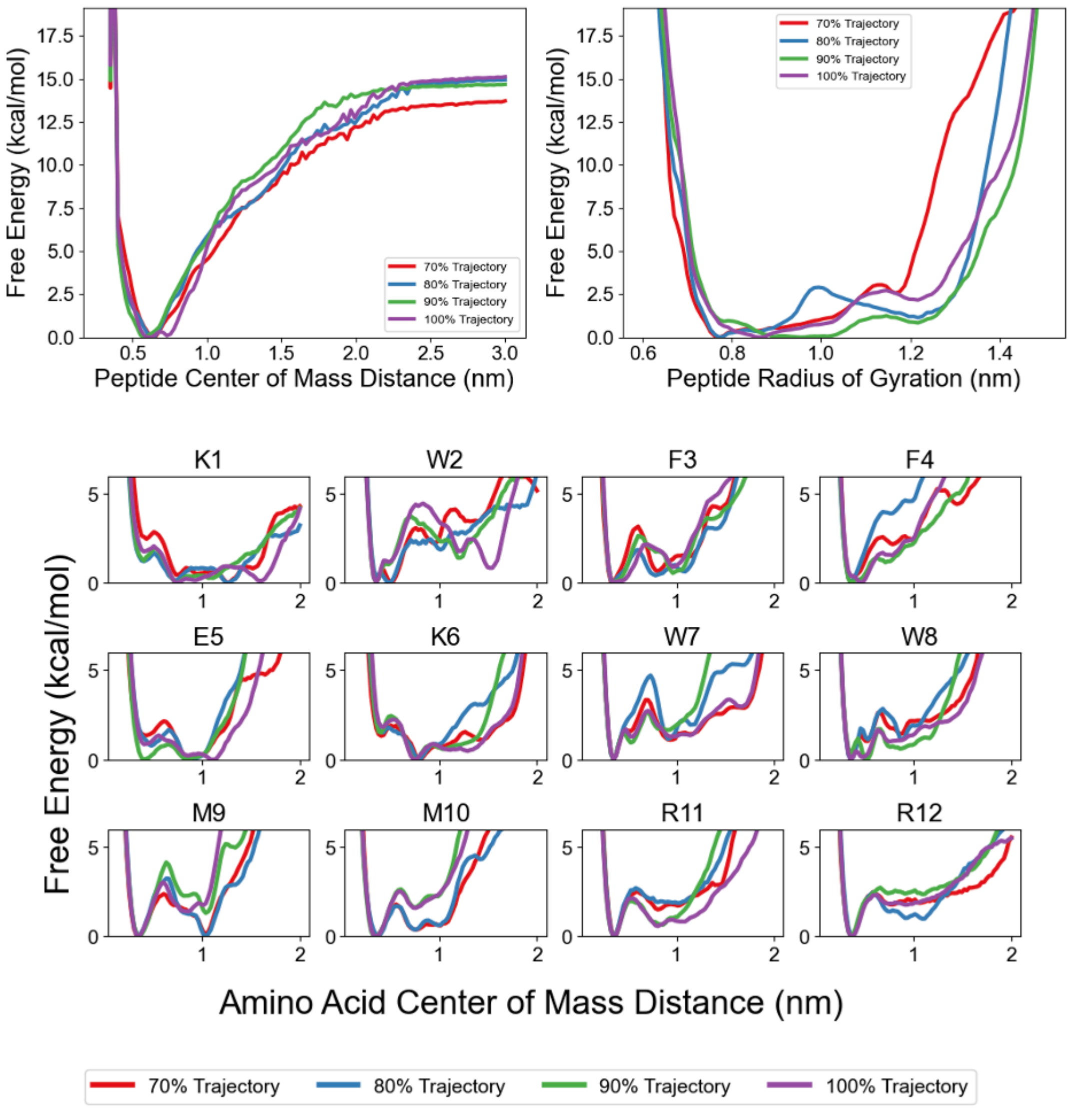

Figure S19. Convergence of free energy profiles for MCTS Design 1 in PBMetaD simulations. Legend shows the fraction of the simulation of each of the four walkers used to construct the free energy profile. The top-left panel shows the convergence of the free energy profile as a function of the peptide radius of gyration. The top-right panel shows the convergence of the free energy profile as a function of the distance between the peptide center of mass and the polyethylene surface. The bottom panel shows the convergence of the free energy profile as a function of the distance between each amino acid's side chain center of mass and the top of the polyethylene surface.

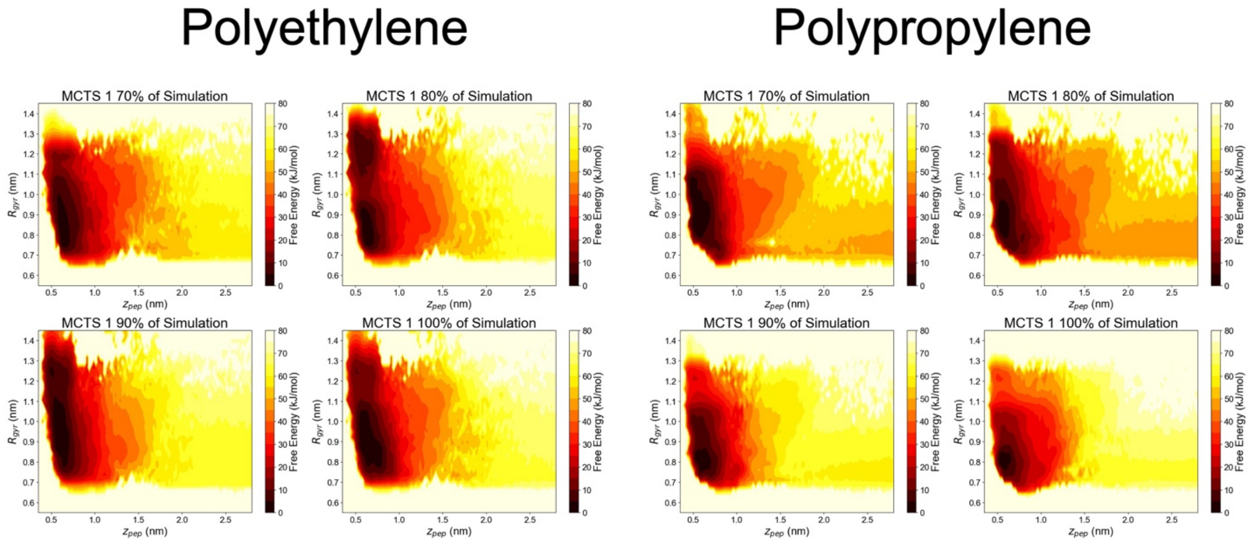

Figure S20. Convergence of two-dimensional free energy surfaces for MCTS Design 1. Plots shown for the peptide adsorbing to polyethylene (left) and polypropylene (right). Free energy surfaces shown as a contour plot for 70, 80, 90, and 100% of the entire metadynamics simulation.

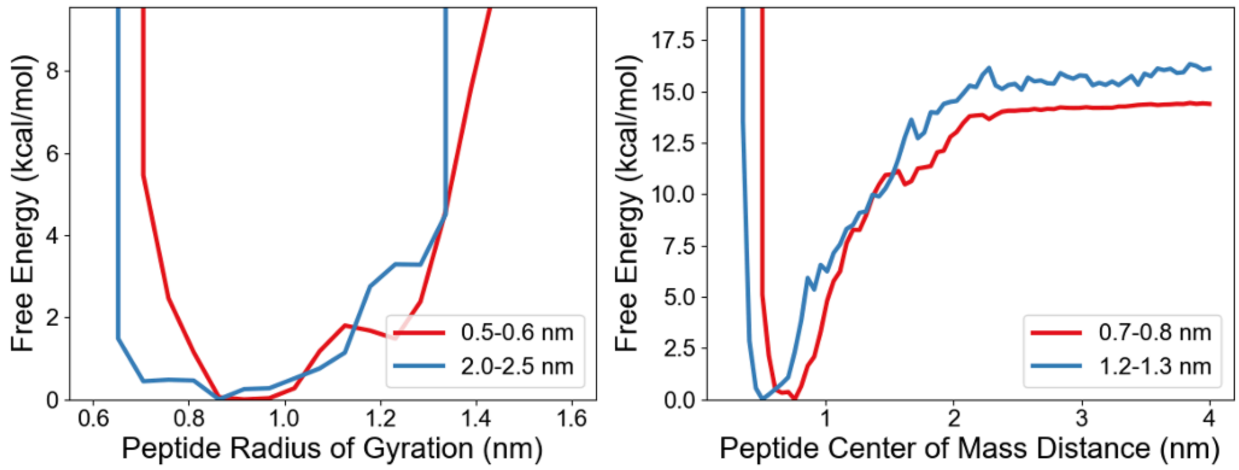

Figure S21. One-dimensional free energy profiles of MCTS Design 1 at fixed peptide radius of gyration or distance to polyethylene surface. Left plot shows the free energy profile for the peptide radius of gyration for certain distance intervals between the peptide center of mass and the polyethylene surface. Right plot shows the free energy profile as a function of this distance for fixed intervals of the peptide radius of gyration. The legend provides the range of values for the fixed variable. Free energy profiles obtained using the normal reweighting procedure, where only data points in the range of values for the fixed variable were included.

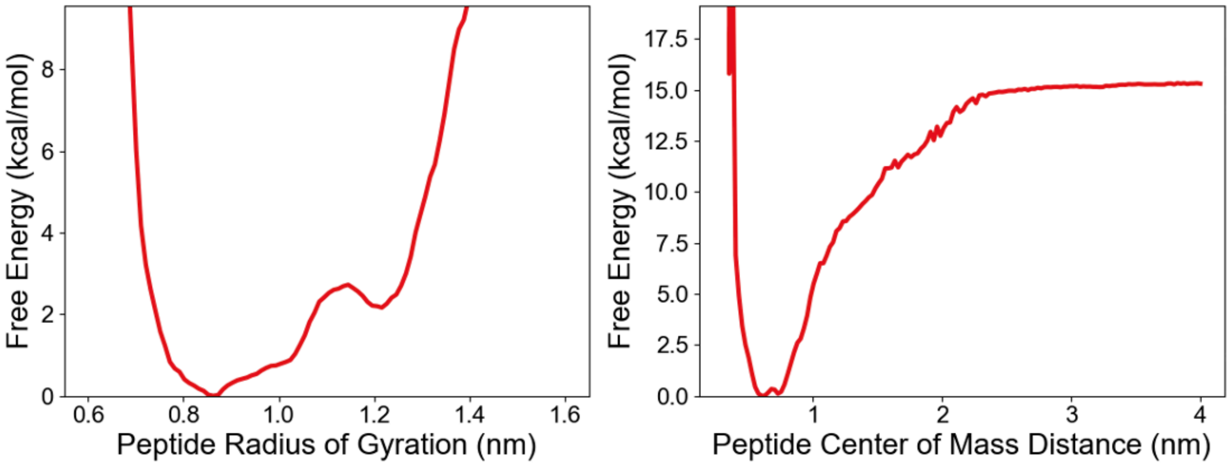

Figure S22. One-dimensional free energy profiles for MCTS Design 1 adsorbing to polyethylene. Free energy profiles are provided as a function of the peptide radius of gyration (left) and the distance between the peptide's center of mass and the top of the polyethylene surface (right). These plots are obtained via marginalization of the two-dimensional free energy surface in Figure 9.

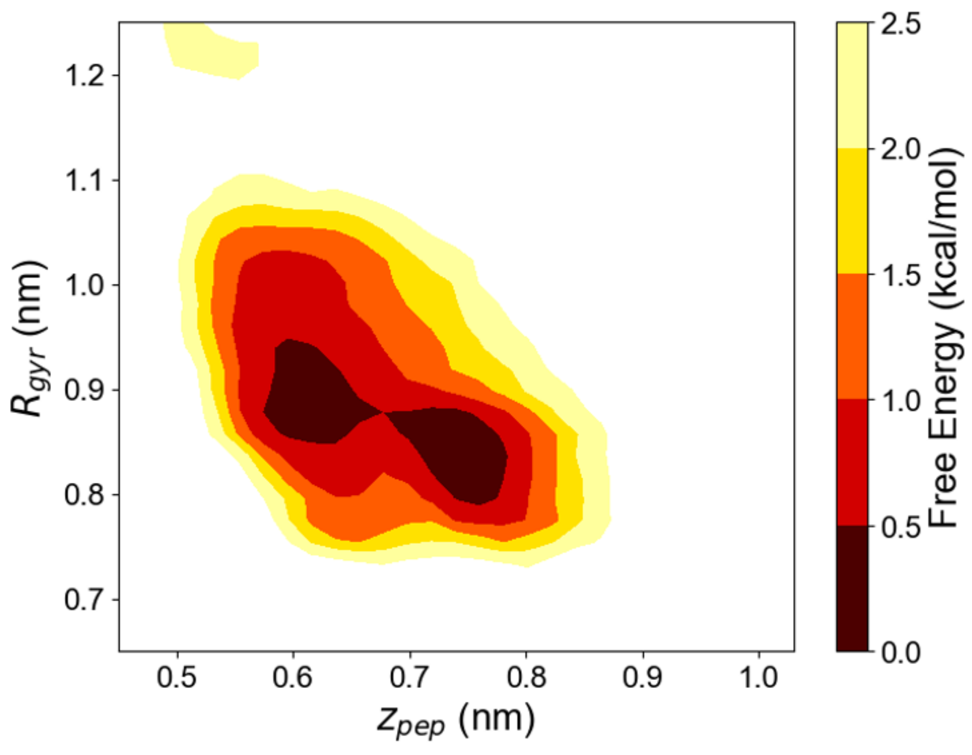

Figure S23. Two-dimensional free energy surface of MCTS Design 1 in the global free energy minimum. This figure provides a magnified view of the global minimum in the two-dimensional free energy profile shown in Figure 9.

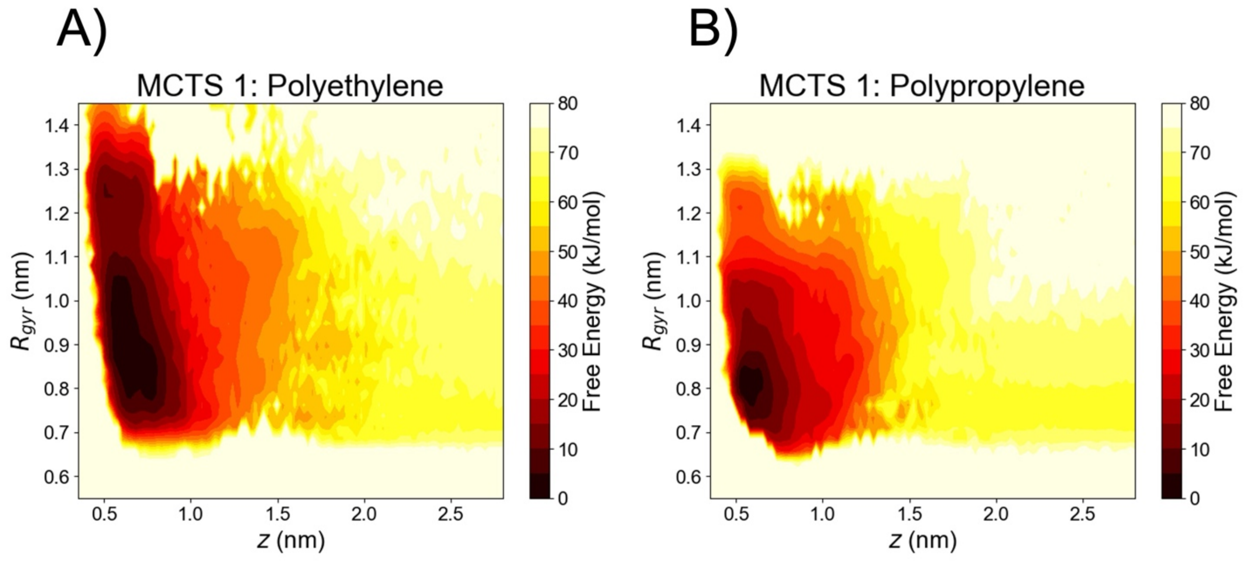

Figure S24. Comparison of MCTS 1 adsorption to polyethylene and polypropylene. A/B) two-dimensional free energy surfaces, in terms of R_{gyr} and the peptide center of mass, for peptide adsorption to polyethylene/polypropylene. Panel A is identical to that of Figure 9A in the main text.

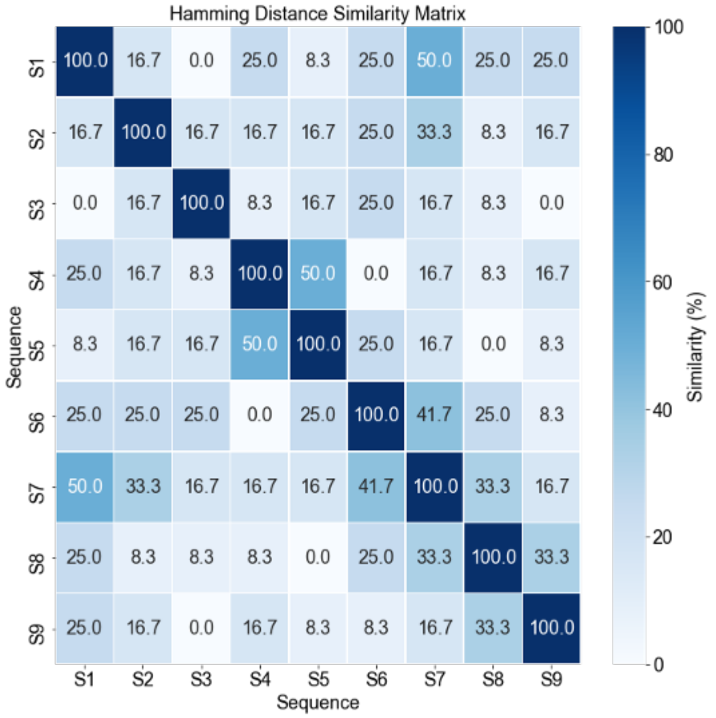

Figure S25. Hamming similarity matrix to evaluate the similarity of the selected peptide sequences from different methodologies. Pairwise Hamming similarity scores (%) between all nine peptide sequences (S1-S9, description in the table below). Hamming similarity is calculated as the percentage of positions where amino acids match exactly between two aligned sequences, with 100% indicating identical sequences and 0% indicating no positional matches. The color intensity (darker blue indicates higher similarity) reflects the degree of sequence conservation at specific positions. Diagonal elements show 100% similarity (self-comparison). The matrix reveals limited sequence similarity across the designed peptides, with most pairwise comparisons showing less than 25% (average 19%) positional conservation, indicating substantial sequence diversity despite comparable binding performance to polyethylene surfaces.

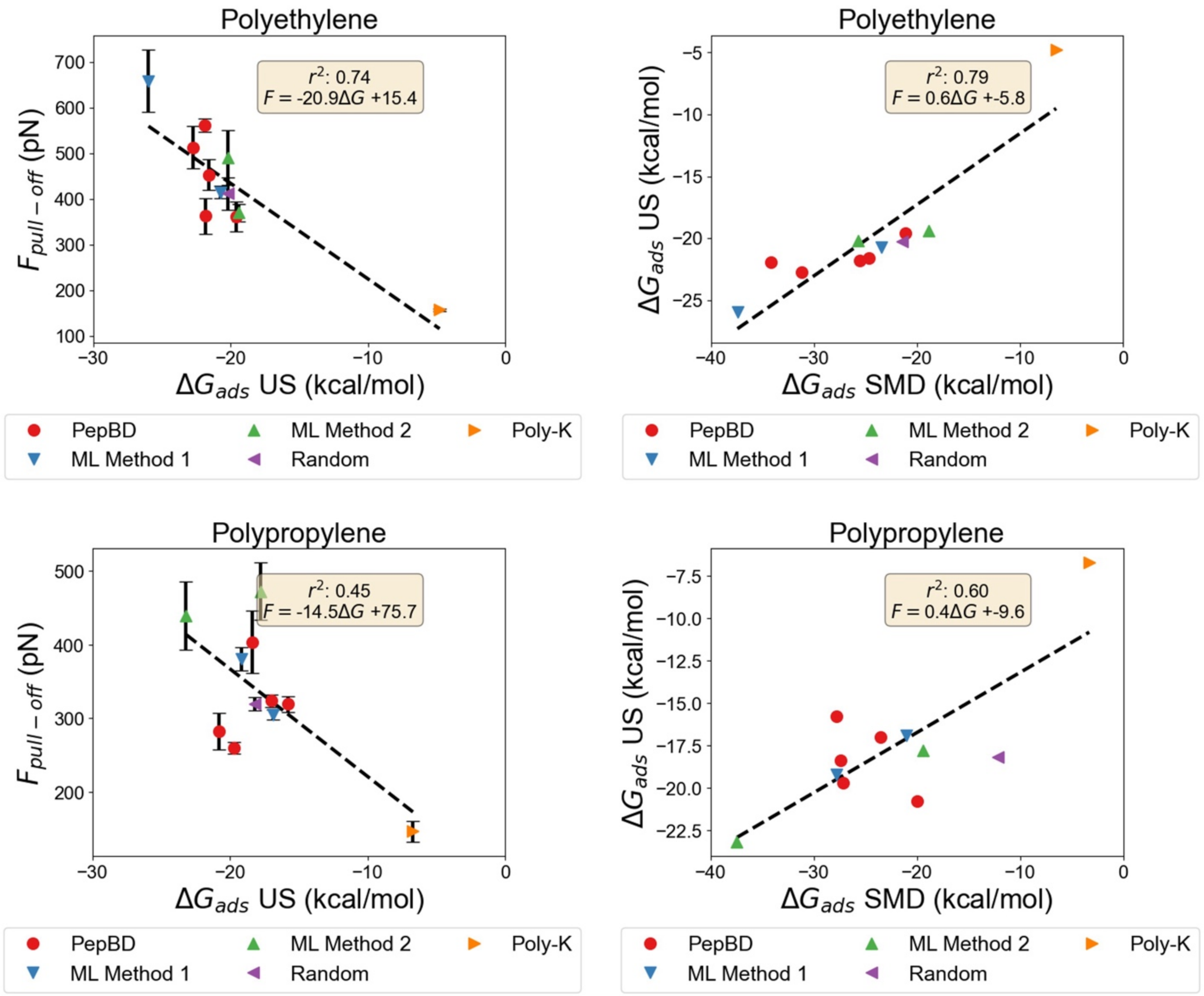

Figure S26. Correlation between Umbrella Sampling (US) and either SMD or SMFM. Top/bottom row shows the results for polyethylene/polypropylene, while the left/right column compares the correlation between SMFM/SMD results and US results**.** Text boxes show the best fit line from linear regression along with the r^2, the squared Pearson correlation coefficient. Error bars for $F_{pull\text{-}off}$ are 1 standard error of mean (SEM) from 3 replicate measurements.

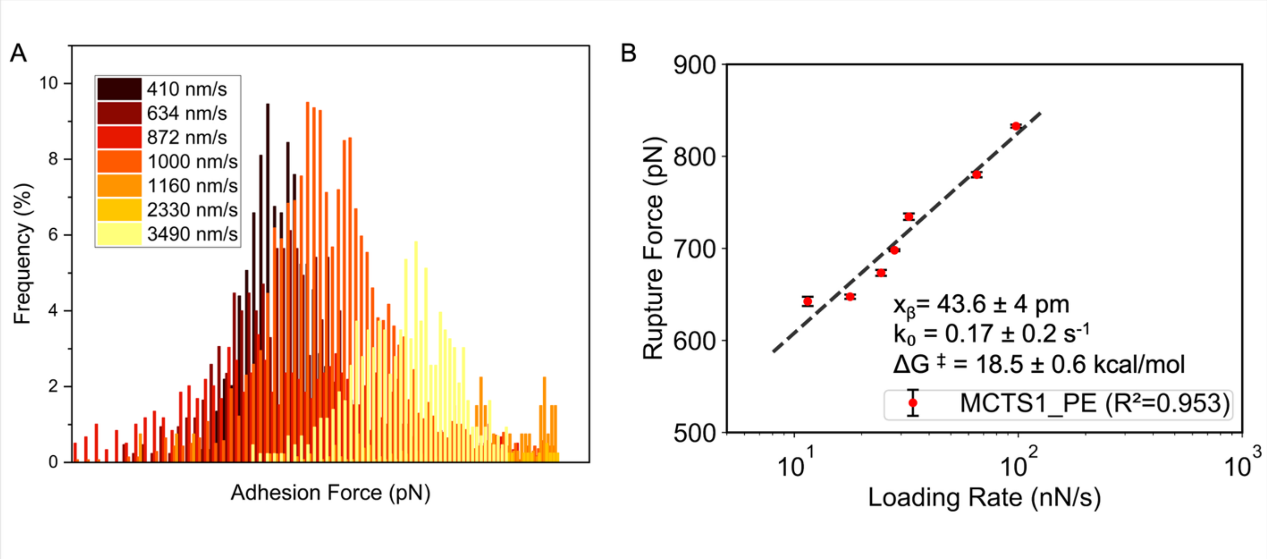

Figure S27. Dynamic force spectroscopy of MCTS 1 on polyethylene. A. Overlay of pull-off force histograms for MCTS 1 on polyethylene surface for retraction speeds of 410, 634, 872, 1000, 1160, 2330 and 3490 nm/s, with other parameters kept constant. The measurements were performed in aqueous PBS. B. Bell Evans model fitting on rupture force vs loading rate data for MCTS 1 sequence on polyethylene surface. This was used to extract kinetic parameters (barrier height, x_β, dissociation rate, k_0 and free energy for bond dissociation, $\Delta G^\ddagger$) reported in the legend.

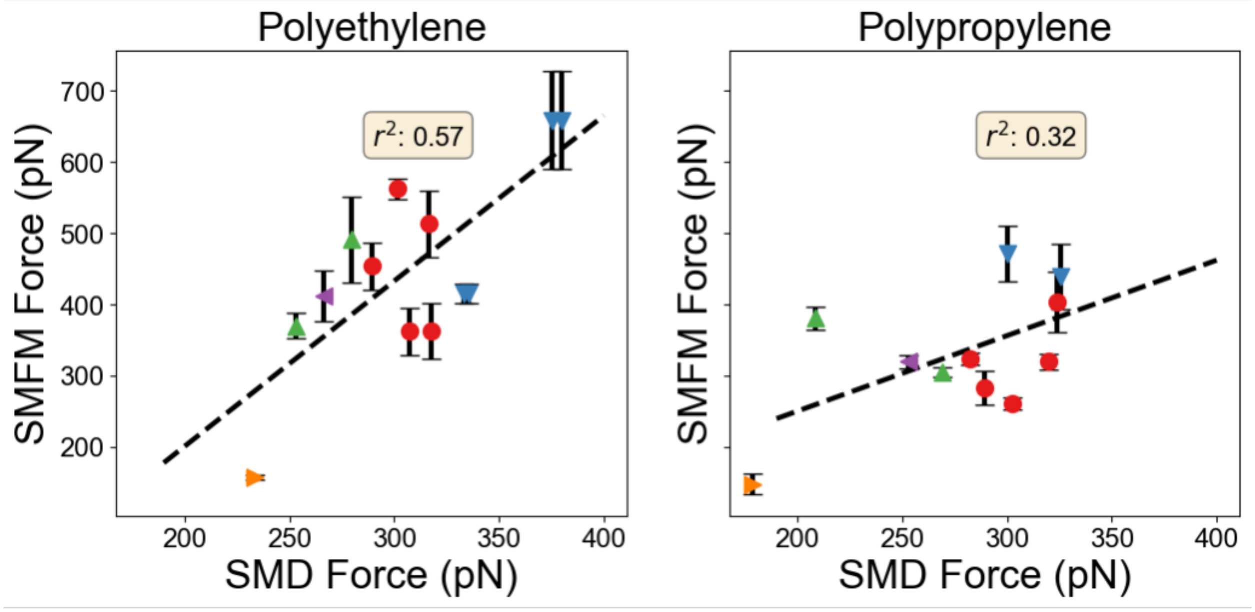

Figure S28. Correlation between pull-off force in SMD simulations and SMFM. r^2 Pearson correlation coefficients are obtained using linear least-squares regression.

Table S1. Change in ΔG_{ads}^{SMD} upon doubling the number of simulations for polyethylene.

Peptide	ΔG_{ads}^{SMD}, 48 Simulations (kcal/mol)	ΔG_{ads}^{SMD}, 96 Simulations (kcal/mol)
MCTS Design 1	-37.5	-35.5
MCTS Design 2	-23.5	-23.9

Table S2. Comparison of ΔG_{ads}^{SMD} upon repeating the entire SMD procedure for polyethylene and polypropylene.

Peptide	ΔG_{ads}^{SMD} Polyethylene, Run 1 (kcal/mol)	ΔG_{ads}^{SMD} Polyethylene, Run 2 (kcal/mol)	ΔG_{ads}^{SMD} Polypropylene, Run 1 (kcal/mol)	ΔG_{ads}^{SMD} Polypropylene, Run 2 (kcal/mol)
MCTS Design 1	-37.0	-37.5	-19.4	-25.1
MCTS Design 2	-23.5	-29.9	-37.4	-33.7
Random 1	-17.6	-22.2	-15.7	-14.3
Random 2	-23.1	-21.9	-22.1	-17.6

Table S3. Summary of SMFM experimental parameters[1]

Parameter	Value
Retraction velocity	1000 nm/s
Approach velocity	1000 nm/s
Loading rate	28 ± 8 nN/s
Cantilever spring constant (nominal)	0.01 N/m
Cantilever spring constant (effective)	0.028 ± 0.008 N/m
Calibration method	Thermal tuning (Asylum MFP-3D)
Contact time	500 ms
Cross-linker (tether)	Sulfosuccinimidyl-4-(N-maleimidomethyl)cyclohexane-1-carboxylate (SSMCC)
Tether length	~1 nm
Cantilever type	Triangular Si_3N_4 (Bruker MSCT-UC)
Aqueous Phase	PBS, room temperature or 60 v% methanol in PBS
Instrument	Nanoscope IIIa Multimode AFM with fluid cell

[1]All parameters were held constant across all peptide sequences tested.

Table S4. Summary of SMFM adhesion events measured across three independent trials

	Polyethylene (0.1%)		Polypropylene (0.1%)	
**Peptide**	**Adhesion Events/Independent Trials**	**Excluded Curves**	**Adhesion Events**	**Excluded Curves**
PepBD 1	1361/3	3	1148/3	3
PepBD 2	1297/3	1	1823/3	6
PepBD 3	2140/3	0	1483/3	2
PepBD 5	1408/3	6	1504/3	11
PepBD 6	1189/3	3	1606/3	0
EDL 1	1172/3	2	1554/3	4
EDL 2	2764/3	13	1386/3	9
MCTS 1	1655/3	0	1730/3	0
MCTS 2	2036/3	9	1526/3	1
Polyglutamic Acid	1629/3	12	2127/3	15
Random Sequence 5	1856/3	8	2058/3	4

Peptide concentration of 0.1%. Excluded curves indicates the number of force curves excluded for showing more than one peak >10 pN.

Table S5. Affinity of peptides to polyethylene (PE) and polypropylene (PP) using all computational methods.[1]

Description	Sequence	ΔG PE, MM/GBSA (kcal/mol)	ΔG PE, SMD (kcal/mol)	ΔG PP, SMD (kcal/mol)	ΔG PE, Umbrella (kcal/mol)	ΔG PP, Umbrella (kcal/mol)
PepBD 1	HWMRTWRWHMFH	-36.8	-21.1	-27.8	-19.6	-15.8
PepBD 2	RWMWHMHRQTMW	-27.7	-24.7	-27.4	-21.6	-18.4
PepBD 3	MFWWRELHQQWR	-27.0	-34.2	-23.5	-21.9	-17.0
PepBD 5	HRWMMDHWRSFW	-25.8	-31.2	-20.0	-22.8	-20.8
PepBD 6	HRWWWDHFRLHF	-25.4	-25.5	-27.2	-21.8	-19.7
EDL 1	DWMWRREFFMHR	-25.4	-25.7	-27.8	-20.2	-19.2
EDL 2	HWMWRMKWNMRH	-30.3	-18.9	-21.0	-19.4	-16.9
MCTS 1	KWFFEKWWMMRR	-37.0	-37.5	-19.4	-26.0	-17.8
MCTS 2	RWRYDYWWMIFF	-28.9	-23.5	-37.4	-20.7	-23.2
Control 3	LKLKLKLKLKLK		-16.7	-13.8	-4.8	-6.7
Random 5	WTYRMTASMRNF	-15.1	-21.5	-12.1	-20.2	-18.2

[1]If entry is blank, then affinity was not measured using the method. Affinity of peptides for polypropylene was not measured using MM/GBSA.

Table S6. Average and best ΔG_{ads}^{SMD} and $F_{pull\text{-}off}$ for peptides to polypropylene.[1]

Peptide Category	ΔG_{ads}^{SMD} (± 5.7 kcal/mol) Average / Best	$F_{pull\text{-}off}$ (pN) Average / Best
PepBD	-23.6 / -27.8	318 ± 22 / 403 ± 34
MCTS	**-28.4 / -37.4**	**455** ± 12/ **472** ± 32
EDL	-24.4 / -27.8	343 ± 27 / 380 ± 13
Controls	-8.1 / -13.8	147 ± 12
Random	-14.4 / -22.1	319 ± 8

[1]Bold entries indicate the peptides with the highest affinity for polypropylene using SMFM or SMD. The $F_{pull\text{-}off}$ error values indicate standard error of mean (SEM).

Table S7. Peptides designed by PepBD to bind to polypropylene have higher affinity for polyethylene.

Description	Sequence	$\Delta\Delta G_{ads}^{SMD}$ (± 11.0 kcal/mol) Polyethylene – Polypropylene
PepBD Design 1	WWQRHEFFWRTF	-8.4
PepBD Design 2	WWQRHEFEFRHW	-4.5
PepBD Design 3	WMWHEMHTWRFR	-14.7
PepBD Design 4	WFQRHQFHWRIW	-11.6

Table S8. Comparison of peptide affinity to polyethylene and polypropylene in water or 60% Methanol in SMD simulations

Description	Sequence	Polyethylene		Polypropylene	
		ΔG_{ads}^{SMD} Water (kcal/mol)	ΔG_{ads}^{SMD} Methanol (kcal/mol)	ΔG_{ads}^{SMD} Water (kcal/mol)	ΔG_{ads}^{SMD} Methanol (kcal/mol)
PepBD Design 2	RWMWHMHRQTMW	-24.68	-5.19	-24.68	-5.19
EDL Design 1	DWMWRREFFMHR	-25.72	-4.51	-25.72	-4.51
MCTS Design 1	KWFFEKWWMMRR	-37.45	-6.54	-37.45	-6.54
MCTS Design 2	RWRYDYWWMIFF	-23.49	-9.71	-23.49	-9.71

References

(1) Alshehri, A. S.; Bergman, M. T.; You, F.; Hall, C. K. Biophysics-Guided Uncertainty-Aware Deep Learning Uncovers High-Affinity Plastic-Binding Peptides. *Digital Discovery* **2025**, *4* (2), 561–571. https://doi.org/10.1039/D4DD00219A.

(2) Wang, Y.; Stebe, K. J.; de la Fuente-Nunez, C.; Radhakrishnan, R. Computational Design of Peptides for Biomaterials Applications. *ACS Appl. Bio Mater.* **2024**, *7* (2), 617–625. https://doi.org/10.1021/acsabm.2c01023.

(3) Vanhee, P.; van der Sloot, A. M.; Verschueren, E.; Serrano, L.; Rousseau, F.; Schymkowitz, J. Computational Design of Peptide Ligands. *Trends in Biotechnology* **2011**, *29* (5), 231–239. https://doi.org/10.1016/j.tibtech.2011.01.004.

(4) Hosseinzadeh, P.; Bhardwaj, G.; Mulligan, V. K.; Shortridge, M. D.; Craven, T. W.; Pardo-Avila, F.; Rettie, S. A.; Kim, D. E.; Silva, D.-A.; Ibrahim, Y. M.; Webb, I. K.; Cort, J. R.; Adkins, J. N.; Varani, G.; Baker, D. Comprehensive Computational Design of Ordered Peptide Macrocycles. *Science* **2017**, *358* (6369), 1461–1466. https://doi.org/10.1126/science.aap7577.

(5) Huang, P.-S.; Boyken, S. E.; Baker, D. The Coming of Age of de Novo Protein Design. *Nature* **2016**, *537* (7620), 320–327. https://doi.org/10.1038/nature19946.

(6) Wang, E.; Sun, H.; Wang, J.; Wang, Z.; Liu, H.; Zhang, J. Z. H.; Hou, T. End-Point Binding Free Energy Calculation with MM/PBSA and MM/GBSA: Strategies and Applications in Drug Design. *Chem. Rev.* **2019**, *119* (16), 9478–9508. https://doi.org/10.1021/acs.chemrev.9b00055.

(7) Sarma, S.; Catella, C. M.; San Pedro, E. T.; Xiao, X.; Durmusoglu, D.; Menegatti, S.; Crook, N.; Magness, S. T.; Hall, C. K. Design of 8-Mer Peptides That Block Clostridioides Difficile Toxin A in Intestinal Cells. *Commun Biol* **2023**, *6* (1), 1–13. https://doi.org/10.1038/s42003-023-05242-x.

(8) Catella, C. M.; Sarma, S.; Hinesley, C. M.; Febo, C. E.; Breau, K. A.; Durmusoglu, D.; Purnell, E.; Magness, S. T.; Hall, C. K.; Menegatti, S.; Crook, N. Development of Peptide Glucosyltransferase Inhibitors With Comprehensive Coverage Across Clostridioides Difficile Toxin B Sub-Types. *Biotechnology and Bioengineering* **2026**, *123* (2), 310–323. https://doi.org/10.1002/bit.70102.

(9) Xiao, X.; Kuang, Z.; Slocik, J. M.; Tadepalli, S.; Brothers, M.; Kim, S.; Mirau, P. A.; Butkus, C.; Farmer, B. L.; Singamaneni, S.; Hall, C. K.; Naik, R. R. Advancing Peptide-Based Biorecognition Elements for Biosensors Using in-Silico Evolution. *ACS Sens.* **2018**, *3* (5), 1024–1031. https://doi.org/10.1021/acssensors.8b00159.

(10) Xiao, X.; Kilgore, R.; Sarma, S.; Chu, W.; Menegatti, S.; Hall, C. K. De Novo Discovery of Peptide-Based Affinity Ligands for the Fab Fragment of Human Immunoglobulin G. *Journal of Chromatography A* **2022**, *1669*, 462941. https://doi.org/10.1016/j.chroma.2022.462941.

(11) Welch, C. M.; Camden, A. N.; Barr, S. A.; Leuty, G. M.; Kedziora, G. S.; Berry, R. J. Computation of the Binding Free Energy of Peptides to Graphene in Explicit Water. *J. Chem. Phys.* **2015**, *143* (4), 045104. https://doi.org/10.1063/1.4927344.

(12) Case, D. A.; Cheatham, T. E.; Darden, T.; Gohlke, H.; Luo, R.; Merz, K. M.; Onufriev, A.; Simmerling, C.; Wang, B.; Woods, R. J. The Amber Biomolecular Simulation Programs. *J Comput Chem* **2005**, *26* (16), 1668–1688. https://doi.org/10.1002/jcc.20290.

(13) Case, D. A.; Aktulga, H. M.; Belfon, K.; Cerutti, D. S.; Cisneros, G. A.; Cruzeiro, V. W. D.; Forouzesh, N.; Giese, T. J.; Götz, A. W.; Gohlke, H.; Izadi, S.; Kasavajhala, K.;

Kaymak, M. C.; King, E.; Kurtzman, T.; Lee, T.-S.; Li, P.; Liu, J.; Luchko, T.; Luo, R.; Manathunga, M.; Machado, M. R.; Nguyen, H. M.; O'Hearn, K. A.; Onufriev, A. V.; Pan, F.; Pantano, S.; Qi, R.; Rahnamoun, A.; Risheh, A.; Schott-Verdugo, S.; Shajan, A.; Swails, J.; Wang, J.; Wei, H.; Wu, X.; Wu, Y.; Zhang, S.; Zhao, S.; Zhu, Q.; Cheatham, T. E. I.; Roe, D. R.; Roitberg, A.; Simmerling, C.; York, D. M.; Nagan, M. C.; Merz, K. M. Jr. AmberTools. *J. Chem. Inf. Model.* **2023**, *63* (20), 6183–6191. https://doi.org/10.1021/acs.jcim.3c01153.

(14) Abraham, M. J.; Murtola, T.; Schulz, R.; Páll, S.; Smith, J. C.; Hess, B.; Lindahl, E. GROMACS: High Performance Molecular Simulations through Multi-Level Parallelism from Laptops to Supercomputers. *SoftwareX* **2015**, *1–2*, 19–25. https://doi.org/10.1016/j.softx.2015.06.001.

(15) Hess, B.; Bekker, H.; Berendsen, H. J. C.; Fraaije, J. G. E. M. LINCS: A Linear Constraint Solver for Molecular Simulations. *Journal of Computational Chemistry* **1997**, *18* (12), 1463–1472. https://doi.org/10.1002/(SICI)1096-987X(199709)18:12%3C1463::AID-JCC4%3E3.0.CO;2-H.

(16) Essmann, U.; Perera, L.; Berkowitz, M. L.; Darden, T.; Lee, H.; Pedersen, L. G. A Smooth Particle Mesh Ewald Method. *The Journal of Chemical Physics* **1995**, *103* (19), 8577–8593. https://doi.org/10.1063/1.470117.

(17) Bussi, G.; Donadio, D.; Parrinello, M. Canonical Sampling through Velocity Rescaling. *J Chem Phys* **2007**, *126* (1), 014101. https://doi.org/10.1063/1.2408420.

(18) Berendsen, H. J. C.; Postma, J. P. M.; van Gunsteren, W. F.; DiNola, A.; Haak, J. R. Molecular Dynamics with Coupling to an External Bath. *J. Chem. Phys.* **1984**, *81* (8), 3684–3690. https://doi.org/10.1063/1.448118.

(19) Jorgensen, W. L. Quantum and Statistical Mechanical Studies of Liquids. 10. Transferable Intermolecular Potential Functions for Water, Alcohols, and Ethers. Application to Liquid Water. *J. Am. Chem. Soc.* **1981**, *103* (2), 335–340. https://doi.org/10.1021/ja00392a016.

(20) Vassetti, D.; Pagliai, M.; Procacci, P. Assessment of GAFF2 and OPLS-AA General Force Fields in Combination with the Water Models TIP3P, SPCE, and OPC3 for the Solvation Free Energy of Druglike Organic Molecules. *J. Chem. Theory Comput.* **2019**, *15* (3), 1983–1995. https://doi.org/10.1021/acs.jctc.8b01039.

(21) Bergman, M. T.; Xiao, X.; Hall, C. K. In Silico Design and Analysis of Plastic-Binding Peptides. *J. Phys. Chem. B* **2023**, *127* (39), 8370–8381. https://doi.org/10.1021/acs.jpcb.3c04319.

(22) Maier, J. A.; Martinez, C.; Kasavajhala, K.; Wickstrom, L.; Hauser, K. E.; Simmerling, C. ff14SB: Improving the Accuracy of Protein Side Chain and Backbone Parameters from ff99SB. *J. Chem. Theory Comput.* **2015**, *11* (8), 3696–3713. https://doi.org/10.1021/acs.jctc.5b00255.

(23) Vanquelef, E.; Simon, S.; Marquant, G.; Garcia, E.; Klimerak, G.; Delepine, J. C.; Cieplak, P.; Dupradeau, F.-Y. R.E.D. Server: A Web Service for Deriving RESP and ESP Charges and Building Force Field Libraries for New Molecules and Molecular Fragments. *Nucleic Acids Res* **2011**, *39* (Web Server issue), W511–W517. https://doi.org/10.1093/nar/gkr288.

(24) Vendrell, R. C.; Ajagekar, A.; Bergman, M. T.; Hall, C. K.; You, F. Designing Microplastic-Binding Peptides with a Variational Quantum Circuit–Based Hybrid Quantum-Classical Approach. *Science Advances* **2024**, *10* (51), eadq8492. https://doi.org/10.1126/sciadv.adq8492.

(25) Humphrey, W.; Dalke, A.; Schulten, K. VMD: Visual Molecular Dynamics. *Journal of Molecular Graphics* **1996**, *14* (1), 33–38. https://doi.org/10.1016/0263-7855(96)00018-5.
(26) Tribello, G. A.; Bonomi, M.; Branduardi, D.; Camilloni, C.; Bussi, G. PLUMED 2: New Feathers for an Old Bird. *Computer Physics Communications* **2014**, *185* (2), 604–613. https://doi.org/10.1016/j.cpc.2013.09.018.
(27) Jarzynski, C. Nonequilibrium Equality for Free Energy Differences. *Phys. Rev. Lett.* **1997**, *78* (14), 2690–2693. https://doi.org/10.1103/PhysRevLett.78.2690.
(28) Park, S.; Khalili-Araghi, F.; Tajkhorshid, E.; Schulten, K. Free Energy Calculation from Steered Molecular Dynamics Simulations Using Jarzynski's Equality. *The Journal of Chemical Physics* **2003**, *119* (6), 3559–3566. https://doi.org/10.1063/1.1590311.
(29) Kästner, J. Umbrella Sampling. *WIREs Computational Molecular Science* **2011**, *1* (6), 932–942. https://doi.org/10.1002/wcms.66.
(30) Bussi, G.; Laio, A. Using Metadynamics to Explore Complex Free-Energy Landscapes. *Nat Rev Phys* **2020**, *2* (4), 200–212. https://doi.org/10.1038/s42254-020-0153-0.
(31) Ma, C. D.; Wang, C.; Acevedo-Vélez, C.; Gellman, S. H.; Abbott, N. L. Modulation of Hydrophobic Interactions by Proximally Immobilized Ions. *Nature* **2015**, *517* (7534), 347–350. https://doi.org/10.1038/nature14018.
(32) Acevedo-Vélez, C.; Andre, G.; Dufrêne, Y. F.; Gellman, S. H.; Abbott, N. L. Single-Molecule Force Spectroscopy of β-Peptides That Display Well-Defined Three-Dimensional Chemical Patterns. *J. Am. Chem. Soc.* **2011**, *133* (11), 3981–3988. https://doi.org/10.1021/ja1089183.
(33) Pomerantz, W. C.; Cadwell, K. D.; Hsu, Y.-J.; Gellman, S. H.; Abbott, N. L. Sequence Dependent Behavior of Amphiphilic β-Peptides on Gold Surfaces. *Chem. Mater.* **2007**, *19* (18), 4436–4441. https://doi.org/10.1021/cm070265d.
(34) Qiu, C.; Whittaker, G. R.; Gellman, S. H.; Daniel, S.; Abbott, N. L. Interactions of SARS-CoV-2 and MERS-CoV Fusion Peptides Measured Using Single-Molecule Force Methods. *Biophys J* **2023**, *122* (4), 646–660. https://doi.org/10.1016/j.bpj.2023.01.016.
(35) Lostao, A.; Lim, K.; Pallarés, M. C.; Ptak, A.; Marcuello, C. Recent Advances in Sensing the Inter-Biomolecular Interactions at the Nanoscale – A Comprehensive Review of AFM-Based Force Spectroscopy. *International Journal of Biological Macromolecules* **2023**, *238*, 124089. https://doi.org/10.1016/j.ijbiomac.2023.124089.
(36) Rankl, C.; Kienberger, F.; Gruber, H.; Blaas, D.; Hinterdorfer, P. Accuracy Estimation in Force Spectroscopy Experiments. *Jpn. J. Appl. Phys.* **2007**, *46* (8S), 5536. https://doi.org/10.1143/JJAP.46.5536.
(37) Hartigan, J. A.; Hartigan, P. M. The Dip Test of Unimodality. *The Annals of Statistics* **1985**, *13* (1), 70–84. https://doi.org/10.1214/aos/1176346577.
(38) Saleem, J.; Baig, M. Z. K.; Luyt, A. S.; Shakoor, R. A.; Zekri, A.; McKay, G. Free-Standing Polypropylene Porous Thin Films Using Energy Efficient Coating Technique. *Energy Reports* **2023**, *9*, 31–39. https://doi.org/10.1016/j.egyr.2022.12.096.
(39) Wei, Y.; Latour, R. A. Correlation between Desorption Force Measured by Atomic Force Microscopy and Adsorption Free Energy Measured by Surface Plasmon Resonance Spectroscopy for Peptide − Surface Interactions. *Langmuir* **2010**, *26* (24), 18852–18861. https://doi.org/10.1021/la103685d.
(40) Duanis-Assaf, T.; Hu, T.; Lavie, M.; Zhang, Z.; Reches, M. Understanding the Adhesion Mechanism of Hydroxyapatite-Binding Peptide. *Langmuir* **2022**, *38* (3), 968–978. https://doi.org/10.1021/acs.langmuir.1c02293.

(41) Thyparambil, A. A.; Wei, Y.; Latour, R. A. Determination of Peptide–Surface Adsorption Free Energy for Material Surfaces Not Conducive to SPR or QCM Using AFM. *Langmuir* **2012**, *28* (13), 5687–5694. https://doi.org/10.1021/la300315r.

(42) Eskhan, A.; AlQasas, N.; Johnson, D. Interaction Mechanisms and Predictions of the Biofouling of Polymer Films: A Combined Atomic Force Microscopy and Quartz Crystal Microbalance with Dissipation Monitoring Study. *Langmuir* **2023**, *39* (18), 6592–6612. https://doi.org/10.1021/acs.langmuir.3c00587.

(43) Zhu, F.; Hummer, G. Convergence and Error Estimation in Free Energy Calculations Using the Weighted Histogram Analysis Method. *Journal of Computational Chemistry* **2012**, *33* (4), 453–465. https://doi.org/10.1002/jcc.21989.

(44) Dallin, B. C.; Yeon, H.; Ostwalt, A. R.; Abbott, N. L.; Van Lehn, R. C. Molecular Order Affects Interfacial Water Structure and Temperature-Dependent Hydrophobic Interactions between Nonpolar Self-Assembled Monolayers. *Langmuir* **2019**, *35* (6), 2078–2088. https://doi.org/10.1021/acs.langmuir.8b03287.

(45) Miller, C. A.; Abbott, N. L.; de Pablo, J. J. Surface Activity of Amphiphilic Helical β-Peptides from Molecular Dynamics Simulation. *Langmuir* **2009**, *25* (5), 2811–2823. https://doi.org/10.1021/la802973e.

(46) Dalgicdir, C.; Globisch, C.; Peter, C.; Sayar, M. Tipping the Scale from Disorder to Alpha-Helix: Folding of Amphiphilic Peptides in the Presence of Macroscopic and Molecular Interfaces. *PLOS Computational Biology* **2015**, *11* (8), e1004328. https://doi.org/10.1371/journal.pcbi.1004328.

(47) MacKay, D. J. C. *Information Theory, Inference, and Learning Algorithms*; University Press: Cambridge, 2004. https://doi.org/10.2277/0521642981.

(48) Pfaendtner, J.; Bonomi, M. Efficient Sampling of High-Dimensional Free-Energy Landscapes with Parallel Bias Metadynamics. *J. Chem. Theory Comput.* **2015**, *11* (11), 5062–5067. https://doi.org/10.1021/acs.jctc.5b00846.

(49) Qi, X.; Pfaendtner, J. High-Throughput Computational Screening of Solid-Binding Peptides. *J. Chem. Theory Comput.* **2024**, *20* (7), 2959–2968. https://doi.org/10.1021/acs.jctc.3c01286.

(50) Branduardi, D.; Bussi, G.; Parrinello, M. Metadynamics with Adaptive Gaussians. *J. Chem. Theory Comput.* **2012**, *8* (7), 2247–2254. https://doi.org/10.1021/ct3002464.

(51) Raiteri, P.; Laio, A.; Gervasio, F. L.; Micheletti, C.; Parrinello, M. Efficient Reconstruction of Complex Free Energy Landscapes by Multiple Walkers Metadynamics. *J. Phys. Chem. B* **2006**, *110* (8), 3533–3539. https://doi.org/10.1021/jp054359r.
